# Proteomic Studies on the Mechanism of Myostatin Regulating Cattle Skeletal Muscle Development

**DOI:** 10.3389/fgene.2021.752129

**Published:** 2021-11-16

**Authors:** Hui Sheng, Yiwen Guo, Linlin Zhang, Junxing Zhang, Manning Miao, Haoyun Tan, Debao Hu, Xin Li, Xiangbin Ding, Guangpeng Li, Hong Guo

**Affiliations:** ^1^ Tianjin Key Laboratory of Agricultural Animal Breeding and Healthy Husbandry, College of Animal Science and Veterinary Medicine, Tianjin Agricultural University, Tianjin, China; ^2^ The Key Laboratory of Mammalian Reproductive Biology and Biotechnology of the Ministry of Education, Inner Mongolia University, Hohhot, China

**Keywords:** myostatin, proteomics, extracellular matrix, ribosome, focal adhesion

## Abstract

Myostatin (MSTN) is an important negative regulator of muscle growth and development. In this study, we performed comparatively the proteomics analyses of gluteus tissues from MSTN^+/−^ Mongolian cattle (MG.MSTN^+/−^) and wild type Mongolian cattle (MG.WT) using a shotgun-based tandem mass tag (TMT) 6-plex labeling method to investigate the regulation mechanism of MSTN on the growth and development of bovine skeletal muscle. A total of 1,950 proteins were identified in MG.MSTN^+/−^ and MG.WT. Compared with MG.WT cattle, a total of 320 differentially expressed proteins were identified in MG.MSTN cattle, including 245 up-regulated differentially expressed proteins and 75 down-regulated differentially expressed proteins. Bioinformatics analysis showed that knockdown of the MSTN gene increased the expression of extracellular matrix and ribosome-related proteins, induced activation of focal adhesion, PI3K-AKT, and Ribosomal pathways. The results of proteomic analysis were verified by muscle tissue Western blot test and *in vitro* MSTN gene knockdown test, and it was found that knockdown MSTN gene expression could promote the proliferation and myogenic differentiation of bovine skeletal muscle satellite cells (BSMSCs). At the same time, Co-Immunoprecipitation (CO-IP) assay showed that MSTN gene interacted with extracellular matrix related protein type I collagen α 1 (COL1A1), and knocking down the expression of COL1A1 could inhibit the activity of adhesion, PI3K-AKT and ribosome pathway, thus inhibit BSMSCs proliferation. These results suggest that the MSTN gene regulates focal adhesion, PI3K-AKT, and Ribosomal pathway through the COL1A1 gene. In general, this study provides new insights into the regulatory mechanism of MSTN involved in muscle growth and development.

## Introduction

Myostatin (MSTN), also known as growth differentiation factor-8 (GDF-8), is a highly conservative member of the transforming growth factor β (TGF-β) superfamily (McPherron et al., 1997). In previous studies, MSTN has been confirmed to be a secreted growth factor expressed predominantly in skeletal muscle (McPherron, 1997; Kollias and McDermott, 2008) and plays a key role in the negative regulation of muscle development (Tsuchida, 2008). *In vitro* and *in vivo* studies have shown that the Mstn signal is mediated by binding to activin receptor type-IIB (ActRIIB). Mstn has also been shown to bind to ActRIIB, although the functional correlation of this interaction has not been fully determined (Lee and McPherron, 2001). MSTN deficiency resulting from genetic ablation of both copies (MSTN^−/-^) or single copy (MSTN^+/−^) of the germline allele or loss-of-function mutations usually leads to a “double muscle” phenotype, mainly characterized by a significant increase in muscle mass (McPherron, 1997; McPherron et al., 1997). Similar phenotypes were also demonstrated in natural mutations of MSTN genes in cattle (Grobet et al., 1997; McPherron, 1997), sheep (Clop et al., 2006), dogs (Mosher et al., 2007), and humans (Schuelke et al., 2004). On the contrary, overexpression of MSTN or systemic administration can lead to muscle atrophy (Zimmers et al., 2002; Durieux et al., 2007). All these effects are mainly achieved by regulating the proliferation and differentiation of myoblasts (Thomas et al., 2000; Rios et al., 2001; Langley et al., 2002; Rios et al., 2002). MSTN has been shown to interfere with proliferation and protein synthesis and protein decomposition of adult muscle fibers (Sartori et al., 2009; Trendelenburg et al., 2009). Many studies have shown that MSTN can regulate IGF-I signal pathway (Morissette et al., 2009), WNT4/β-catenin signal pathway (Steelman et al., 2006), Erk1/2, c-Jun N-terminal kinase (JNK) signal pathway, p38 mitogen-activated protein (MAP)K (Allendorph et al., 2006; Huang et al., 2007; Joulia-Ekaza and Cabello, 2007; Elkasrawy and Hamrick, 2010), and PI3K/Akt signal pathway (Lipina et al., 2010) through transforming growth factor-β pathway (Kollias and McDermott, 2008; Elkasrawy and Hamrick, 2010). Besides, muscle somatostatin mediates CKD-induced muscle catabolism by coordinating the activation of autophagy and the ubiquitin-proteasome system and may limit cell proliferation by activating miRNAs (Wang et al., 2015; Huang et al., 2019). In addition to its effects on muscle development, MSTN also plays an important role in fat and glucose metabolism. The results of previous studies in our laboratory showed that knockout MSTN gene expression enhanced glycolysis and fat β-oxidation in bovine muscle tissue (Yang et al., 2018; Xin et al., 2020). High levels of MSTN have been shown to cause muscle atrophy and are associated with a variety of diseases, such as cancer (Aversa et al., 2012), chronic obstructive pulmonary disease (Hayot et al., 2011), chronic heart failure (Lenk et al., 2009), acquired immune deficiency syndrome (Gonzalez-Cadavid et al., 1998), obesity, insulin resistance, and type 2 diabetes (McPherron and Lee, 2002; Zhang et al., 2011; Zhang et al., 2012). Therefore, MSTN dysfunction has been considered a promising strategy for animal breeding or for fighting muscle atrophy in different diseases, including neuromuscular diseases (Mariot et al., 2017). Although MSTN signal cascade plays a central role in regulating muscle weight, the mechanism of this signal cascade is still unclear (Elkina et al., 2011).

Proteins play an important role in many types of molecular networks and perform most of the biochemical functions of organisms. Label-free liquid chromatography-mass spectrometry (LC-MS/MS) can be used to quantify and identify thousands of proteins in multiple samples in one operation, which provides an unprecedented opportunity to study the proteomic changes of biological components or organisms (Wang et al., 2019). Protein de/phosphorylation is a ubiquitous post-translational modification, which plays a regulatory role in protein structure, function, cell signal transduction, and enzyme activity regulation. Phosphorylation usually occurs on serine, threonine, and tyrosine residues and is catalyzed by upstream protein kinases by adding covalently bound phosphate groups (Chen et al., 2019). Some studies have shown that reversible protein phosphorylation plays an important role in the transformation of muscle to meat by regulating the development of meat quality by regulating proteins involved in glycolysis and muscle contraction (Huang et al., 2011; Huang et al., 2012; Li et al., 2012; Huang et al., 2014; Li et al., 2015a; Li et al., 2015b; Chen et al., 2016). Protein phosphorylation of various muscle samples has been reported, for example, myosin and myosin regulatory light chain 2 have been identified (Obermann et al., 1997; Muroya et al., 2007). Because there are many proteins in the pathway affected by MSTN, the single-target study can not fully analyze its function and mechanism, so proteomics and detection of the phosphorylation level of key proteins in signal pathway can be used to comprehensively and effectively analyze its mechanism or regulatory proteins.

In this study, in order to explore the mechanism of MSTN gene regulating signal pathway during muscle development, we used 6-plex TMT labeling method to determine the global protein abundance in the gluteal muscle tissue of MG.MSTN^+/−^ cattle with artificially knocked down expression of MSTN gene and MG.WT cattle with normal expression of MSTN gene. These proteomic analyses enable us to compare quantitative changes in overall protein abundance. After statistical and bioinformatics analysis, based on the results of protein abundance changes related to MSTN gene deletion, we carried out Western blot tests of muscle tissue samples and *in vitro* experiments and confirmed that the data sets from global proteomics are reliable.

## Results

### Statistical Analysis of Mass Spectrometry Results

MSTN^+/−^ associated changes in abundance of any identified proteins were determined based on the TMT 6-plex reporter ion ratios. The results of mass spectrometry analysis showed that a total of 1,950 quantitative proteins were identified in the gluteal muscle tissues of MG.MSTN^+/−^ cattle and MG.WT cattle, and each protein contained at least two unique peptides (Supplement Table 3A). After scatter plotting analysis (Supplementary Figure S1) used for determining the internal error of the biological replicates and student t-test analysis of the data set (Supplement Table 3B), the fold change in values more than 1.3 were determined based on the value of the log2 TMT ratio (log2 1.3 = 0.38) at which 95% of all proteins had no deviation (Martin et al., 2016). Thus, the fold-change (≥1.3) and *p*-value (≤0.05) from the *t*-test were applied to rank and filter the quantitative data. The proteins with fold change ≥1.30 or ≤0.77 in relative abundance and a *p*-value ≤ 0.05 were identified as differentially expressed proteins (Supplement Table 3B).

### Identification of Differentially Expressed Proteins Between MG.MSTN^+/−^ and MG.WT Cattle

**FIGURE 1:**
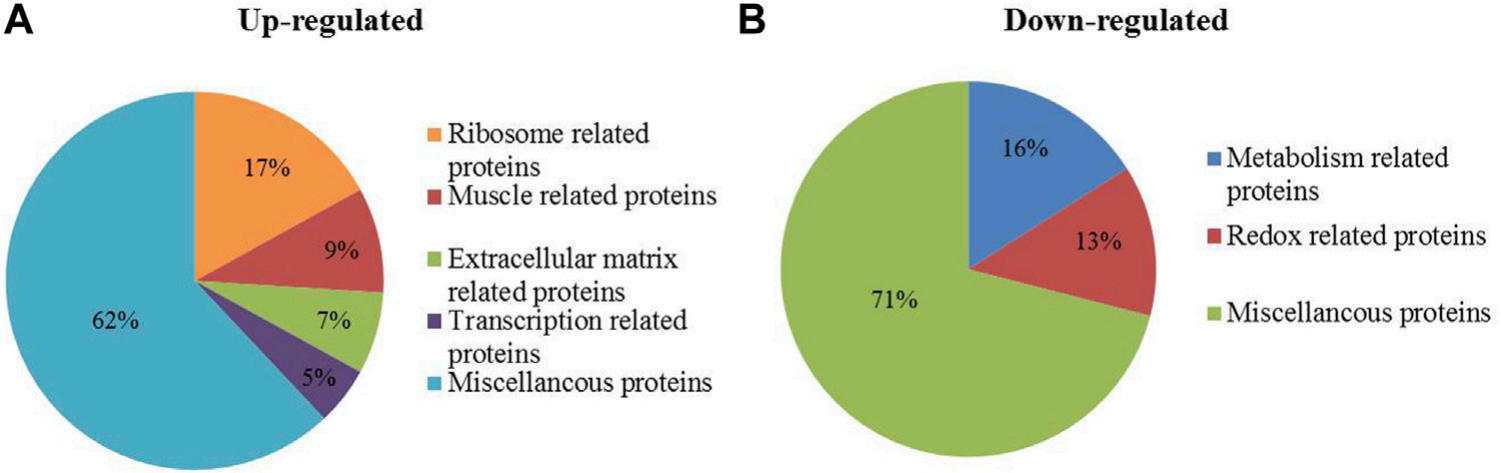
The biological functions of 320 differentially expressed proteins identified in MG.MSTN+/− vs MG.WT data. **(A, B)** show the proportions of biological functions among 245 up-regulated proteins and 75 down-regulated proteins.

Compared with wild MG.WT, 320 differentially expressed proteins were identified in MG.MSTN^+/−^ group, including 245 proteins with increased abundance and 75 proteins with decreased abundance (Supplementary Table S3), which were used in subsequent bioinformatics analysis and verification experiments. GO analysis showed that the functions of 245 up-regulated proteins were divided into ribosome-related proteins (17%), muscle-related proteins (9%), extracellular matrix-related proteins (7%), and transcription-related proteins (5%) (Figure 1A). While the 75 down-regulated proteins were classified as metabolism-related proteins (16%) and redox-related proteins (13%) (Figure 1B). Interestingly, we found that the expression abundance of a large number of extracellular matrix related proteins (such as collagen alpha-1(I) chain, laminin subunit beta 1) and ribosomal related proteins (such as 40S ribosomal protein S6, 60S ribosomal protein L26) increased in MG.MSTN^+/−^ (Table 1). This result is expected to activate focal adhesion, PI3K-AKT, and ribosomal pathways, enhance actin-binding and protein synthesis, and promote muscle contraction and growth.

**TABLE 1:** A partial list of the differentially expressed proteins involving extracellular matrix related proteins and ribosomal related in MG.MSTN^+/−^ vs MG.WT

Protein accession	Protein description	Protein name	Fold-change (MG.MSTN^+/−^ vs MG.WT)	Regulated
**Extracellular matrix related proteins**				
77404252	collagen alpha-1(I) chain precursor	COL1A1	2.32	Up
27806257	collagen alpha-2(I) chain precursor	COL1A2	2.95	Up
528938209	PREDICTED: collagen alpha-2(VI) chain isoform X1	COL6A2	1.81	Up
528945453	PREDICTED: collagen alpha-3(VI) chain isoform X2	COL6A3	2.26	Up
982945253	PREDICTED: collagen alpha-1 (XIV) chain isoform X1	COL14A1	1.66	Up
116003881	collagen alpha-1(III) chain precursor	COL3A1	1.52	Up
300796391	collagen alpha-1 (XV) chain precursor	COL15A1	1.43	Up
982987742	PREDICTED: laminin subunit alpha-2, partial	LAMA2	2.03	Up
329664360	laminin subunit alpha-4 precursor	LAMA4	1.93	Up
330688474	laminin subunit beta-1 precursor	LAMB1	2.06	Up
332205887	laminin subunit gamma-1 precursor	LAMC1	1.62	Up
982993396	PREDICTED: LOW QUALITY PROTEIN: laminin subunit alpha-5 isoform X2	LAMA5	0.58	Down
78045497	vitronectin precursor	VTN	0.70	Down
**Ribosomal related proteins**				
66792868	40S ribosomal protein S15	RPS15	1.85	Up
528974050	PREDICTED: 40S ribosomal protein S21 isoform X1	RPS21	1.68	Up
77797830	40S ribosomal protein S10	RPS10	1.66	Up
70778778	40S ribosomal protein S13	RPS13	1.57	Up
529010831	PREDICTED: 40S ribosomal protein S24 isoform X1	RPS24	1.56	Up
75812924	40S ribosomal protein S18	RPS18	1.53	Up
82697365	40S ribosomal protein S19	RPS19	1.53	Up
149642623	40S ribosomal protein S7	RPS7	1.47	Up
62752040	40S ribosomal protein S6	RPS6	1.38	Up
149642675	40S ribosomal protein S17	RPS17	1.36	Up
62461611	40S ribosomal protein S12	RPS12	1.36	Up
155372029	40S ribosomal protein S9	RPS9	1.34	Up
70778964	40S ribosomal protein S25	RPS25	1.32	Up
70778960	40S ribosomal protein S28	RPS28	1.31	Up
741965251	PREDICTED: 28S ribosomal protein S27, mitochondrial isoform X1	MRPS27	1.71	Up
982996641	PREDICTED: 60S ribosomal protein L22 isoform X1	RPL22	1.64	Up
62751887	60S ribosomal protein L26	RPL26	1.58	Up
77404275	60S ribosomal protein L27	RPL27	1.53	Up
70778766	60S ribosomal protein L31	RPL31	1.48	Up
77735941	60S ribosomal protein L35	RPL35	1.48	Up
27806129	60S ribosomal protein L24	RPL24	1.45	Up
114051890	60S ribosomal protein L23a	RPL23A	1.45	Up
528936538	PREDICTED: 60S ribosomal protein L35a isoform X1	RPL35A	1.44	Up
27807523	60S acidic ribosomal protein P2	RPLP2	1.42	Up
529001374	PREDICTED: 60S ribosomal protein L29 isoform X1	RPL29	1.40	Up
78042478	60S ribosomal protein L37a	RPL37A	1.38	Up
528993469	PREDICTED: 60S ribosomal protein L28 isoform X1	RPL28	1.37	Up
528988589	PREDICTED: 60S ribosomal protein L6 isoform X1	RPL6	1.35	Up
62751646	60S ribosomal protein L13	RPL13	1.33	Up
62460552	60S ribosomal protein L7	RPL7	1.32	Up
94966839	60S ribosomal protein L7a	RPL7A	1.32	Up

Note: Corrected *p*-value ≤ 0.05, Fold change ≥1.30 or ≤0.77.

### Functional Analysis of Differentially Expressed Proteins Between MG.MSTN^+/−^ and MG.WT Cattle

**FIGURE 2:**
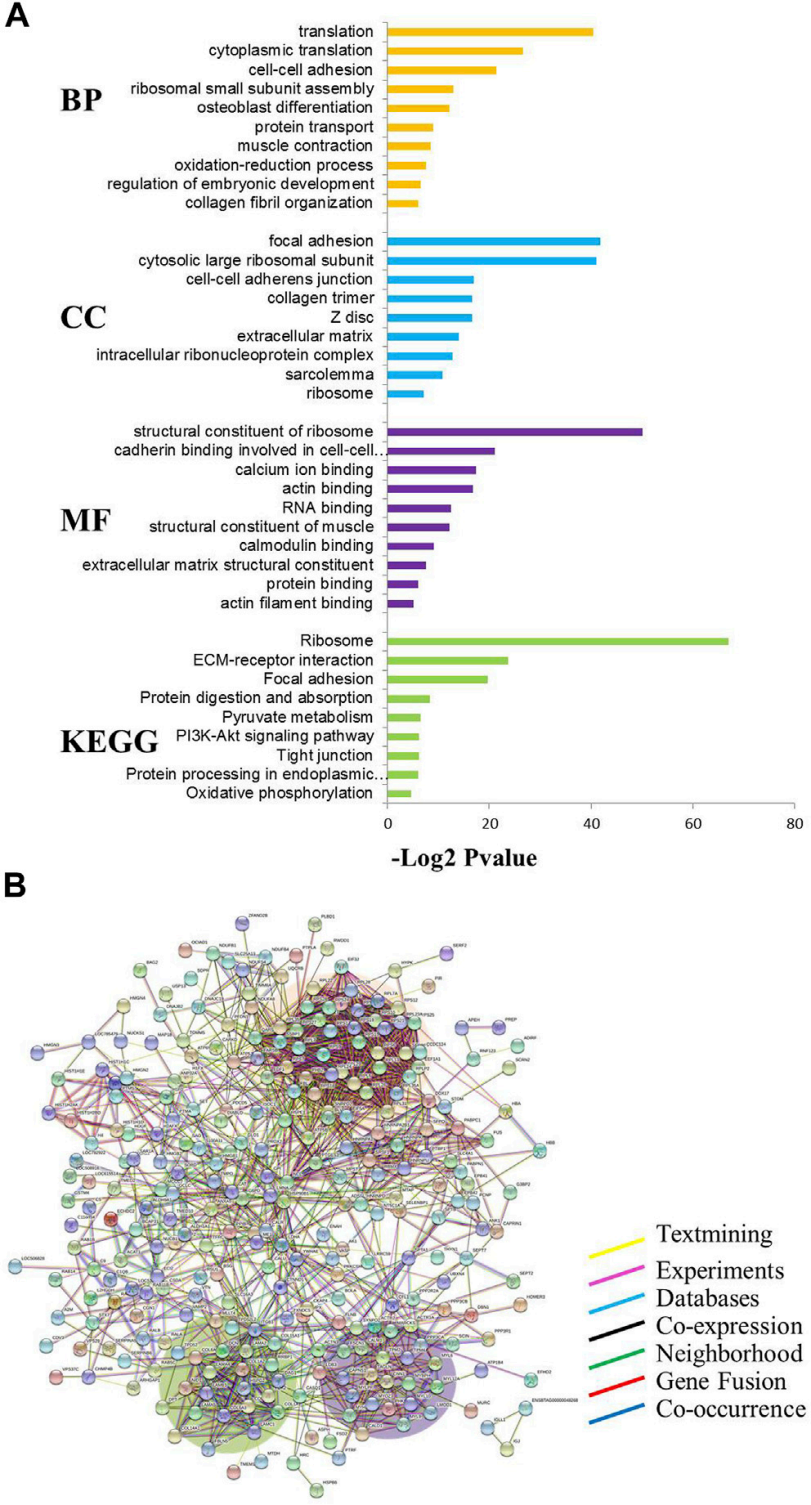
Functional classification of differentially expressed proteins in MG.MSTN+/− vs MG.WT. **(A)** GO and KEGG analyses. BP: Biological process, CC: Cellular component, MF: Molecular function, KEGG: Kyoto encyclopedia of genes and genomes. The values given in each of the enriched terms or pathways are corrected at *p* ≤ 0.01. **(B)** Protein-protein interaction analysis.

The results of the GO analysis are shown in Figure 2A with *p* ≤ 0.01 as a significant threshold. In the GO annotation and KEGG analysis of 320 differentially expressed proteins, the translation, focal adhesion, and structural constituent of ribosome were significantly enriched as the primary categories of biological process (BP), cellular component (CC), and molecular functional (MF), respectively. At the same time, ribosome, ECM receptor interaction, focal adhesion, and other pathways have also been greatly enriched in the current KEGG database. Using the STRING database to further analyze the possible protein-protein interaction networks of these changed proteins, it was found that there were three major protein-protein interaction networks among the 320 differentially expressed proteins (Figure 2B). The largest network highlighted with an pink background in Figure 2B includes more than 60 proteins known to be associated with translation (such as eukaryotic translation initiation factor 5A-1 and 40S ribosomal protein S21). The second network with a violet background contains about 30 proteins including myosin light polypeptide 6 and is mainly involved in muscle contraction. The third network with a green background includes about 20 extracellular matrix related proteins (such as collagen alpha-1(I) chain).

### Tissue Samples Western Blot to Verify the Accuracy of Data Analysis

**FIGURE 3:**
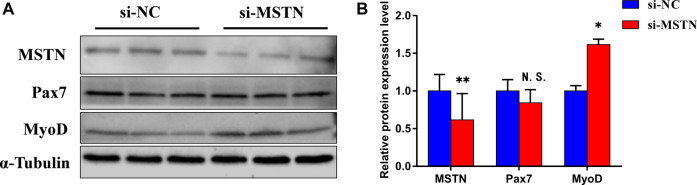
Comparison and verification of quantitative results of phosphorylation levels of differentially expressed proteins and key proteins in the pathway from Western blotting analysis. **(A)** Western blotting results of MSTN,MYL6, pFAK (Tyr-473), pAKT1 (Ser-473) and pRPS6 (Ser-235/236) proteins. **(B)** Results of muscle tissue protein Western blot quantification. These imprints are cropped and the original image is shown in Supplementary Figure S2.

By comparing the proteomic data of the gluteal muscle of MG.MSTN^+/−^ and MG.WT, it was found that knocking down the expression of MSTN increased the expression of a large number of extracellular matrix related proteins and ribosomal related proteins. And these proteins are important components of focal adhesion, PI3K-AKT, and ribosomal pathway. Therefore, we speculate that knocking down the expression of the MSTN gene may activate focal adhesion, PI3K-AKT, and ribosomal pathway. To verify the TMT-based quantitative proteomics results, we used classical Western blot analyses to validate the accuracy of data analysis. Considering the availability of some antibodies, we performed Western blot analysis of MSTN, one differentially expressed protein (MYL6), and alpha-tubulin (for internal control). As shown in Figure 3, the expression trend of Western blot results is consistent with that of data analysis (supplement Table 3), indicating that quantitative proteomics data have reasonable accuracy. At the same time, the expressions of key proteins pFAK, pAKT, and pRPS6 in focal adhesion, PI3K-AKT, and ribosomal pathway were verified by Western blot. The results showed that the expression of pFAK, pAKT, and pRPS6 in the gluteal muscle of MG.MSTN^+/−^ was significantly higher than that of WT Mongolian cattle (Figure 3), which was consistent with our conjecture.

### Knockdown of Myostatin Expression Promotes Proliferation and Myogenic Differentiation of Bovine Skeletal Muscle Satellite Cells

**FIGURE 4:**
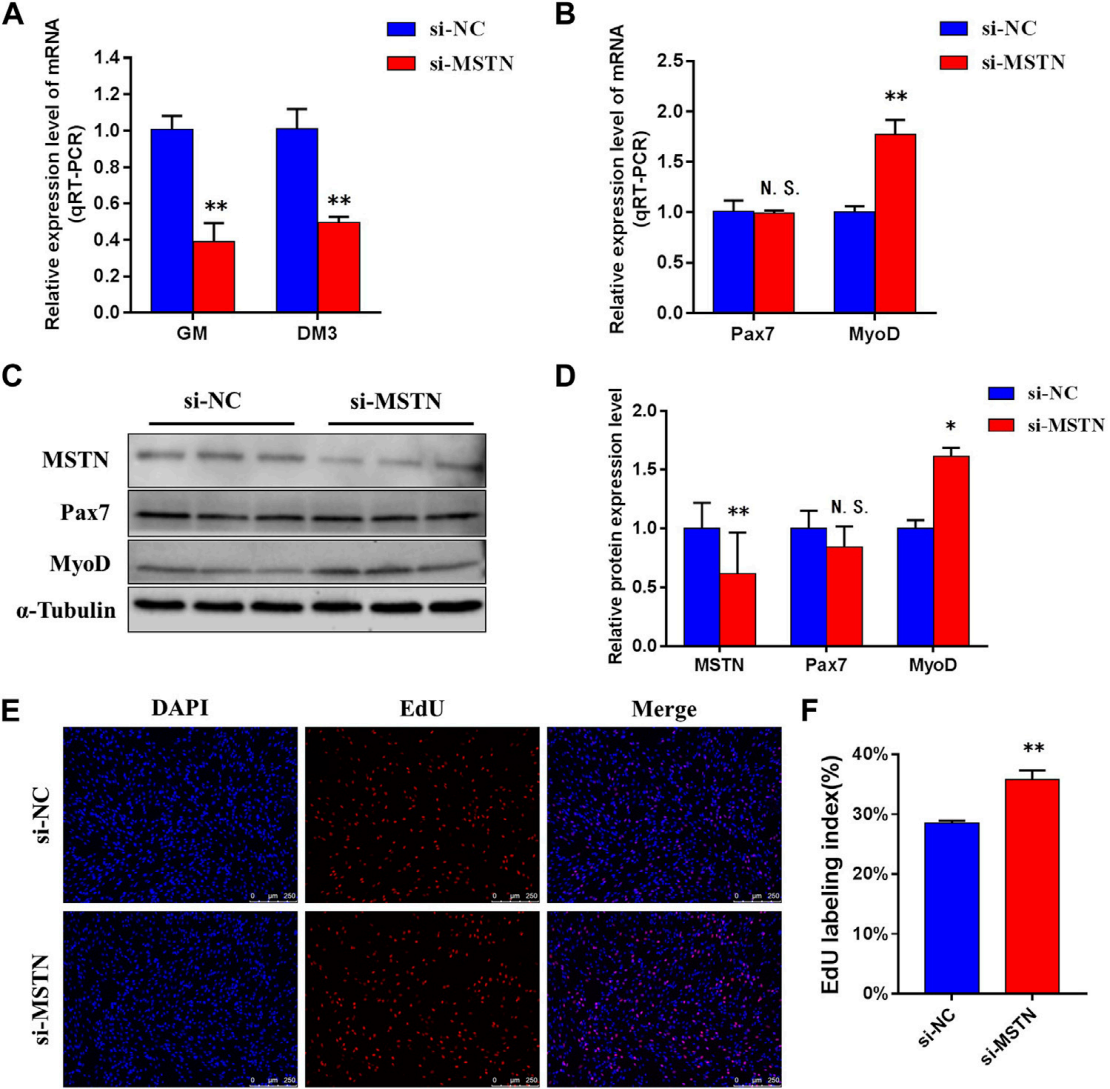
Knockdown of MSTN expression promotes the maintenance of bovine skeletal muscle satellite cells (BSMSCs) proliferation. **(A)** Knockdown MSTN significantly decreased MSTN expression level in proliferative phase (GM) and the third day of differentiation (DM3). **(B)** resulting in no change in the expression level of mRNA of Pax7, but significantly increased the expression level of mRNA of MyoD. **(C–D)** no significant change of protein expression of Pax7, and significant increase of protein expression of MyoD. (E–F) EdU assay was performed at 24 h after transfection. The number of 5-ethynyl-2′-deoxyuridine (EdU)-positive cells (E, ×200; scale bars 250 μm) and EdU labeling index increased with MSTN knockdown. These blots were cropped and original images were shown in Supplementary Figure S3.

**FIGURE 5:**
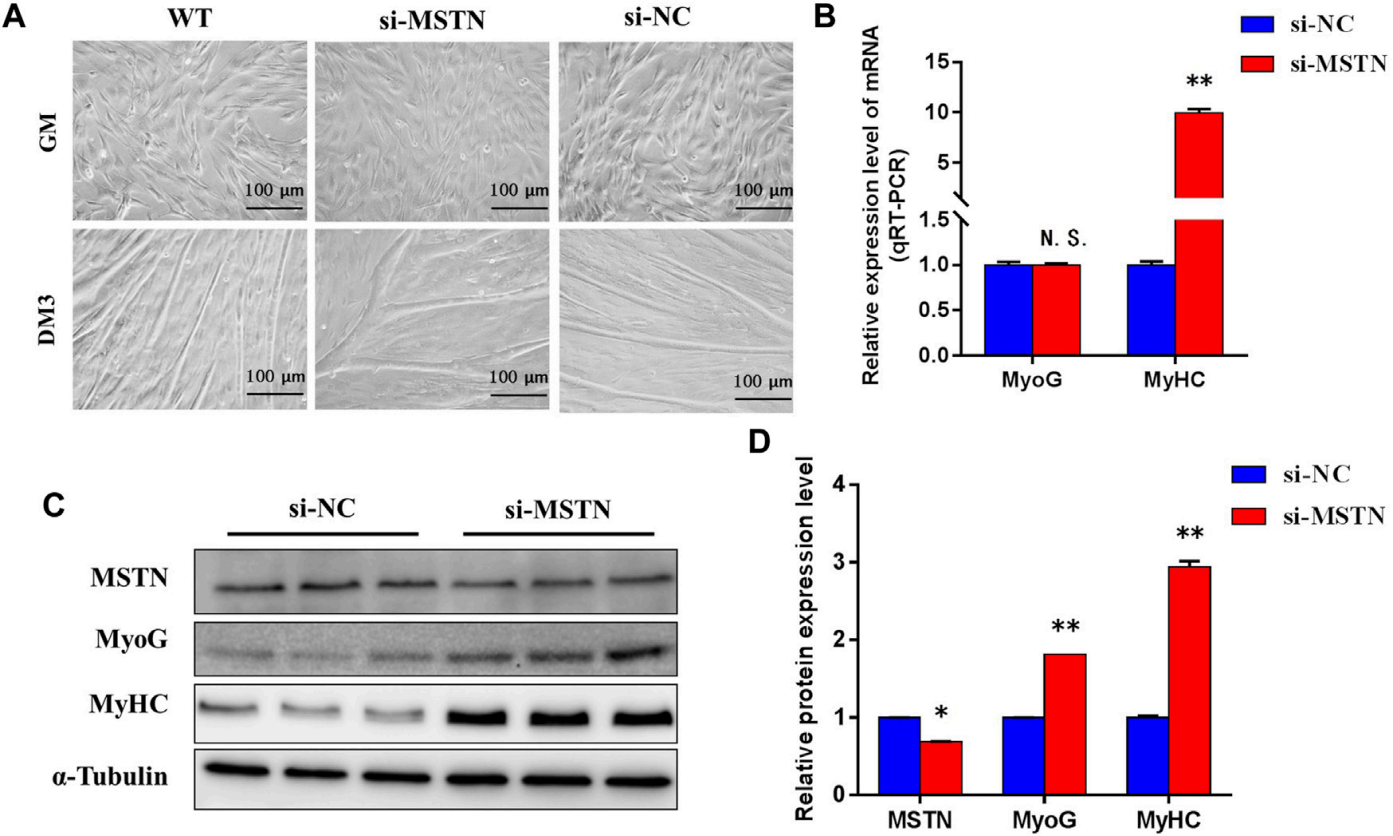
MSTN functions in the differentiation of bovine myoblasts. **(A)** Knockdown of MSTN expression promoted the differentiation process of bovine myoblasts (×200; scale bars 100 μm). **(B)** Knockdown experiments resulted in no change in the mRNA expression level of MyoG, but increased the expression level of mRNA of MyHC. **(C–D)** The protein expression levels of MyoG and MHC were significantly increased. These blots were cropped and original images were shown in Supplementary Figure S4.

To explore the mechanism of the effect of the MSTN gene on the development of bovine skeletal muscle, this experiment studied the effect of MSTN knockdown on the proliferation and myogenic differentiation of BSMSCs. SiRNA (si-MSTN) for MSTN knockdown was designed and synthesized which resulted in a significant decrease in the expression of MSTN in cells GM and DM3 respectively (Figure 4A). Meanwhile, proliferation-related markers Pax7 and MyoD were detected to evaluate whether cell proliferation was affected. The results showed that the expression of MSTN was silenced, the mRNA expression levels of Pax7 did not change significantly, the mRNA expression levels of MyoD was significantly up-regulated (Figure 4B); the protein expression levels of Pax7 did not change significantly, and the protein expression levels of MyoD was significantly up-regulated (Figure 4C). The results of the 5-ethynyl-2′-deoxyuridine (EdU) cell proliferation assay showed that when MSTN was knocked down, the number of EDU positive cells (Figure 4D) and EDU labeling index (Figure 4E) were all up-regulated. Meanwhile, the differentiation process of satellite cells was observed under the light microscope. Compared with the wild group (WT) and control group (si-NC), when MSTN was knocked down, thick myotubes were formed in DM3 (Figure 5A). The expression levels of differentiation-related markers MyoG and MyHC were detected. The results showed that the expression of MSTN was silenced, the mRNA expression level of MyoG did not change significantly, but the mRNA expression level of MyHC was significantly up-regulated (Figure 5B); the protein expression levels of MyoG and MyHC were all significantly up-regulated (Figures 5C,D). To sum up, our experiments showed that knocking down the expression of the MSTN gene promotes the proliferation and myogenic differentiation of BSMSCs.

### Knockdown of Myostatin Expression Activates Focal Adhesion, PI3K-AKT, and Ribosomal Pathway

**FIGURE 6:**
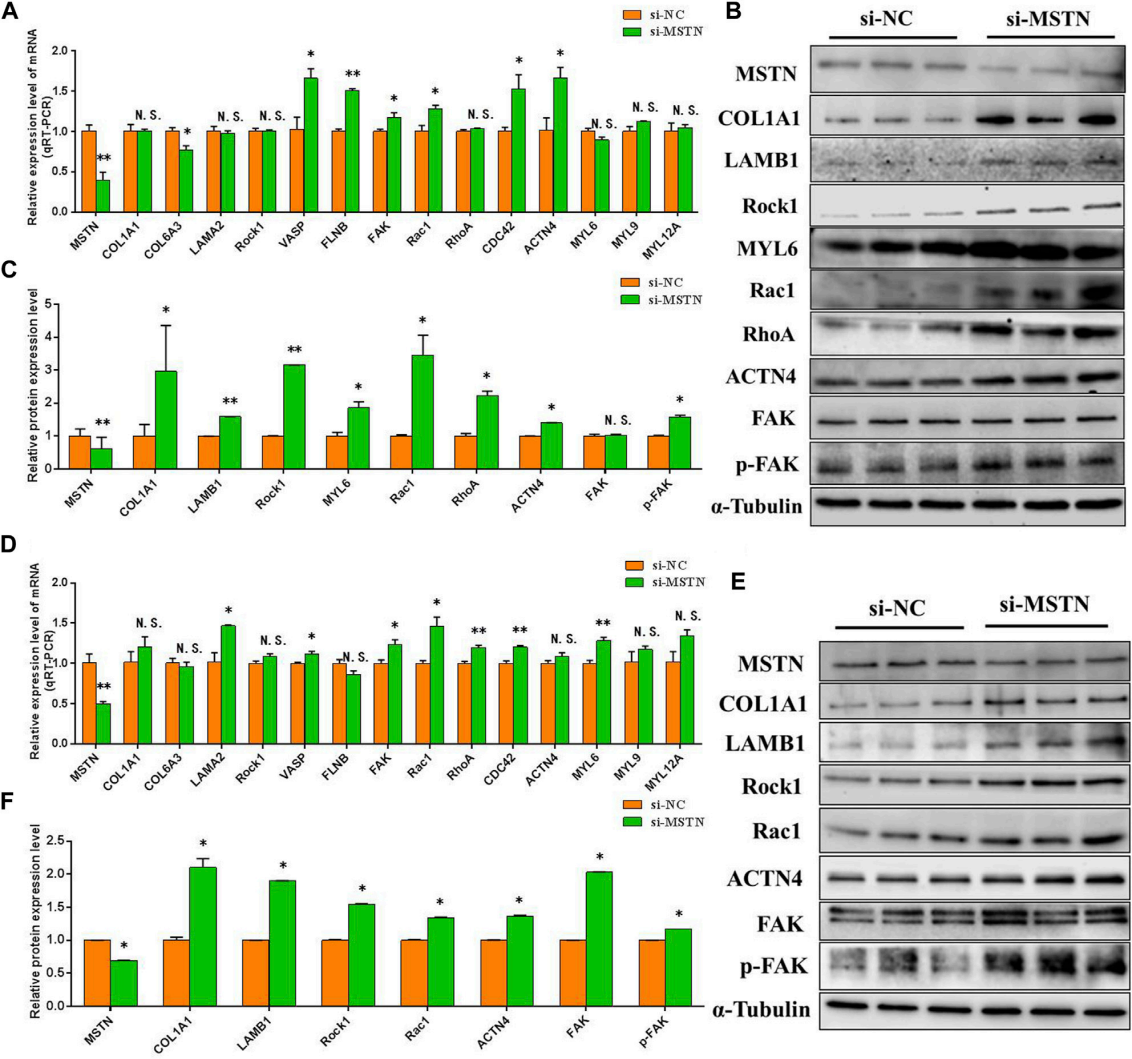
The effect of MSTN on the focal adhesion pathway. **(A–C)** Knockdown of MSTN expression resulted in a significant increase in the mRNA expression level and protein expression level of the majority of differentially expressed proteins involved in the focal adhesion pathway in GM, as well as the expression level of the key gene FAK and p-FAK in the pathway. **(D–F)** The mRNA expression level and protein expression level of these proteins were also generally increased in DM3. These blots were cropped and original images were shown in Supplementary Figure S5 and Figure 6.

**FIGURE 7:**
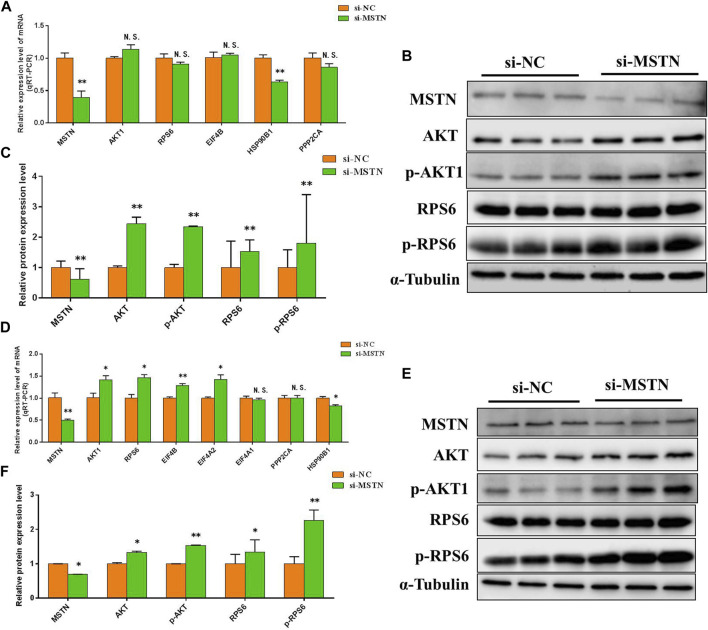
The effect of MSTN on the PI3K-AKT and ribosomal pathway. **(A–C)** Knockdown of MSTN expression resulted in a significant increase in mRNA expression level and protein expression level of majority differentially expressed proteins involved in PI3K-AKT and ribosomal pathway in GM, as well as a significant increase in the expression of key genes AKT1, RPS6, p-AKT1and p-RPS6 in the pathway. **(D–F)** The mRNA expression level and protein expression level of these proteins were also generally increased in DM3. These blots were cropped and original images were shown in Supplementary Figure S7 and Figure 8.

To further verify the accuracy of the data analysis results, to explore the effects of the MSTN gene on focal adhesion, PI3K-AKT, and ribosomal pathway. The RNA and protein were extracted from GM and DM3 cells transfected with si-MSTN and were used to detect the differentially expressed proteins involved in focal adhesion, PI3K-AKT, and ribosomal pathways in the gluteal muscles of MG.MSTN^+/−^ and MG.WT, as well as the expression levels of key genes FAK, AKT, RPS6, pFAK, pAKT, and pRPS6 in these pathways. The results showed that the mRNA expression level and protein expression level of these differentially expressed proteins, as well as FAK, AKT, and RPS6 were mostly up-regulated in GM and DM3 cells silenced by MSTN. Meanwhile, it was found that the protein expression levels of p-FAK, p-AKT, and p-RPS6 were also significantly increased (Figure 6, Figure 7). The test results are consistent with speculation, which proves that knocking down the MSTN gene expression can activate focal adhesion, PI3K-AKT, and ribosomal pathways.

### Verification of Interaction Between Myostatin and COL1A1

**FIGURE 8:**
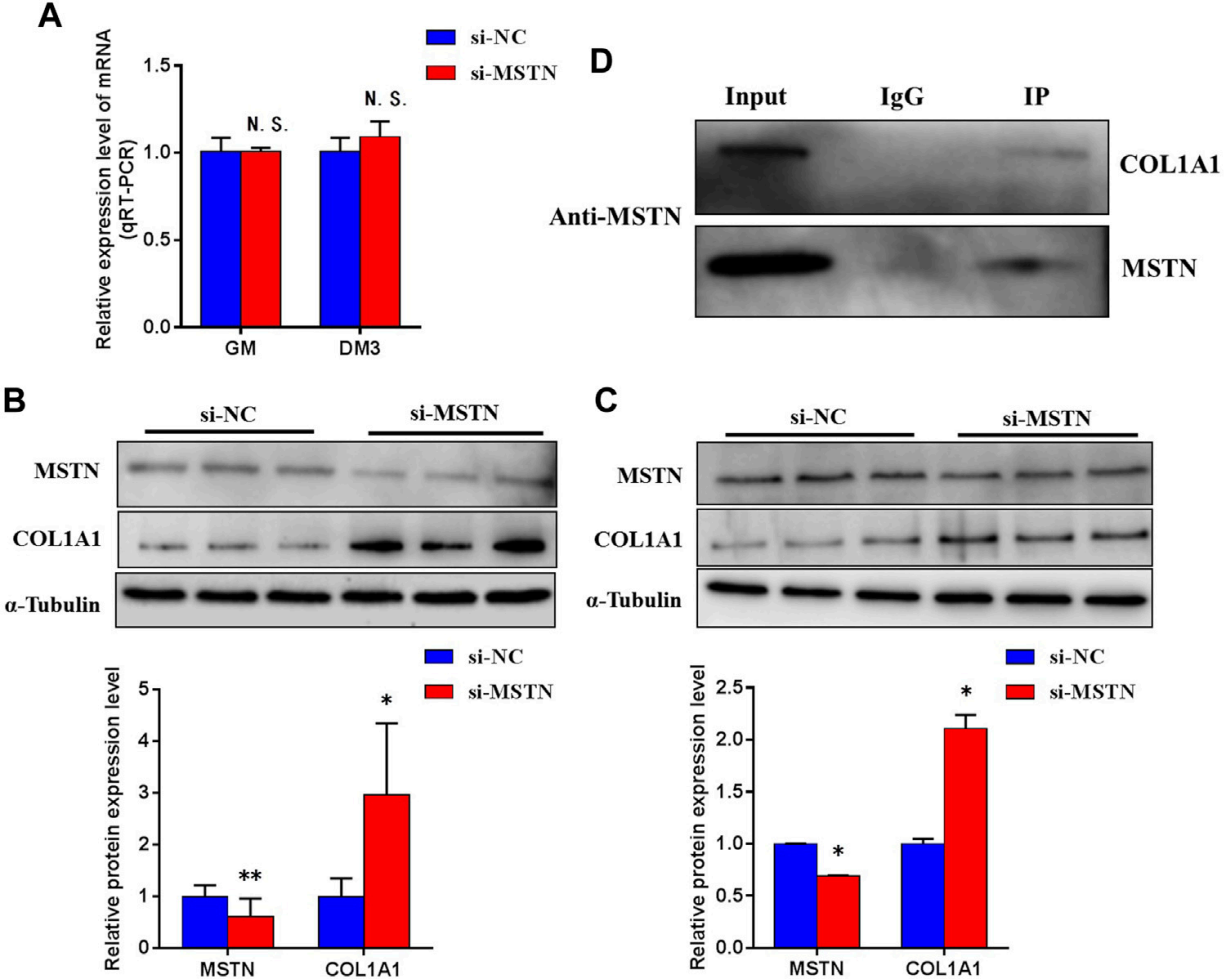
The physical interaction between MSTN and COL1A1. **(A)** Knockdown of MSTN expression in BSMSCs resulted in no significant change in COL1A1 mRNA expression, **(B–C)** but protein expression significantly increased in GM and DM3. **(D)** Co-IP against the endogenous MSTN using anti-MSTN antibody was conducted with the lysate from BSMSCs and the eluent was immunoblotted against anti-COL1A1 antibody. A normal rabbit IgG was used as a negative control for immunoprecipitation. These blots were cropped and original images were shown in Supplementary Figure S9.

To explore the mechanism of MSTN acting on downstream target proteins to regulate Focal adhesion, PI3K-AKT, and Ribosomal pathways. By comparing proteomics analysis, we found that compared with MG.WT, the expression of a large number of extracellular matrix-related proteins in the gluteal muscle tissues of MG.MSTN^+/−^ was up-regulated, among which the expression of COL1A1 The levels were raised by 2.3 times (see Supplementary Table S3B). At the same time, in GM and DM3 BSMSCs transfected with si-MSTN, the mRNA expression level of the COL1A1 gene did not change significantly (Figure 8A), but the protein expression level was significantly increased (Figures 8B,C). To investigate the interaction between MSTN and COL1A1, wild-type GM BSMSCs were collected. CO-IP was performed against MSTN using an anti-MSTN antibody. COL1A1 protein was detected in the eluent of the immunocomplex by western blot using an anti-COL1A1 antibody (Figure 8D), indicating that MSTN interacted with COL1A1.

### Knockdown of COL1A1 Expression Inhibits Proliferation of Bovine Skeletal Muscle Satellite Cells

**FIGURE 9:**
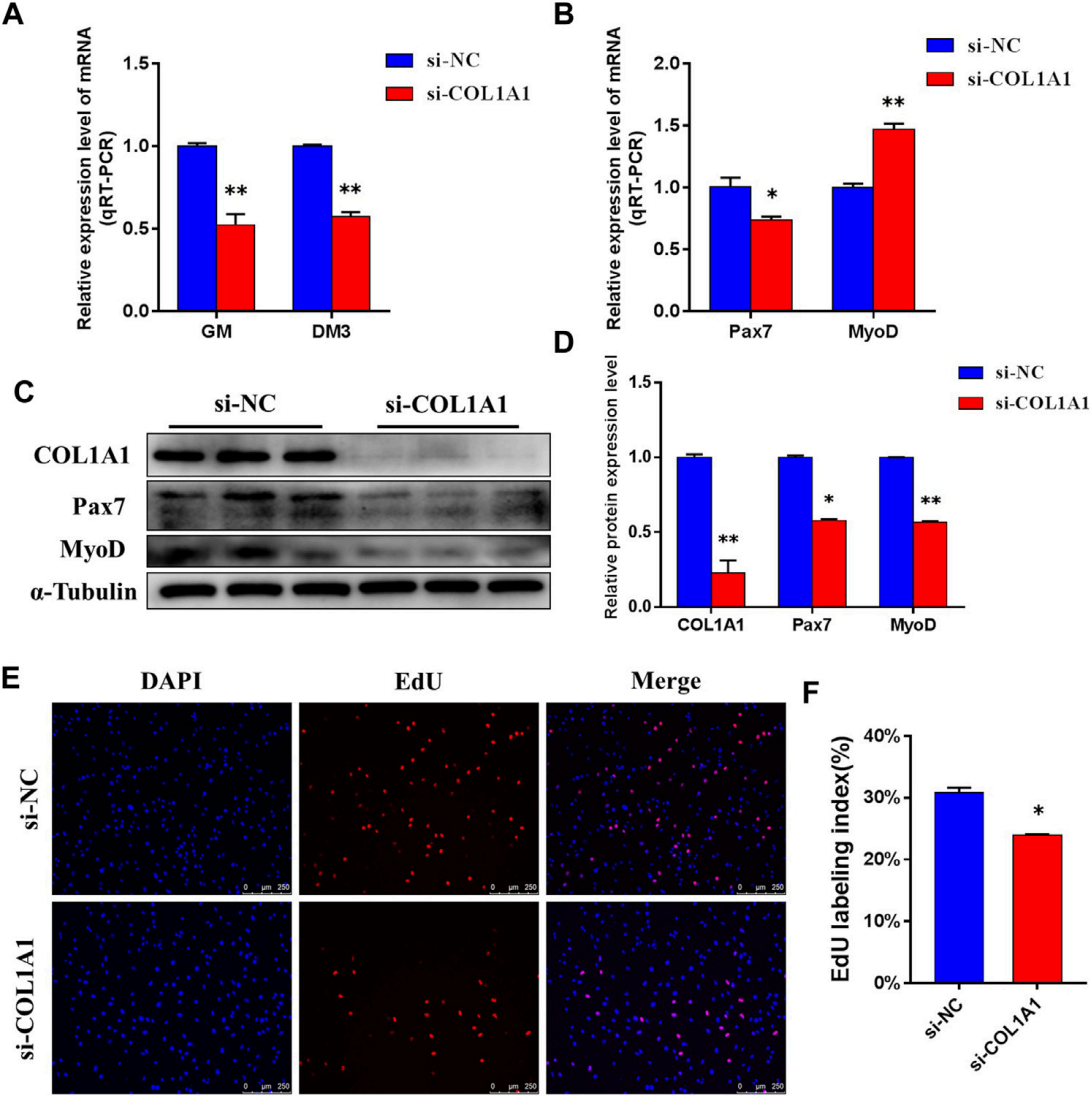
COL1A1 maintains the proliferation of BSMSCs. **(A)** Knockdown COL1A1 significantly decreased COL1A1 expression level in GM and DM3, **(B)** resulting in a significant decrease in the expression level of mRNA in Pax7 and a significant increase in the expression level of mRNA in MyoD, **(C–D)** but the protein expression levels of Pax7 and MyoD were significantly decreased. **(E–F)** EdU assay was performed at 24 h after transfection. The number of EdU-positive cells (E, ×200; scale bars 250 μm) and EdU labeling index (F) were decreased with COL1A1 knockdown. These blots were cropped and original images were shown in Supplementary Figure S10.

After confirming the interaction between MSTN and COL1A1, we tested the effect of knocking down COL1A1 expression on the proliferation of BSMSCs. Designed and synthesized siRNA (si-COL1A1) for COL1A1 knockdown, which resulted in a very significant decrease in the mRNA expression level and protein expression level of COL1A1 in GM BSMSCs (Figure 9A). The expression levels of Pax7 and MyoD, which are related to proliferation, were detected. The results showed that after silencing COL1A1 expression, the mRNA expression level of Pax7 was significantly decreased, the mRNA expression level of MyoD was significantly increased (Figure 9B), and the protein expression levels of Pax7 and MyoD were significantly decreased (Figures 9C,D). The results of the 5-ethynyl-2′-deoxyuridine (EdU) cell proliferation assay showed that when COL1A1 was knocked down, the number of EDU positive cells (Figure 9E) and EDU labeling index (Figure 9F) were all down-regulated. In summary, our experiments show that COL1A1 is essential for maintaining the proliferation of BSMSCs.

### Knockdown of COL1A1 Expression Inhibits Focal Adhesion, PI3K-AKT, and Ribosomal Pathways

**FIGURE 10:**
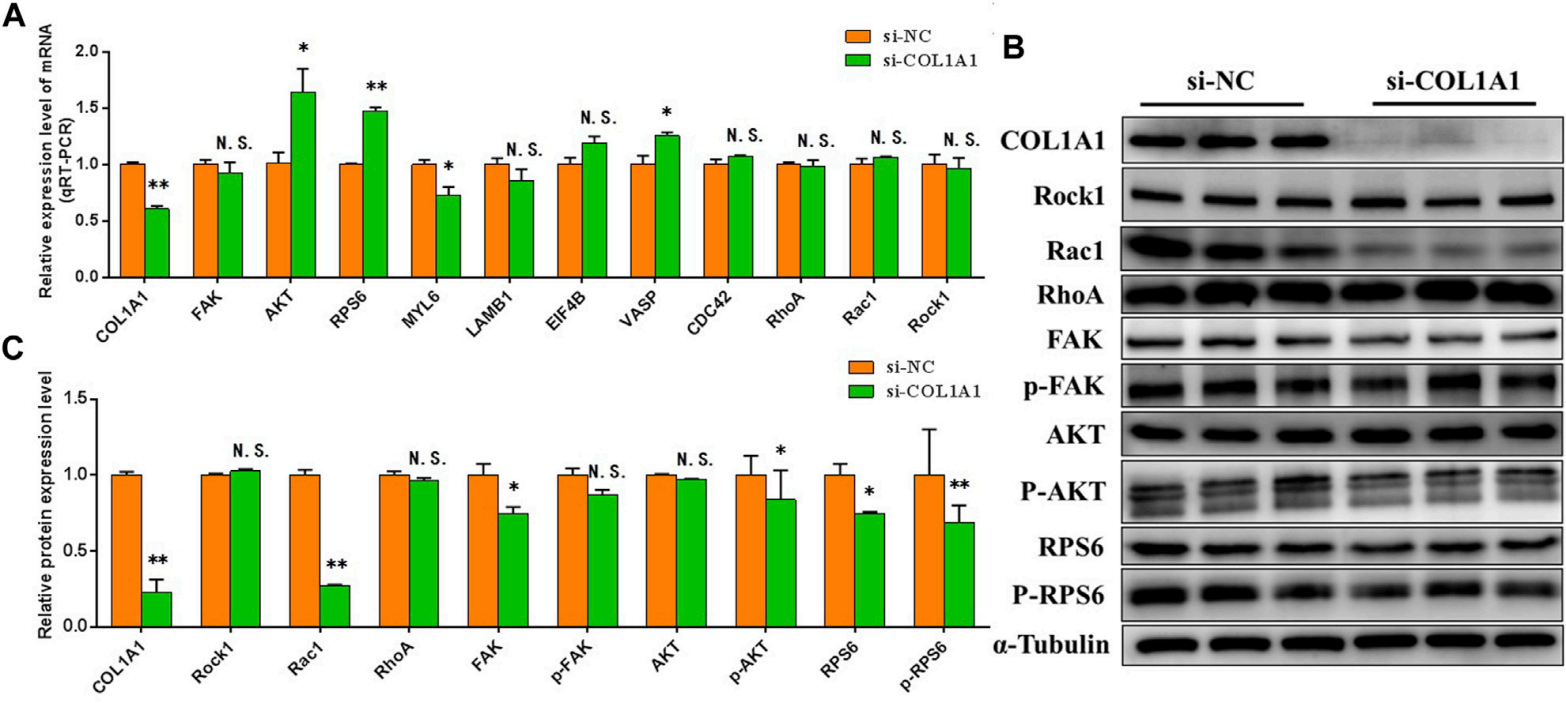
COL1A1 functions in the proliferation of BSMSCs. **(A)** knockdown of COL1A1 expression resulted in focal adhesion, PI3K-AKT and ribosomal pathway-related differentially expressed protein mRNA expression level did not change significantly, **(B–C)** but the protein expression level decreased significantly. These blots were cropped and original images were shown in Supplementary Figure S11.

The effects of knocking down COL1A1 on Focal adhesion, PI3K-AKT, and Ribosomal pathway were detected. The results showed that in BSMSCs transfected with si-COL1A1, the mRNA expression levels of majority differentially expressed proteins involved in focal adhesion, PI3K-AKT, and ribosomal pathways did not change significantly (Figure 10A), but the protein expression levels decreased significantly, especially the protein expression levels of key genes p-FAK, p-AKT1 and p-RPS6 in the pathway (Figures 10B,C). It shows that knockdown of COL1A1 expression inhibits Focal adhesion, PI3K-AKT, and Ribosomal pathways.

## Discussion

Our group has a long-standing research interest in understanding the molecular mechanisms of MSTN knockout in transgenic cattle. The main purpose of these studies is to improve livestock breeds to obtain the best economic benefits. We demonstrated that MSTN knockout promoted skeletal muscle growth in Luxi-MSTN^−/−^ cattle, which showed a higher percentage of lean meat (Yang et al., 2018). It was found that MSTN knockout promoted fatty acid β oxidation and glycolysis in MSTN^−/-^ Inner Mongolia black beef cattle (Xin et al., 2020). In order to further explore the global regulatory mechanism of MSTN related to skeletal muscle growth and development, in the present study, the global proteomics of gluteus muscle of MG. MSTN^+/−^ and MG. WT were analyzed. We believe that the differentially expressed proteins identified in MG.MSTN^+/−^ vs MG.WT group data are caused by MSTN gene knockdown. Through bioinformatics analysis, it was found that the expression abundance of extracellular matrix-related proteins and ribosome-related proteins in the gluteal muscle of MG.MSTN^+/−^ was higher than that of MG.WT (Supplementary Table S3B). Extracellular matrix-related proteins and ribosome-related proteins are important components of focal adhesion, PI3K-AKT, and ribosomal pathways. Therefore, we speculate that MSTN may regulate focal adhesion, PI3K-AKT, and ribosomal pathway.

Extracellular matrix (ECM) is a non-cellular three-dimensional macromolecular network composed of collagens, elastin, fibronectin (FN), laminins, glycoproteins, proteoglycans (PGs), glycosaminoglycans (GAGs), and several other glycoproteins (Theocharis et al., 2016; Birch, 2018; Karamanos et al., 2018; Muncie and Weaver, 2018). The ECM not only provides physical scaffolds embedded by cells but also regulates many cellular processes, including survival, migration, growth, differentiation, homeostasis, and morphogenesis (Gumbiner, 1996; Frantz et al., 2010; Clause and Barker, 2013). The ECM is an important source of growth factors, providing the attachment and controlled release of many growth factors and signal molecules, thereby aiding cell-to-cell signaling (Birch, 2018). Transcriptome studies showed that in the rat model of overload, the expression abundance of the MSTN gene decreased, a large number of extracellular matrix-related proteins increased, and skeletal muscle increased (Mendias et al., 2017; Stantzou et al., 2021). Biological scaffolds composed of ECM have been shown to facilitate the functional reconstruction of several tissue types including the esophagus (Badylak et al., 2005; Desai et al., 2006), heart and vascular structures (Badylak et al., 2006; Quarti et al., 2011), lower urinary tract (Reddy et al., 2000; Boruch et al., 2010), and musculoskeletal tissues (Smith et al., 2004; Franklin et al., 2008; Turner et al., 2010; Valentin et al., 2010; Zhao and Bass, 2018), among others. Mutations in COL1A1 cause osteogenic insufficiency (OI), resulting in decreased muscle mass and function (Veilleux et al., 2014; Veilleux et al., 2015). The extracellular matrix is an important part of focal adhesions and the PI3K-AKT pathway. Focal adhesions are structural links between the extracellular matrix and actin cytoskeleton, which play critical roles in normal physiological events such as cellular adhesion, movement, cytoskeletal structure, and intracellular signaling pathways (Yam et al., 2009). Tyrosine 397 phosphorylation of FAK, a key gene in the adhesion pathway, is essential for normal myoblast differentiation (de Oliveira et al., 2009) and skeletal muscle hypertrophy (Gordon et al., 2001) after hindlimb suspension in rodents, and leads to the binding of FAK to the SH2 domain of the 85 kDa subunit of PI3K, which may lead to the increase of PI3K activity (Appeddu, 1996). The PI3K/AKT signaling pathway is an essential node in mammalian cells that controls cell growth, migration, proliferation, and metabolism (Pompura and Dominguez-Villar, 2018). Previous studies have shown that knockout of the expression of MSTN activates the PI3K-AKT pathway, which can promote muscle development in pigs (Li et al., 2020), which is consistent with the results of this study.

Muscle hypertrophy occurs when the rate of protein synthesis exceeds the rate of degradation. The main factor determining the rate of protein synthesis is ribosome abundance or translational capacity (Wen et al., 2016). In the last couple of decades, studies have revealed that the ribosome has an essential role in the regulation of cell proliferation and growth (Volarevic, 2000; Kirn-Safran et al., 2007) and homeostasis in mammalian organisms. For example, deletions and mutations in genes linked to ribosome biogenesis result in pathologies known as ribosomopathies, which are associated with malformation and growth retardation (Teng et al., 2013). A known role of ribosomal protein RPS6 phosphorylation is the regulation of ribosomal protein synthesis (Jefferies et al., 1994).

The expression levels of MYL6 in muscle tissue were detected by western blot to verify the accuracy of proteomics data. Our Western blotting with anti-phospho-FAK (Tyr-576), Akt (Ser-473), RPS6(Ser-235/236) confirmed the dramatically increased expression of pFAK, pAkt, pRPS6 in MG.MSTN^+/−^ (Figure 3), thus activating the focal adhesion, PI3K-AKT, and ribosomal signaling pathways. Satellite cells are tissue-specific stem cells responsible for skeletal muscle growth and regeneration (Kanisicak et al., 2009). We constructed an MSTN siRNA model by using BSMSCs and found that knockdown of the expression of MSTN activated focal adhesion, PI3K-AKT, and ribosomal signal pathways, and promoted cell proliferation and myogenic differentiation. The interaction between MSTN and COL1A1 is verified by CO-IP technology. At the same time, the COL1A1 siRNA model was constructed, and it was found that knockdown of the expression of COL1A1 inhibited the adhesion plaque, PI3K-AKT, and ribosomal pathway, and inhibited cell proliferation.

**FIGURE 11:**
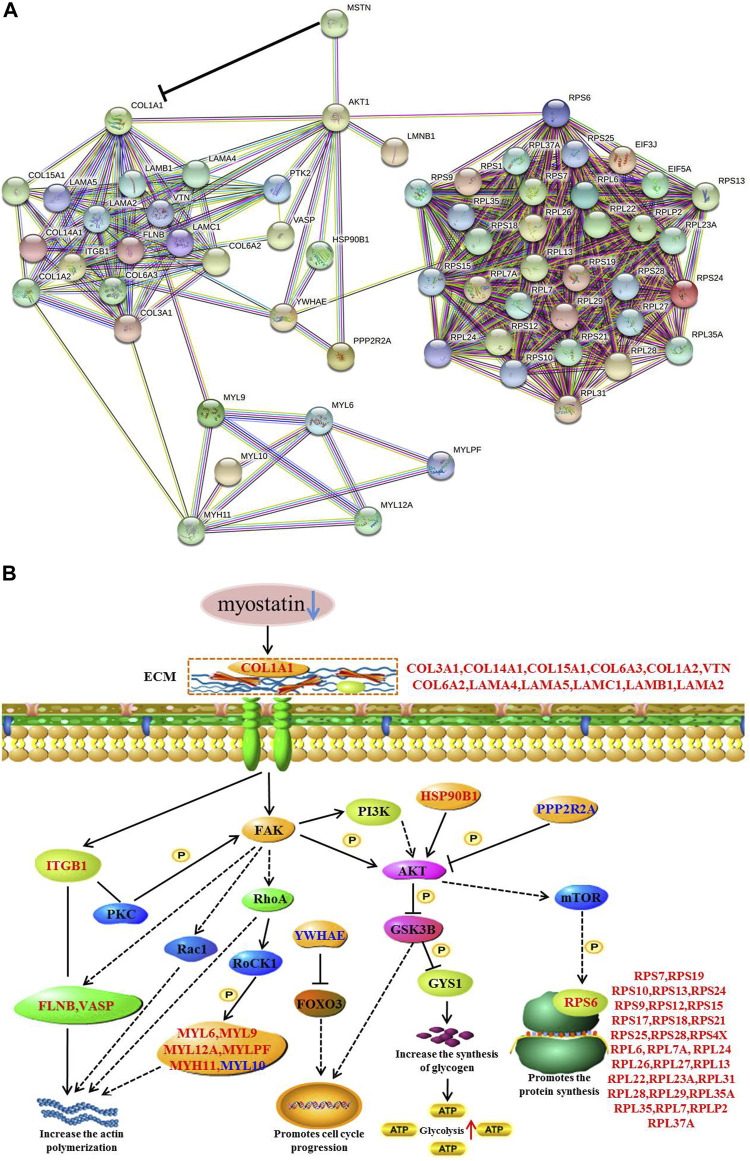
Functional interaction network and a diagrammatic mode illustrate the regulatory mechanism of the MSTN gene on the focal adhesion, PI3K-AKT, and ribosomal pathways. **(A)** MSTN, FAK, AKT1, RPS6, and 55 Differentially expressed proteins were submitted to conduct blast searching against the existing databases in STRING 11 software. **(B)** “→” The activation of the process, “ -| ” the inhibition of the process, “– –” the presence of intermediate steps either unknown or omitted. Up-regulated differentially expressed proteins were marked with red font, while down-regulated differentially expressed proteins were marked with blue font.

Our study using TMT-based quantitative proteomics analyses on skeletal muscle from MG.MSTN^+/−^ and MG.WT, and verified the results of proteomics at the cellular level for the first time. The quantitative proteomics data reveal that MSTN induces abundance changes of extracellular matrix and ribosome-related proteins. Using muscle tissue and cell model verification, we found that MSTN regulates focal adhesion, PI3K-AKT, and ribosomal pathways through its interaction with COL1A1 (Figure 11). By the combination of the results reported in this work and previous studies we conclude that MSTN plays a critical role in muscle development, and knock down the expression of MSTN can activate focal adhesion, PI3K-AKT, and ribosomal pathways, and elevated actin polymerization and protein synthesis.

## Materials and Methods

### Experimental Design and Sample Collection

**FIGURE 12:**
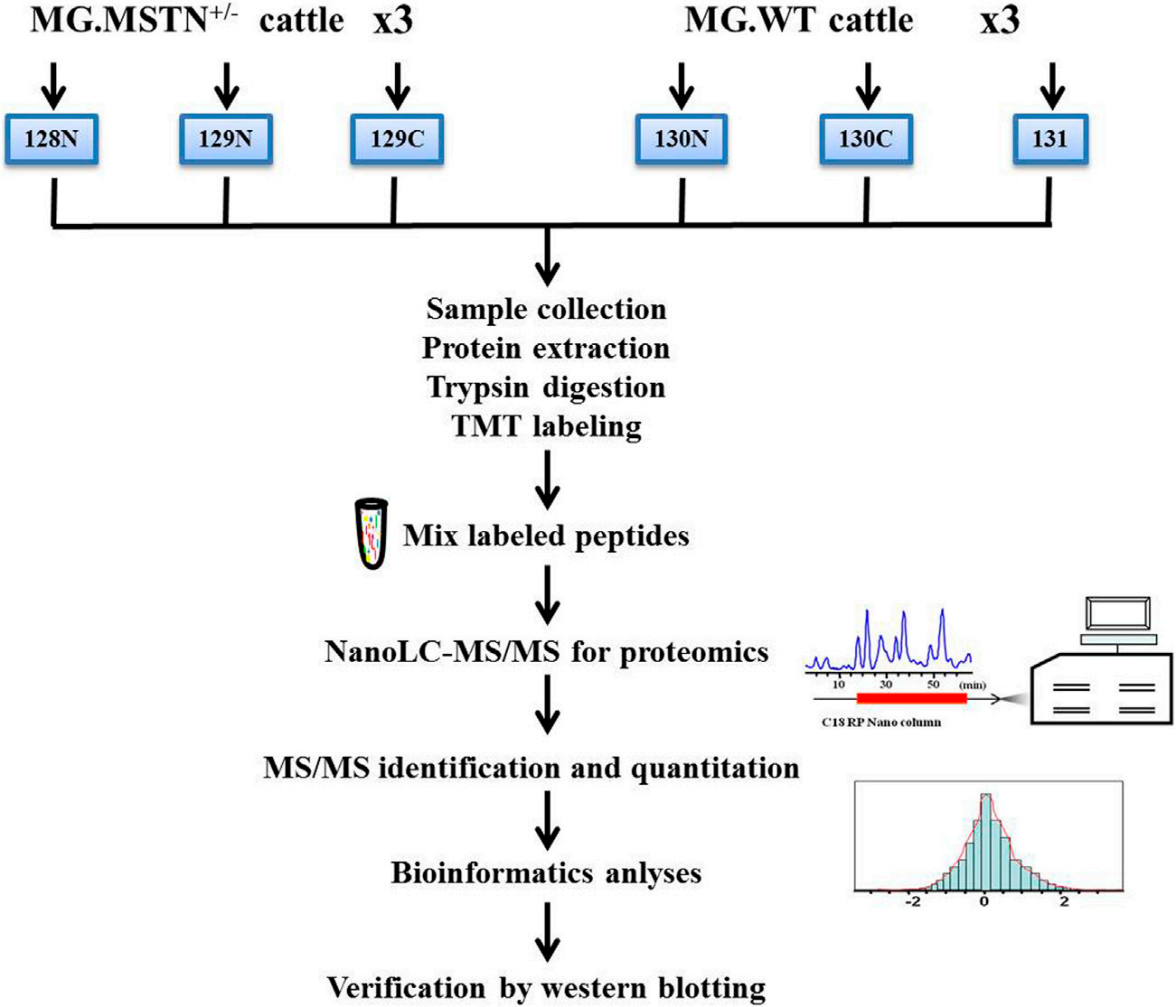
Experimental design and schematic diagram of the proteomics workflow. A total of 3 MSTN^+/−^ Mongolian cattle (MG.MSTN^+/−^) and 3 wild type Mongolian cattle (MG.WT) were analyzed by TMT 6-plex based shotgun-based quantitative proteomics, using the high pH reverse phase label-free liquid chromatography-mass spectrometry (hpRP-nanoLC-MS/MS) workflow, and the discovery results by multiple methods including real-time fluorescence quantitative PCR (qRT-PCR), co-Immunoprecipitation (CO-IP), and western blotting approaches.

In this experiment, 3 MSTN^+/−^ Mongolian cattle and 3 WT Mongolian cattle were selected as the research objects in the animal experimental base of Inner Mongolia University. The experimental cattle were 2-year-old female cattle, healthy and reared in the same environment. The muscle tissue of bovine leg and gluteal muscle was collected by *in vivo* sampling technique, and the individual information was recorded. Quantitative proteomics analysis based on TMT 6-plex were designed and carried out to study the regulation mechanism of MSTN in muscle development. A schematic diagram for experimental design and workflow is shown in Figure 12. All the experiments were conducted in strict accordance with the recommendations in the guidelines for Animal Protection and Utilization of Inner Mongolia University and approved by the Animal Welfare Committee of Inner Mongolia University.

### Protein Extraction and Digestion

Crush gluteal muscle tissue by conventional grinding method, and add RIPA lysis buffer (1% NP-40, 0.5% sodium deoxycholate, 1% SDS solution, 5 ml 1×PBS, 42 ml milli-Q water) containing protease inhibitor and phosphatase inhibitor (RIPA: protease inhibitor: phosphatase inhibitor:100:1:1) to mix well. The homogenized samples were centrifuged at 4°C for 20 min at 15,000 g after incubated for 30 min at room temperature. The supernatant was collected to obtain protein samples, and then the protein concentration was determined by BCA (CWbiotech Ltd., Beijing, China). The proteins were lyophilized and stored at −80°C for further analysis.

The lyophilized proteins were resuspended and denatured for 5 min sonication and 0.5 h of the vortex in 7 M urea, 2 M thiourea with a final concentration of 100 mM phosphate buffer (pH 7.8) containing 0.5 tablets PhosSTOP (phosphatase Inhibitor Cocktail Tablets from Roche) for 5 ml buffer. The protein concentration for each of the 6 samples was quantified by a gel-based analysis as described previously (Chen et al., 2013). Each protein solution was reduced in 10 mM dithiothreitol (DTT) for 1 h at 60°C before being alkylated in 40 mM freshly made iodoacetamide for 10min at room temperature. The reduced and alkylated protein solutions were transferred to 10 K ultrafiltration tubes before being centrifuged at 12,000 g for 20 min. After discarding the liquid in the button of the tube, the supernatant was transferred to a new ultrafiltration tube and subsequently mixed with trypsin (AB Sciex) (1:50 w/w) for overnight protein digestion at 37°C. After digestion, the samples were centrifuged at 12,000 g for 20 min, the sediment (peptides) was dried by vacuum centrifugation.

### Tandem Mass Tag 6-Plex Labeling and High pH Reverse Phase LC Fractionation

The 6-plex tandem mass tag (TMT) labeling was carried out according to Thermo Scientific’s TMT Mass Tagging Kits protocol. Each of the TMT 6-plex label reagents was reconstituted in 45 µL of acetonitrile (ACN), and the digested peptides from each sample were incubated with a specific tag (tags 128N, 129N, 129C used for 3 MG.MSTN^+/−^ samples and tags 130N, 130C, 131 used for 3 MG.WT samples, respectively) for 1 h in room temperature (Figure 12). And then the samples were pooled and dry-out to cation exchange chromatography. The hpRP chromatography was carried out as described previously (Yang et al., 2011). Forty-eight fractions were obtained at 1 min intervals and pooled into 6 fractions based on UV absorbance at 214 nm and with a multiple fraction concatenation strategy (Wang et al., 2011). The trypsin peptides labeled in each component are used for global proteomic analysis of nanoLC-MS/MS analysis.

### Nano-Scale Reverse Phase Chromatography and Tandem MS Analysis

NanoLC-MS/MS analysis was carried out using an Orbitrap Fusion mass spectrometer (Thermo-Fisher Scientific, San Jose, CA) with an UltiMate3000RSLCnano system (Thermo-Dionex, Sunnyvale, CA). 10 μL supernatant were loaded onto a reverse-phase trap column (Thermo Scientific Acclaim PepMap100, 75 μm*2 cm, nanoViper C18) which was connected to a C18 reversed-phase analytical column (Thermo Scientific Easy Column, 10 cm long, 75 μm inner diameter). The samples were loaded at 4 μL/min for 5 min, then run at 400 ml/min from 5 to 30% for 40 min in mobile phase B (98% acetonitrile, 0.1% formic acid), followed by 8 min linear gradient to 80%, then keep at 80% for 8 min in mobile phase B, and finally back to 5% in 2 min. MS data were acquired using a data-dependent top 20 method dynamically choosing the most abundant precursor ions from the survey scan (350–2000 m/z) for Higher Collision Dissociation (HCD) fragmentation. The automatic gain control (AGC) target was set to 3e6. The resolution was set to 70,000 for Survey scans and 17,500 for HCD spectra. Isolation width was 2 m/z. The normalized collision energy was 27 eV and the underfill ratio was 1%.

### Protein Identification and Data Analysis

The NanoLC-MS/MS data were searched against the Bos Taurus database from NCBI using Sequest HT software and converted into MGF files using the Proteome Discoverer 1.4 (PD1.4, Thermo) as previously described (Xin et al., 2017). The parameters of default search for quantitative processing and protein identification were also the same as our previous reports. For the assignment of proteins, at least two distinct peptides should be identified for one protein. The final ratios were subjected to statistical analysis with Perseus software (http://www.coxdocs.org/doku.php), and significance was assessed with t-tests.

### Bioinformatics Analysis

UniProt website (http://www.uniprot.org/) was used to find protein function. DAVID Bioinformatics DAVID Bioinformatics Resources 6.8 (https://david.ncifcrf.gov/) was used to perform the GO annotation and KEGG pathway analysis of differentially expressed proteins. All interactions and construct networks were performed in the STRING 11.0 database (http://string-db.org/).

### Cell Isolation and Culture

Primary BSMSCs were isolated, cultured, and differentiated according to the previous study (Dai et al., 2016). To put this point simply, the muscle tissue of the hindlimb was isolated from 5-6-month-old fetal cattle and digested with type Ⅱ collagenase (Gibco, Waltham, MA) and trypsin (Gibco). The isolated cells were cultured in a growth medium containing 80% Dulbecco’s modified Eagle’s medium (DMEM, Gibco) and 20% fetal bovine serum (FBS, Gibco) until the cell density reached about 70%, the differentiation medium containing 98% DMEM and 2% horse serum (HS, Gibco) was used to induce differentiation.

### SiRNA Synthesis

SiRNA for specific knockdown of MSTN and COL1A1 expression was designed and synthesized by RiboBio (Guangzhou, China). The sequences of si-MSTN and si-COL1A1 are shown in Supplementary Table S1.

### Cell Transfection

According to the manufacturer’s protocol, when the cell density reaches 70–90%, Lipofectamine 3000 (Invitrogen, Carlsbad, CA) is used to transfect siRNA into the original BSMSCs. The final concentrations used for siRNA were 100 nM. After 24 h of transfection, the (GM) of proliferative cells was collected. The differentiation medium was changed and cultured for 72 h, and the cells on the third day of differentiation (DM3) were collected.

### RNA Isolation and Quantitative Real-Time PCR

The total RNA was isolated from BSMSCs using Trizol reagent (Invitrogen). The integrity and concentration of RNA samples were measured by 2.0% agarose gel electrophoresis and NanoDrop 2000c. Then, the PrimeScript II 1st Strand cDNA Synthesis Kit (Takara, Dalian, China) was employed to prepare the first-strand cDNA. Quantitative real-time PCR (qRT-PCR) was performed using All-in-One™ qRT-PCR Mix (Genocopoeia, Guangzhou, China) in a LightCycler^®^ 96 Instrument (Roche, Germany) to detect the expression level of mRNAs. The gene relative expression level was calculated by the 2^−ΔΔCt^ method with TUBB mRNA as an endogenous control of the basal level. All primers used were listed in Supplementary Table S2.

### 5-Ethynyl-2′-deoxyuridine Assay

After 24 h of transfection, BSMSCs were incubated at 37°C for 2 h in 96-well plates with 50 μM EdU (RiboBio, Guangzhou, China). Then, the cells were fixed with 4% paraformaldehyde for 30 min and neutralized with 2 mg/ml glycine solution. The Apollo^®^ staining solution which contains EdU was added and incubated at room temperature for 30 min in the dark to label the DNA in the synthesis stage, and the nucleus was then counterstained with DAPI solution. Three images were randomly obtained by an inverted fluorescence microscope (Leica, Germany) at a magnification of ×200, and the number of EdU positive cells was calculated.

### Co-Immunoprecipitation

Co-immunoprecipitation was conducted using a Pierce™ Crosslink Magnetic IP/Co-IP Kit (Thermo-Fisher Scientific, San Jose, CA). The cells were lysed with a buffer containing 25 mM Tris, 150 mM NaCl, 1 mM EDTA, 1% NP40, 5% glycerol, and a protease inhibitor cocktail. The lysates were immunoprecipitated with either anti-MSTN antibody or anti-IgG antibody, and the eluents were loaded on a 10% SDS–PAGE gel. The blots were incubated overnight at 4°C with either anti-MSTN antibody or anti-COL1A1 antibody. After washing in TBS containing 0.05% Tween 20, then incubated with secondary antibody for 1 h before detection using ECL chemiluminescent substrate (Solarbio).

### Western Blot Analysis

GM phase and DM3 phase cells were collected and lysed with RIPA buffer with protease inhibitor and phosphatase inhibitor, and the total proteins were identified. The total protein concentration of the extract was determined by BCA method. Then, equal amounts of cells lysate were resolved by 10% SDS-PAGE and transferred onto polyvinylidene difluoride membranes (Millipore, Burlington, MA). The membranes were blocked with 5% BSA for 1 h, incubated with primary antibody at 4°C overnight, then incubated with secondary antibody for 1 h prior to detection using ECL chemiluminescent substrate (Solarbio). Several proteins were validated by western blot analysis using antibodies against the following proteins: Pax7 (1:100, DSHB, United States), MyoG (1:100, DSHB, United States), MyoD (1:100, DSHB, United States), MyHC (1:100, DSHB, United States), MSTN (1:100, Santa Cruz, United States), p-AKT1 (Ser-473) (1:200, Santa Cruz, Dallas, United States), COL1A1 (1:5,000, Abcam, United States), FAK (1:1,000, Abcam, United States), Rock1 (1:5,000, Abcam, United States), AKT1 (1:5,000, Abcam, United States), RhoA (1:1,000, NewEast, China), Rac1 (1:1,000, NewEast, China), LAMB1 (1:1,500, Sangon Biotech, China), MYL6 (1:500, Sangon Biotech, China), ACTN4 (1:1,000, Sangon Biotech, China), RPS6 (1:500, Sangon Biotech, China), p-FAK (Tyr-473) (1:500, Sangon Biotech, China), p-RPS6 (Ser-235/236) (1:500, Sangon Biotech, China), and reference gene Alpha-Tubulin (1:5,000, Abcam, United States). The gray values of each protein band were calculated using ImageJ software.

## Data Availability

The datasets presented in this study can be found in online repositories. The names of the repository/repositories and accession number(s) can be found in the article/Supplementary Material.
