# Supplementary material for: Proteomic Studies on the Mechanism of Myostatin Regulating Cattle Skeletal Muscle Development
Source: Front Genet. 2021 Nov 16;12:752129. doi: 10.3389/fgene.2021.752129 (PMC8635237; doi:10.3389/fgene.2021.752129)
Supplement: Supplementary file 1 [file DataSheet2.PDF]

Supplementary Table 1:

siRNAs sequences for MSTN and COL1A1 knockdown

Fragment name	Sequence (5’-3’)
si-bta-MSTN	GCACAAAGACATGTCATGT
si-bta-COL1A1	ACTGGTATATCAGCAAGAA

Supplementary Table 2:

Nucleotide sequences of the primers used for real-time quantitative PCR

Genes	Direction	Primer sequence (5’-3’)
MSTN	F	TGTGGAAAAAGAGGGGCTGT
	R	AGCTGTTTCCAGGCGAAGTT
Pax7	F	AGCCAGAGTTTCAACGGGAG
	R	GTCGCCAACAGACAACACAC
MyoD	F	ACGGCATGATGGACTACAGC
	R	AGGCAGTCGAGGCTCGACA
MyoG	F	GGCTGACAAATGCCAGACTATCC
	R	TGGTCCCTTGCTTTATCTCCCT
MyHC	F	CTGGAATCCGGAGGCAGAA
	R	TTTTCGAAGGTAGGGAGCGG
COL1A1	F	CGGCTACGACTTGAGCTTCCTG
	R	GTCACGGACCACATTGGCATCA
COL6A3	F	TTCCTCTTGGAGCATTGGCA
	R	ATGTGGGTTGCCGTTGAACT
LAMA2	F	AGAAGGCTATGCGTTGGTCAGT
	R	TCAGGTCTCGTGTGGCAAGGTA
Rock1	F	ACCAGGAAGGTGTACGCTATGA
	R	GCTGAACAACCCAAGGACTGT
VASP	F	CCCTGGGAGAAGAACAGCACAA
	R	CGTCACTGGAACTAGGCGTAGC
FLNB	F	CAAGCCTGCCGAGTTCACCATC
	R	CCATCGCCGTTGTCTGAGCATT
FAK	F	TGGCTGCTGCTTACCTTGACC
	R	AGAACGCTCCACACCAGTCC
Rac1	F	TCCCAACACACCCATCATCCT
	R	GGCGTCAGCTTCTTCTCCTTC
RhoA	F	GATGTCCAACCCACCTGACC
	R	AATTAGCGCCTGGTGTGTCA

CDC42	F	ACGACCGCTGAGTTATCCACAA
	R	GGGTCCCAACAAGCAAGAAAGG
ACTN4	F	GGAGCGGTTACCTAAGCCAGAG
	R	GCGATGAAGTCCAGTGCCTTGT
MYL6	F	CTATGAAGCGTTTGTGAGGCA
	R	CCTCAGCCATTCAGCACCAT
MYL9	F	CCTGCTCCCACATATACCGT
	R	CTCAGCCCGTTTCCTTCACT
MYL12A	F	AGCCAAACGTTCCTTGTTGC
	R	AGAGATGCATGTGCCCCAAA
RPS6	F	CGAATCAGTGGCGGGAACGATA
	R	ACTCAGTAGCAGGCGAACTCTG
EIF4B	F	TCGTGATGGGTATCGGGACAGT
	R	AGTCTCTGCCACCTCGGTCAT
HSP90B1	F	GAGTCTCCGTGTGCTCTGGTAG
	R	TGTCCTTGCCTGTCTGGTATGC
PPP2CA	F	GGGTCCAATGTGTGACTTGC
	R	CCTGCCCAAAGGTGTAACCA
TUBB	F	CAACAGCACAGCCATCCAGGA
	R	TCTCAGCCTCGGTGAACTCCAT

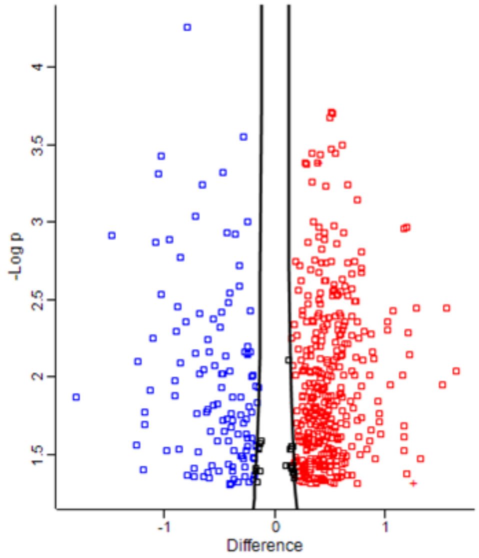

Supplementary Figure 1: Volcanic map of differentially expressed proteins.

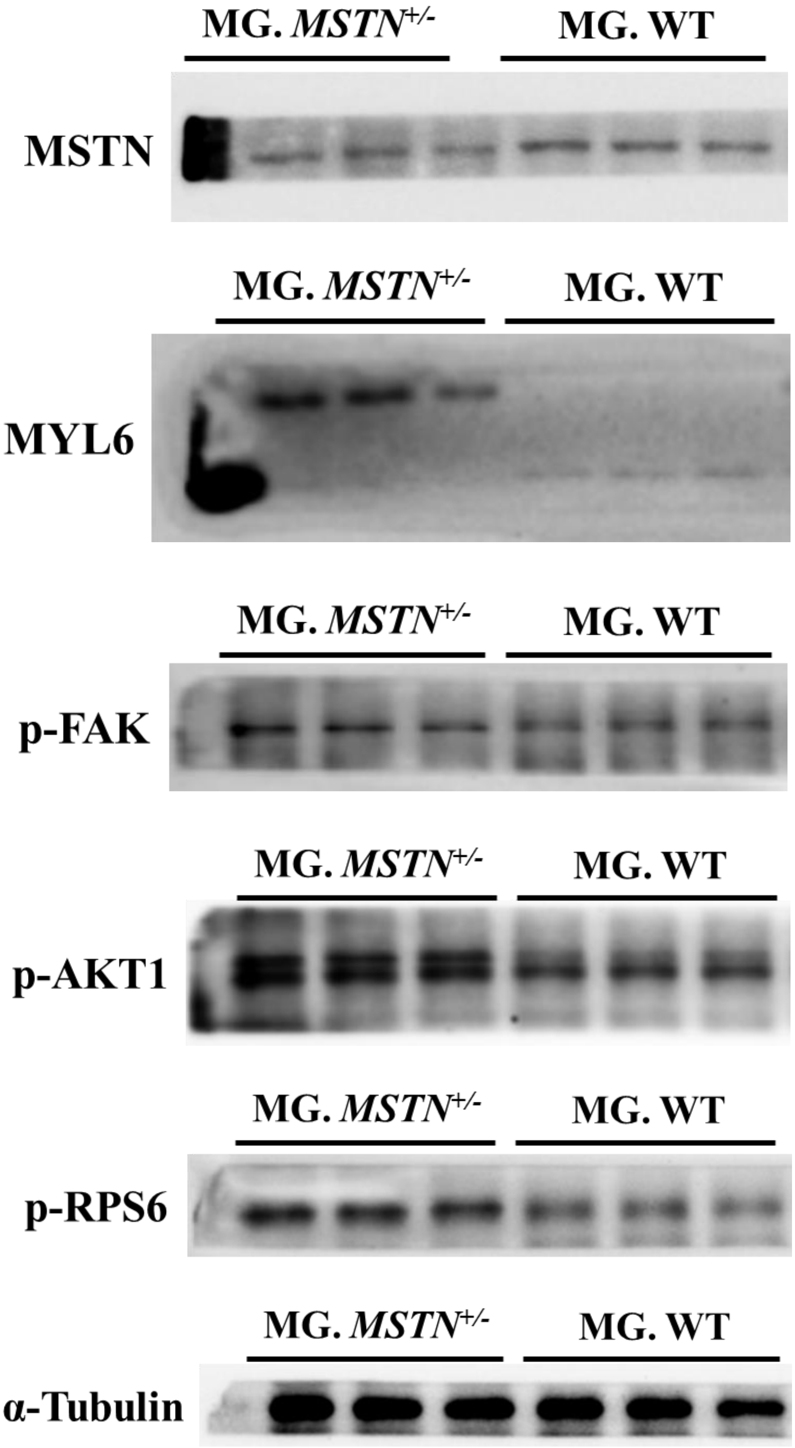

Supplementary Figure 2: Western blot result of MSTN, MYL6, pFAK(Tyr-473), pAKT1(Ser-473) and pRPS6(Ser-235/236) in muscle tissue.

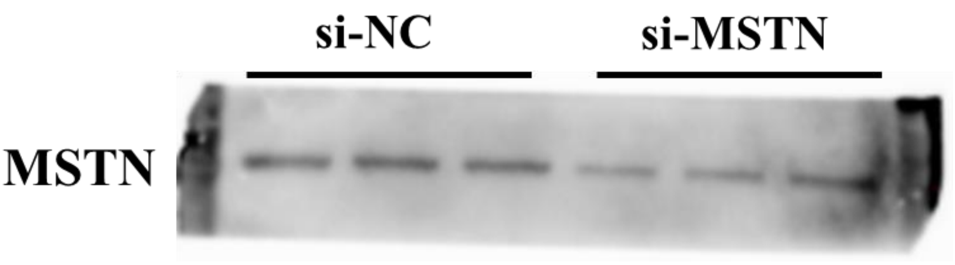

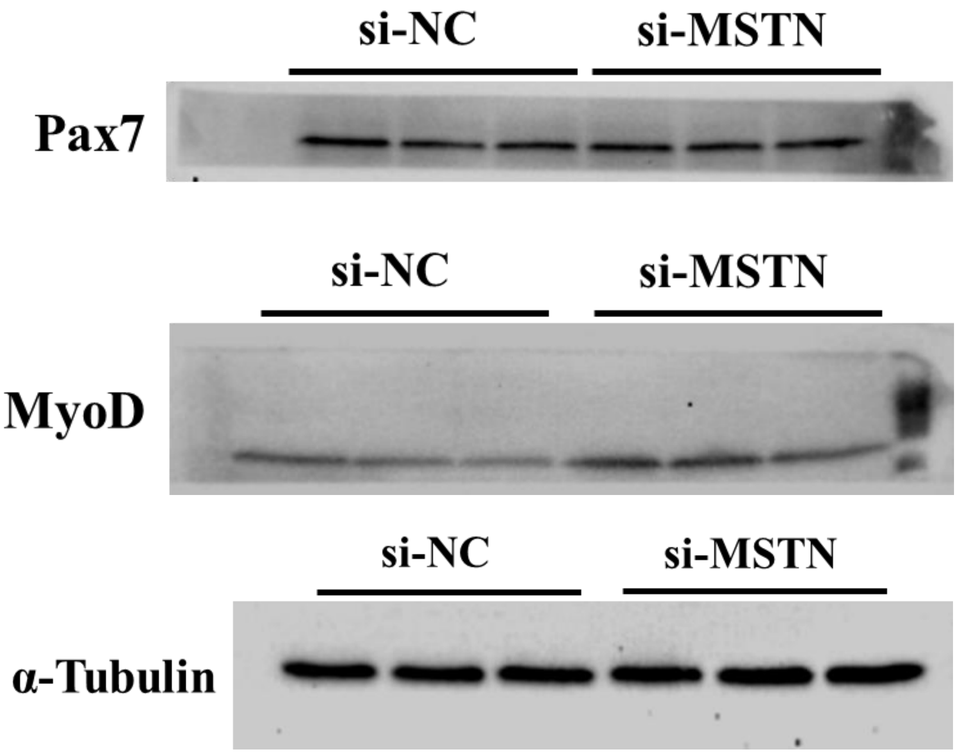

Supplementary Figure 3: Western blot result of MSTN, Pax7 and MyoD proteins in GM bovine skeletal muscle satellite cells.

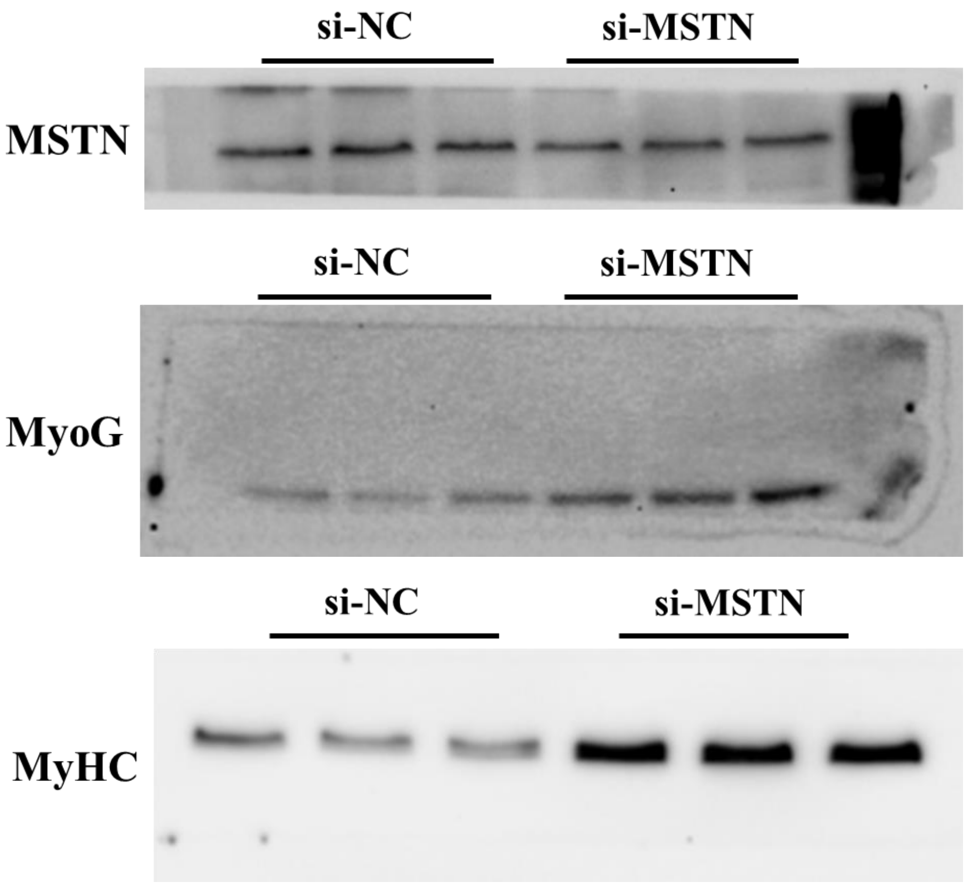

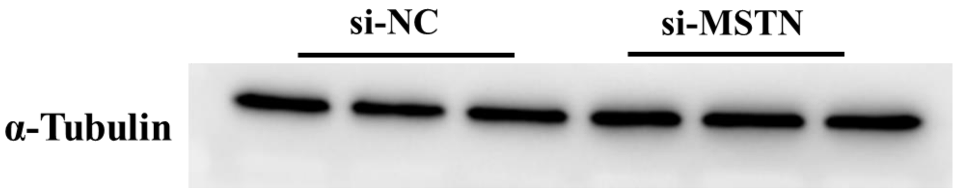

Supplementary Figure 4: Western blot result of MSTN, MyoG and MyHC proteins in DM3 bovine skeletal muscle satellite cells.

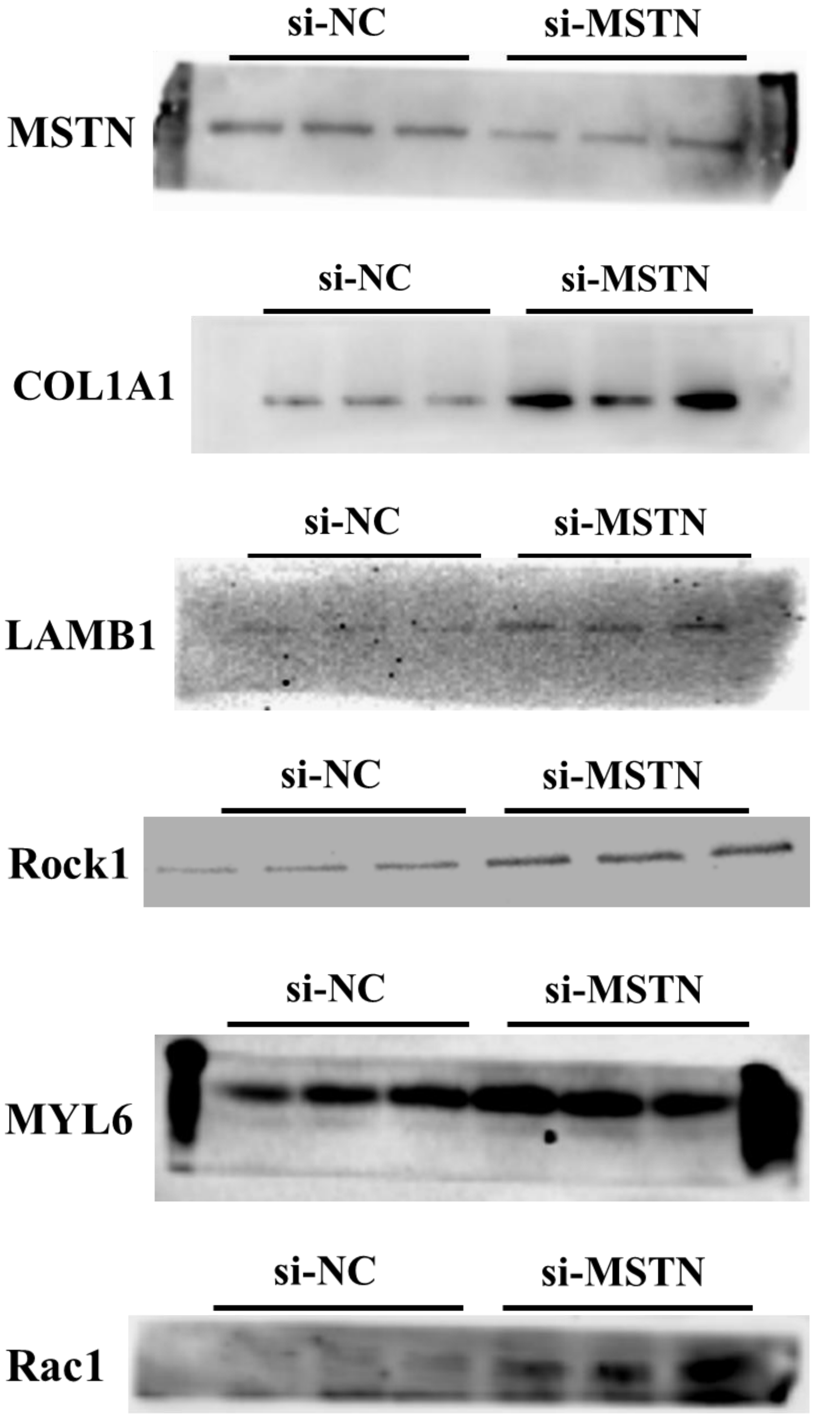

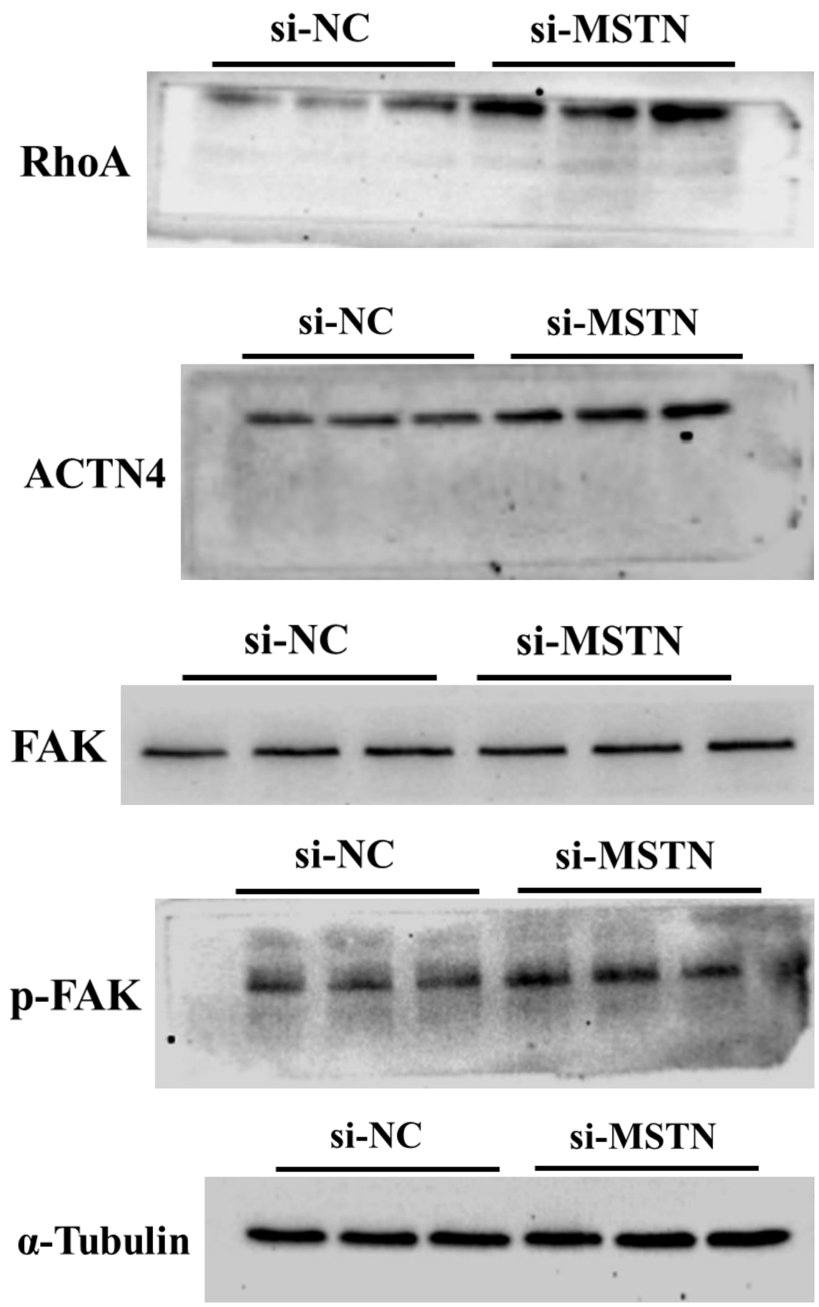

Supplementary Figure 5: Western blot result of MSTN, COL1A1, LAMB1, Rock1, MYL6, Rac1, RhoA, ACTN4, FAK and pFAK (Tyr-473) in GM bovine skeletal muscle satellite cells.

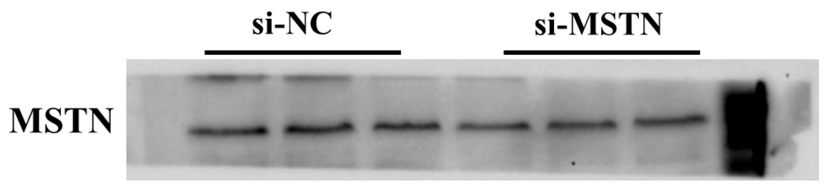

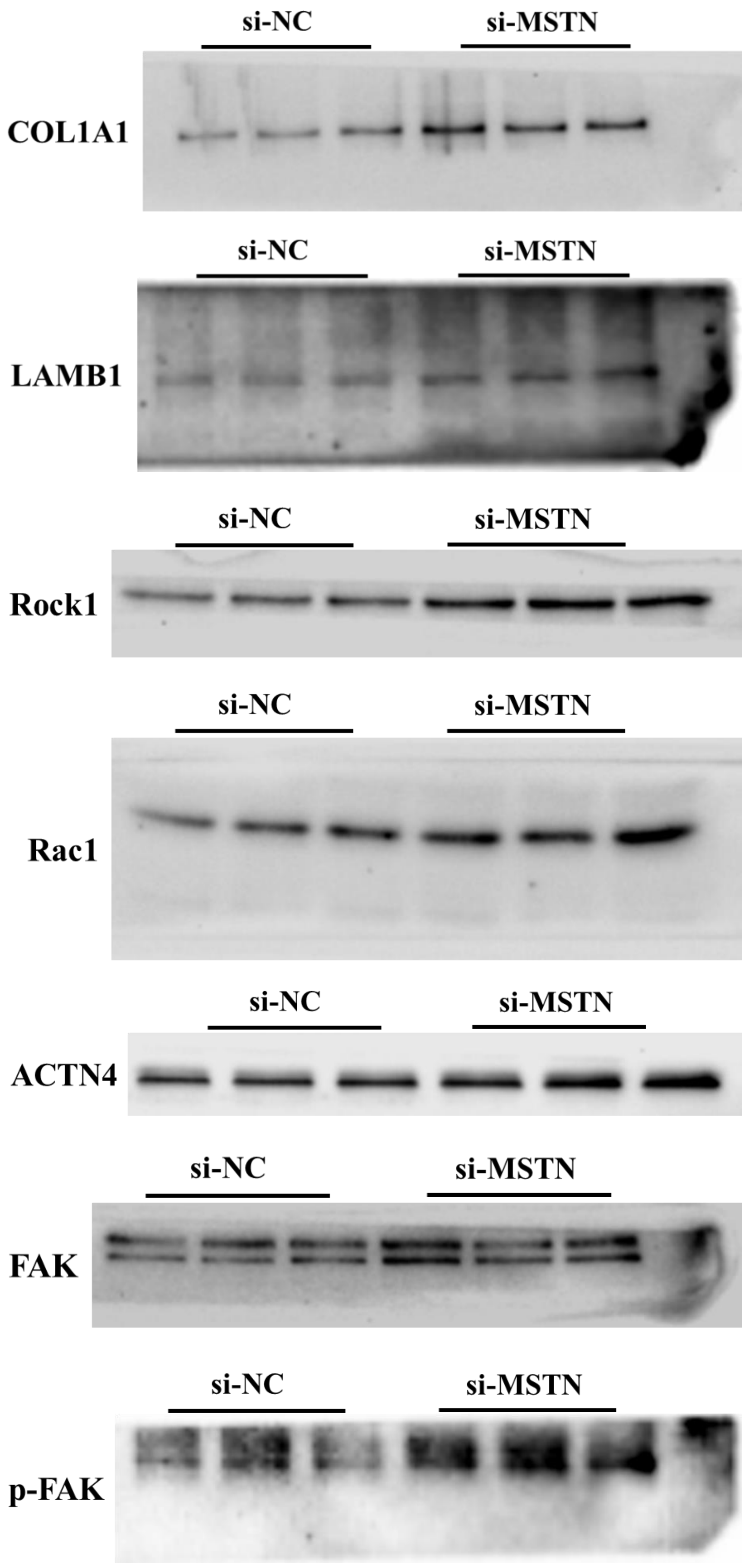
si-NC
si-MSTN
COL1A1
si-NC
si-MSTN
LAMB1
si-NC
si-MSTN
Rock1
si-NC
si-MSTN
Rac1
si-NC
si-MSTN
ACTN4
si-NC
si-MSTN
FAK
si-NC
si-MSTN
p-FAK

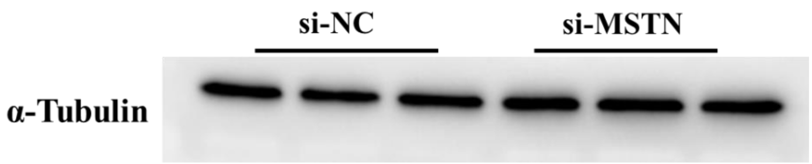

Supplementary Figure 6: Western blot result of MSTN, COL1A1, LAMB1, Rock1,Rac1, ACTN4, FAK and pFAK (Tyr-473) in DM3 bovine skeletal muscle satellite cells.

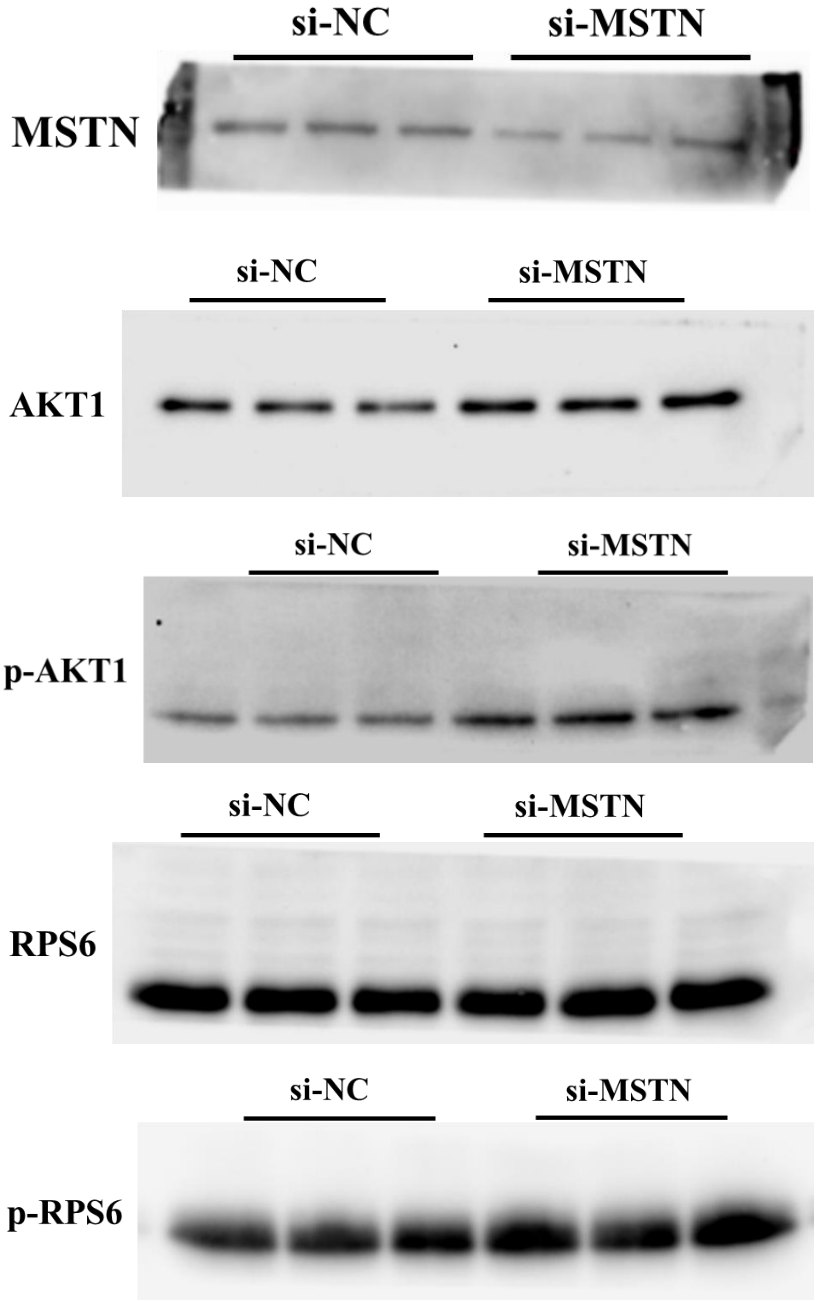

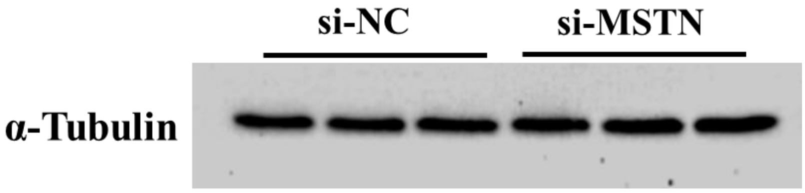

Supplementary Figure 7: Western blot result of MSTN, AKT1, pAKT1 (Ser-473), RPS6 and pRPS6 (Ser-235/236) in GM bovine skeletal muscle satellite cells.

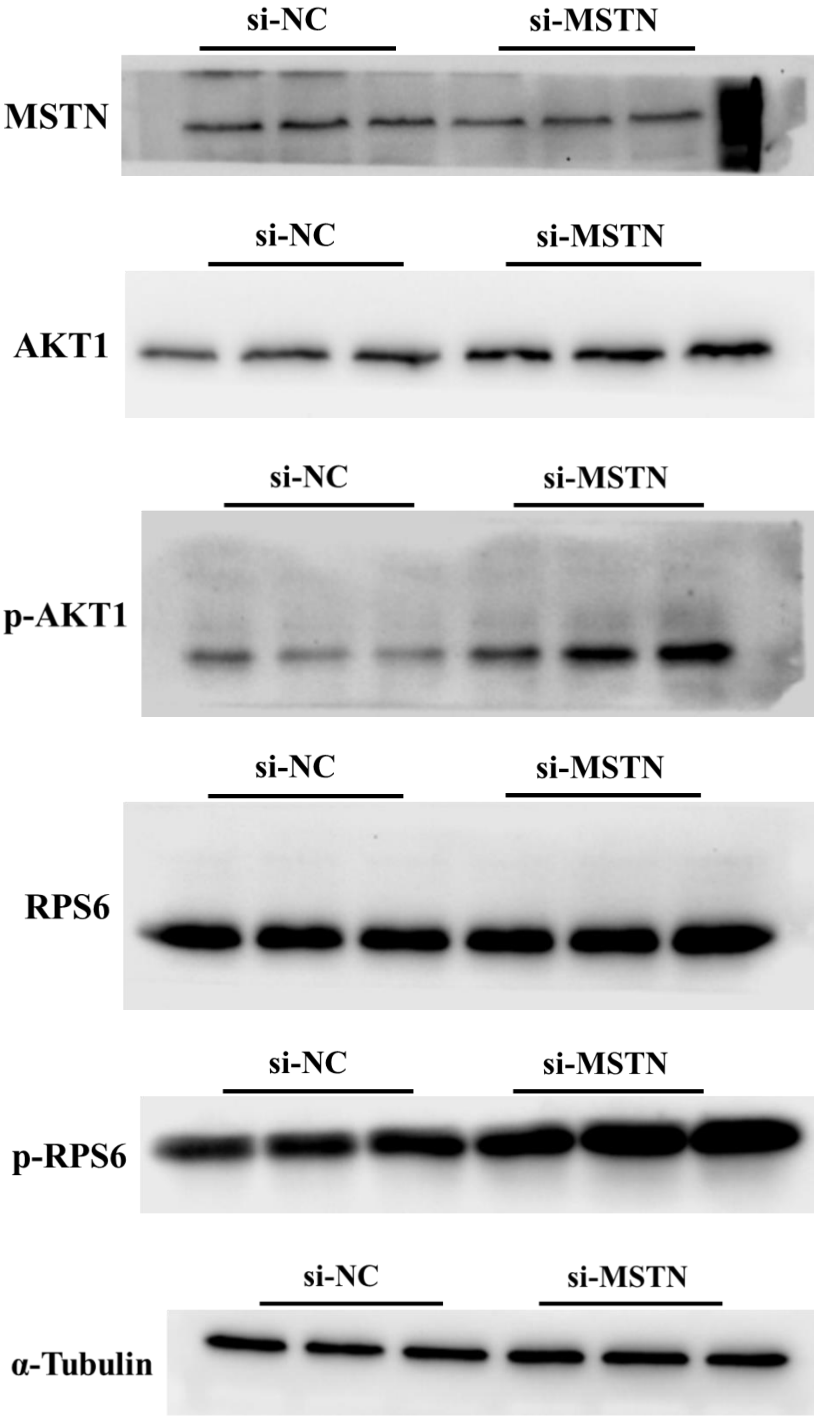

Supplementary Figure 8: Western blot result of MSTN, AKT1, pAKT1 (Ser-473), RPS6 and

pRPS6 (Ser-235/236) in DM3 bovine skeletal muscle satellite cells.

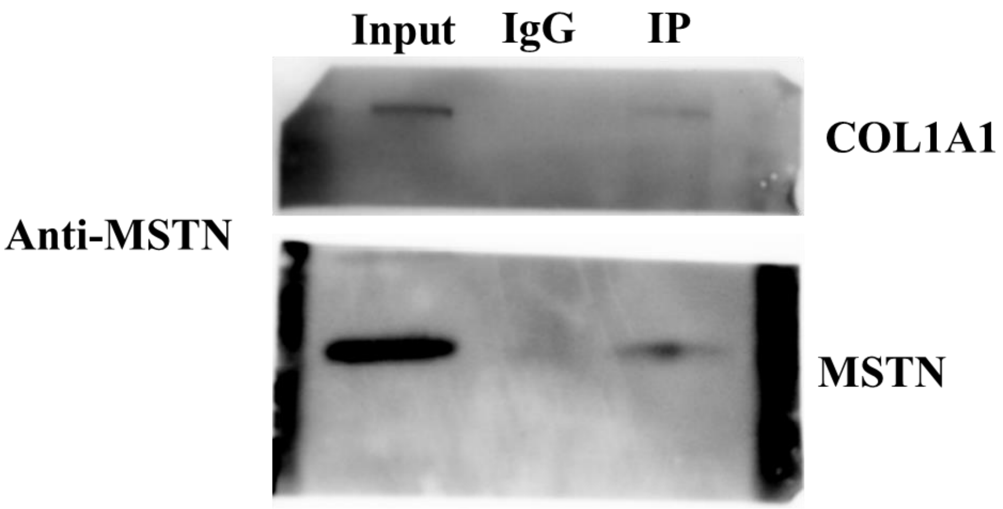

Supplementary Figure 9: CO-IP result of MSTN and COL1A1 proteins.

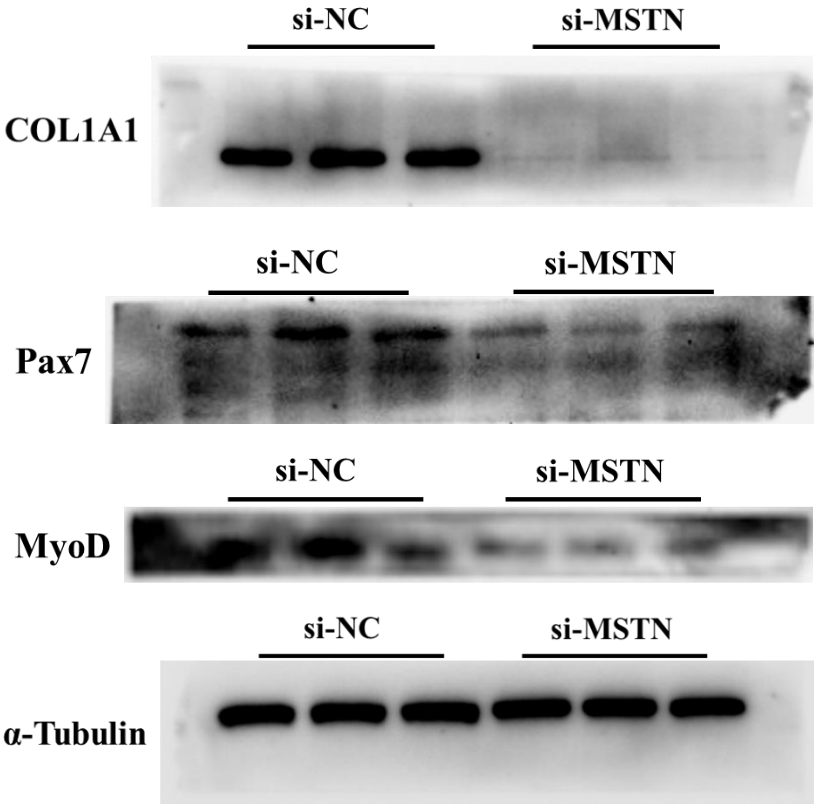

Supplementary Figure 10: Western blot result of COL1A1, Pax7 and MyoD proteins.

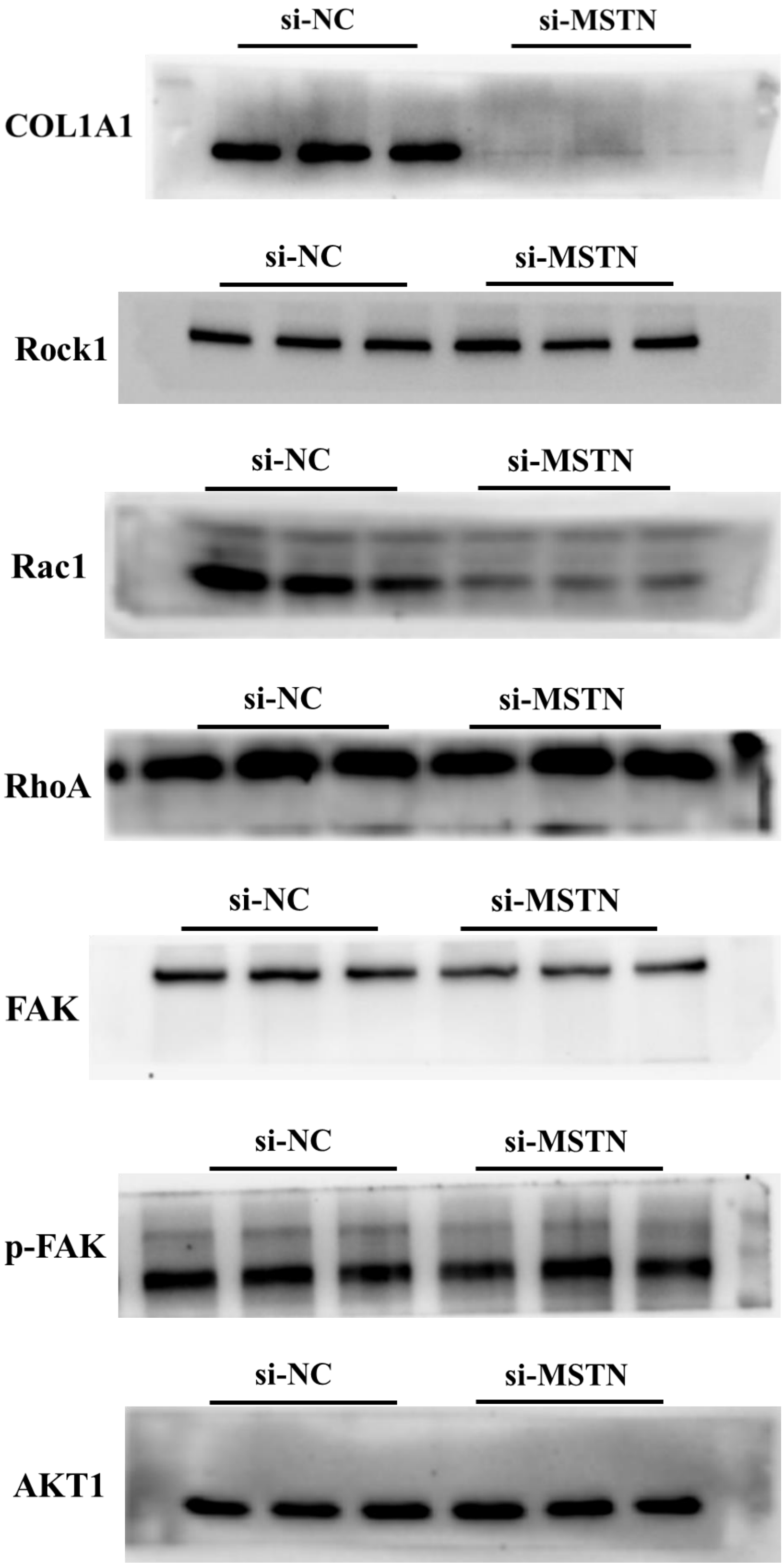
si-NC
si-MSTN
COL1A1
si-NC
si-MSTN
Rock1
si-NC
si-MSTN
Rac1
si-NC
si-MSTN
RhoA
si-NC
si-MSTN
FAK
si-NC
si-MSTN
p-FAK
si-NC
si-MSTN
AKT1

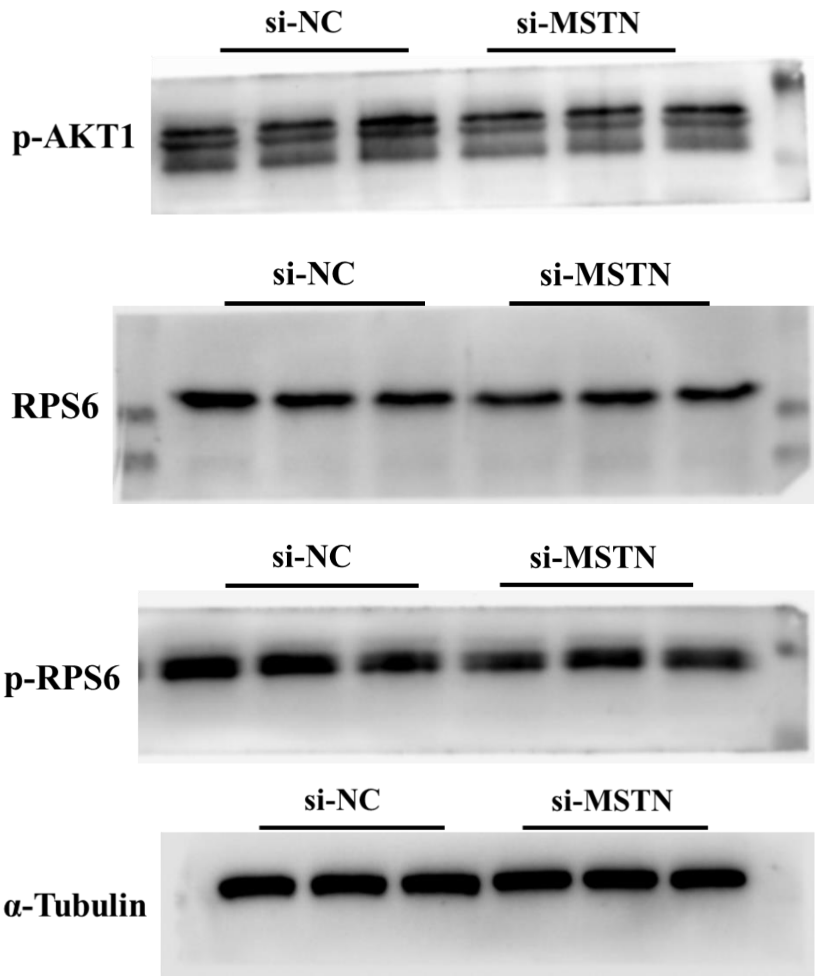

Supplementary Figure 11: Western blot result of COL1A1, Rock1, Rac1, RhoA, FAK, pFAK(Tyr-473), AKT1, pAKT1(Ser-473), RPS6 and pRPS6(Ser-235/236).
