# Supplementary material for: Proteomic Studies on the Mechanism of Myostatin Regulating Cattle Skeletal Muscle Development
Source: Front Genet. 2021 Nov 16;12:752129. doi: 10.3389/fgene.2021.752129 (PMC8635237; doi:10.3389/fgene.2021.752129)
Supplement: Supplementary file 2 [file DataSheet1.PDF]

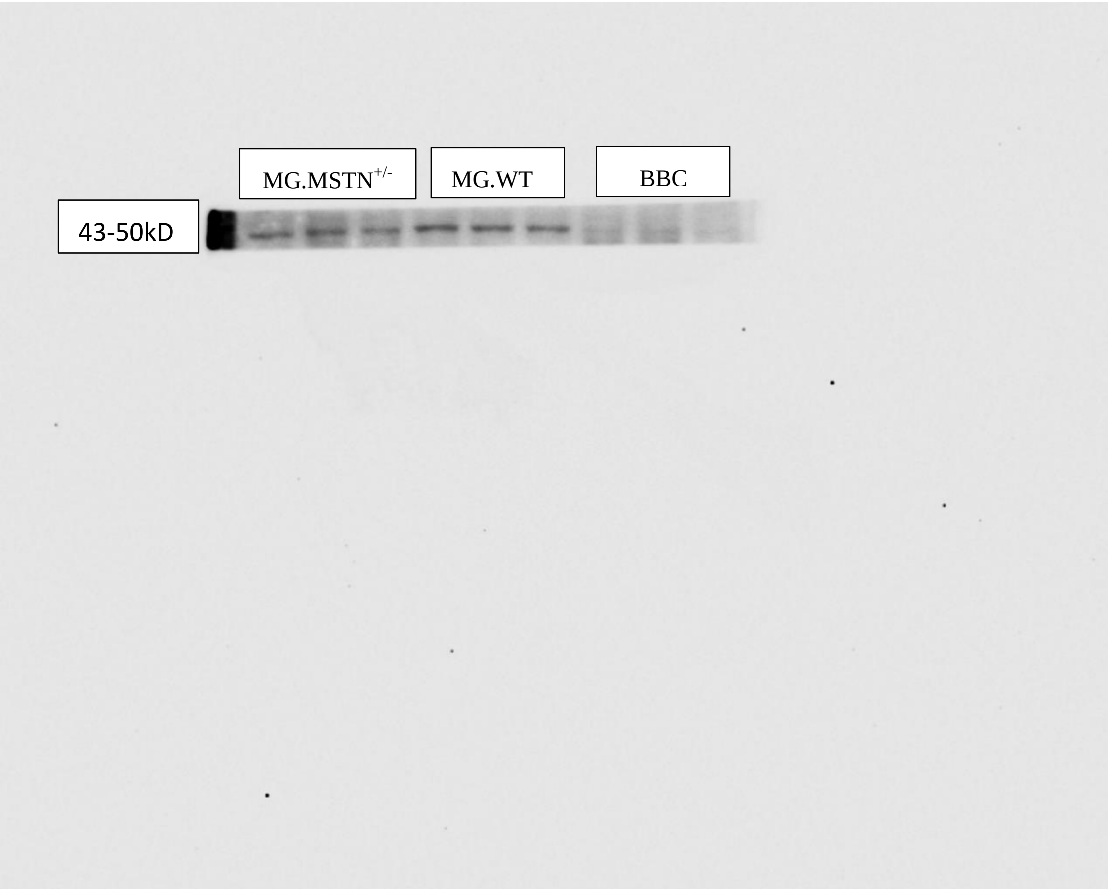

Figure 5, Western blotting results of MSTN

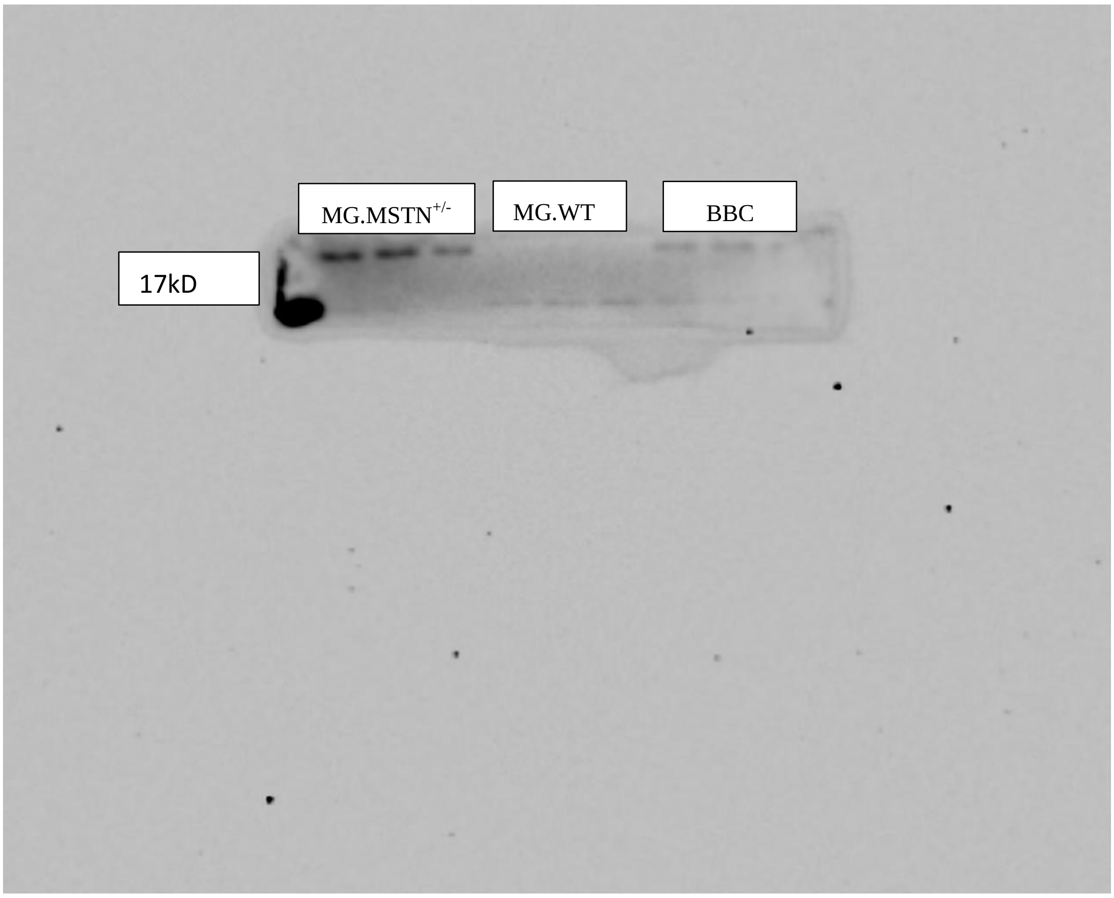

Figure 5, Western blotting results of MYL6

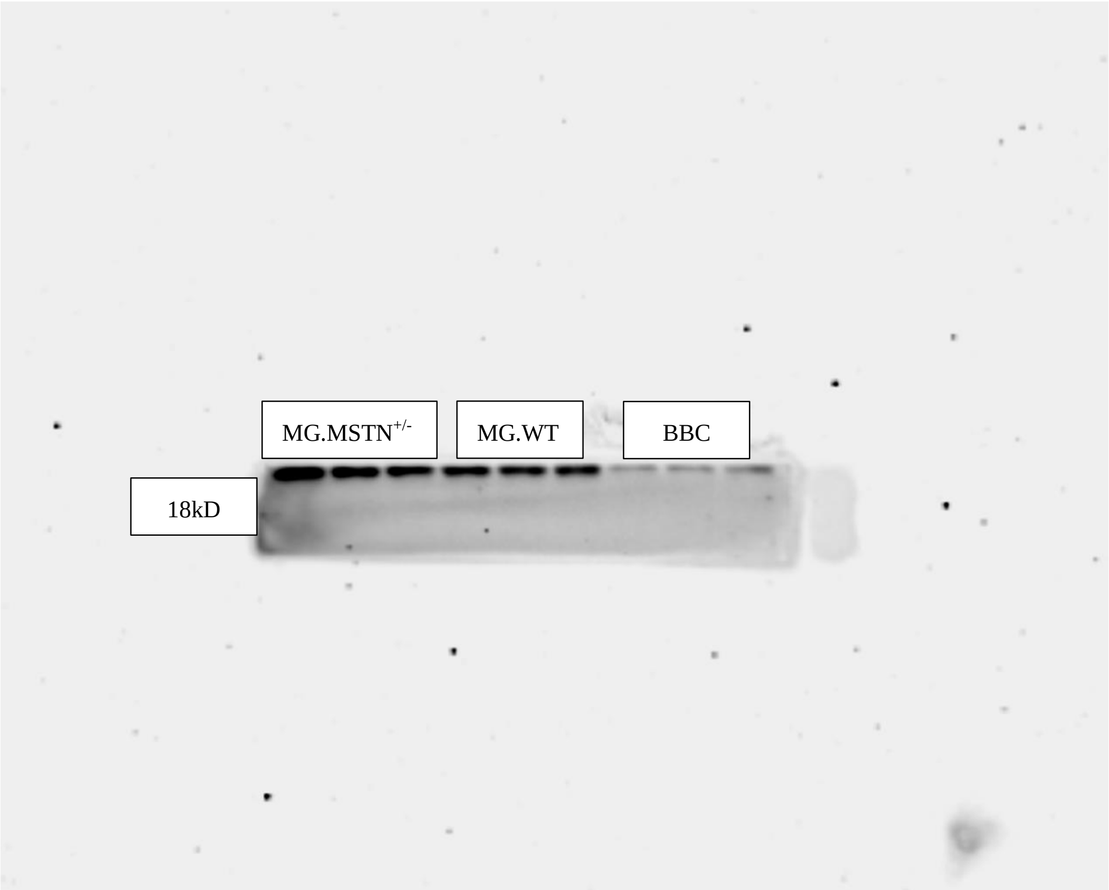

Figure 5, Western blotting results of NDUFA6

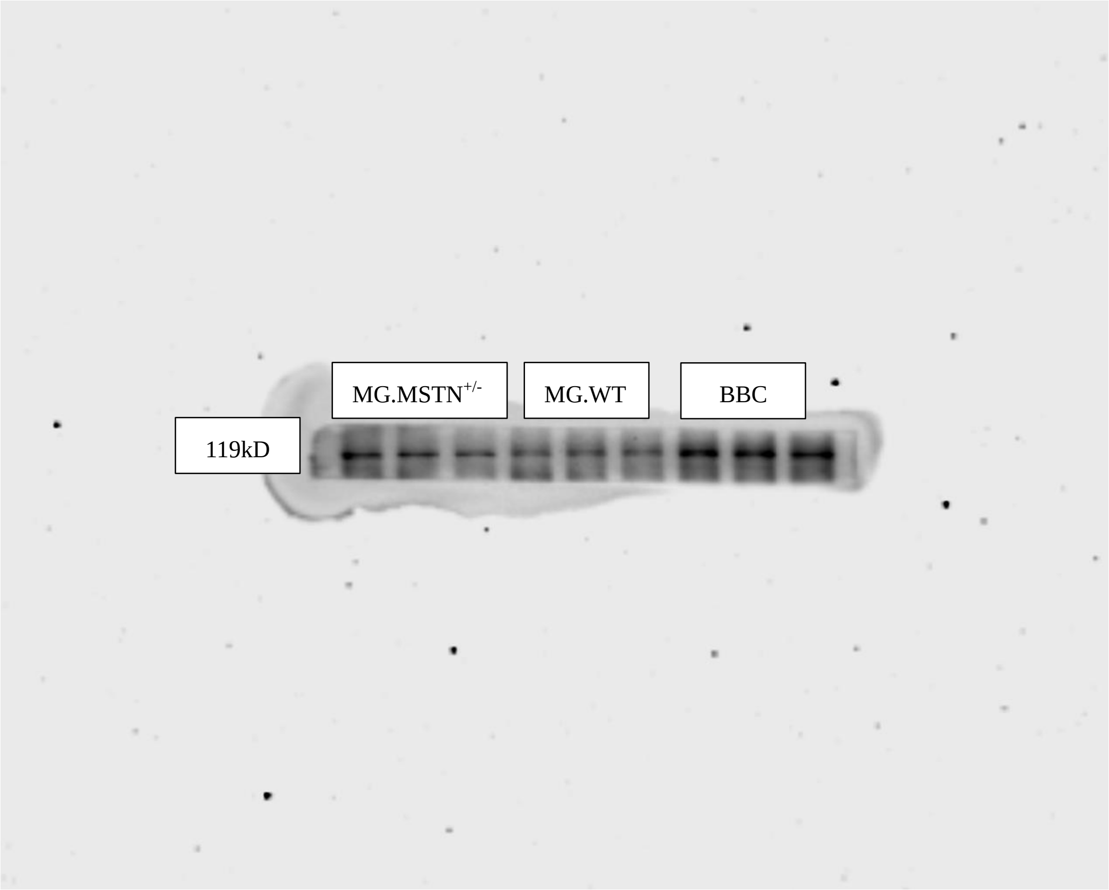

Figure 5, Western blotting results of p-FAK

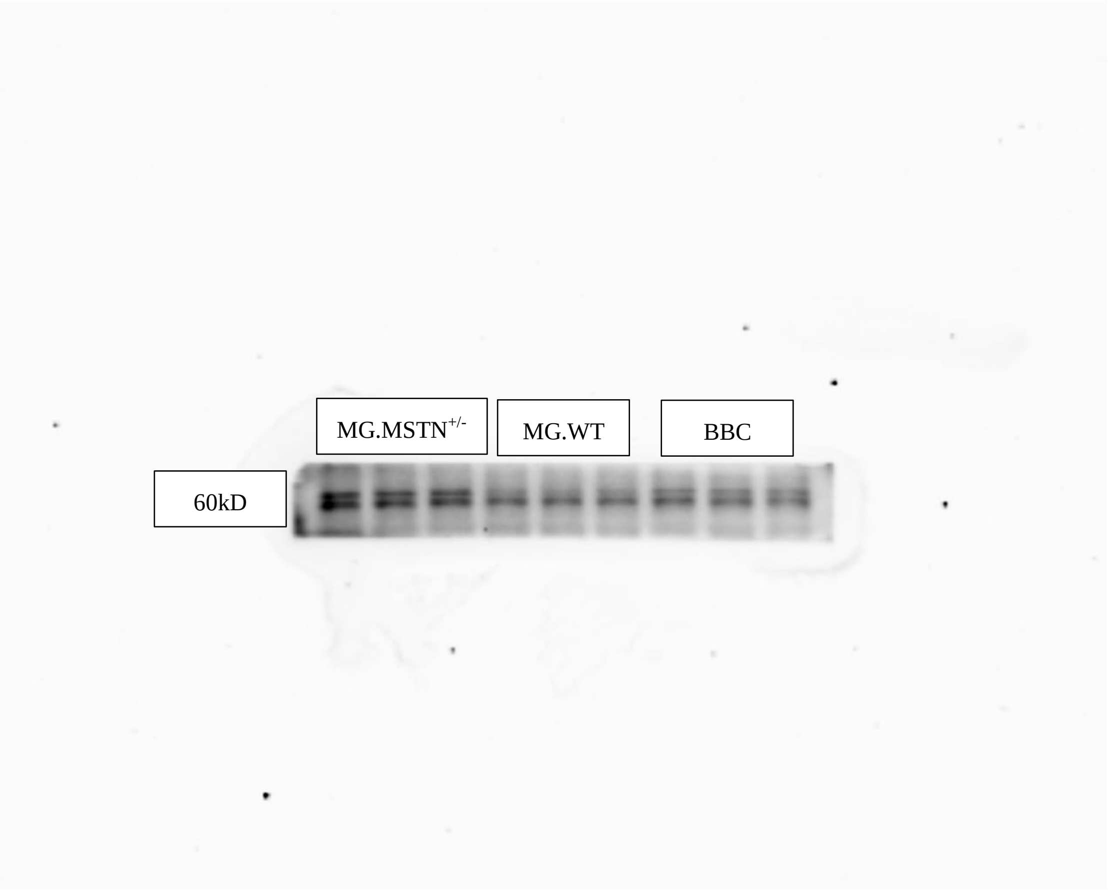

Figure 5, Western blotting results of p-AKT

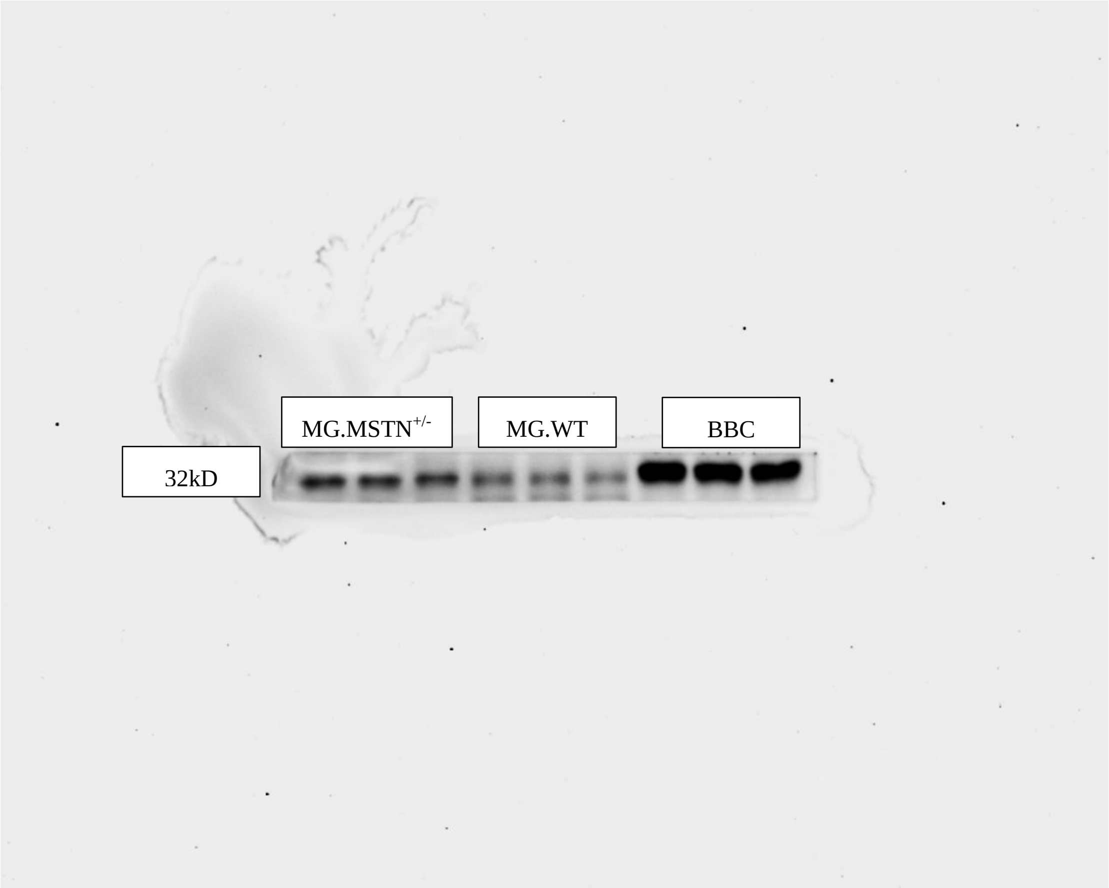

Figure 5, Western blotting results of p-RPS6

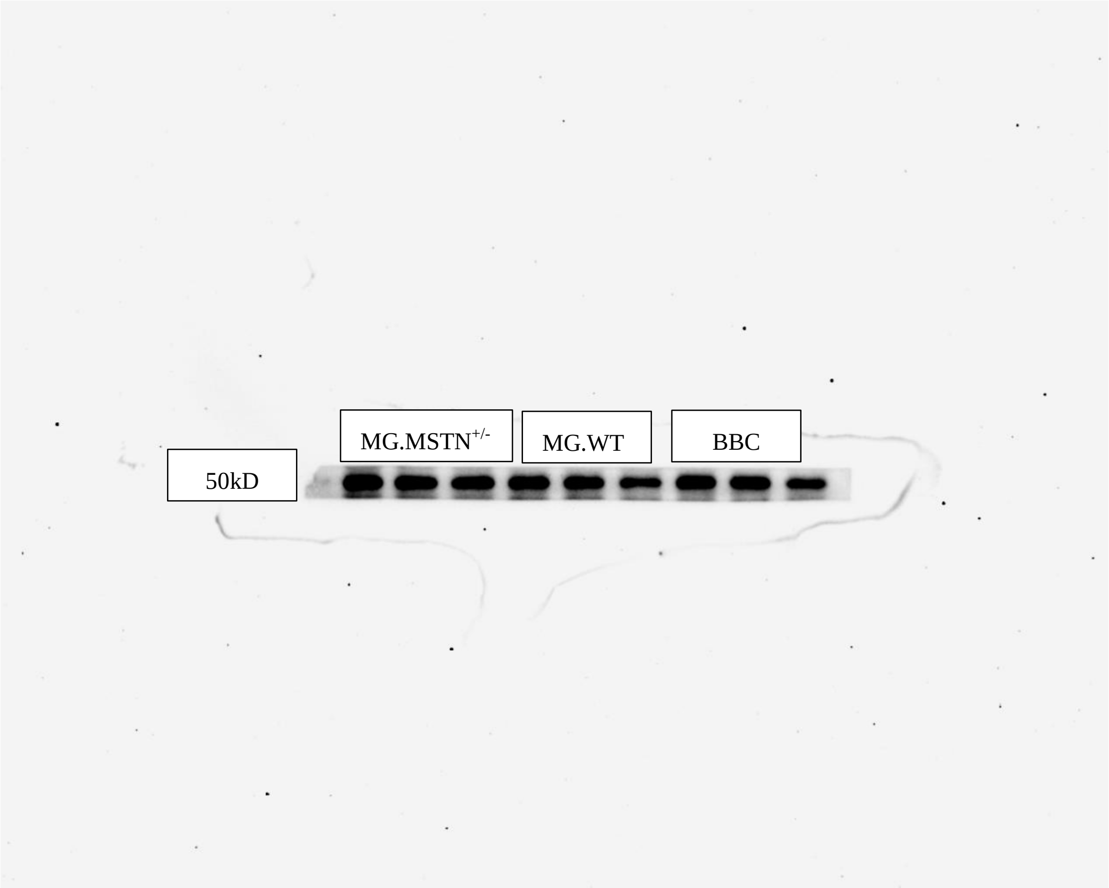

Figure 5, Western blotting results of α-tubulin

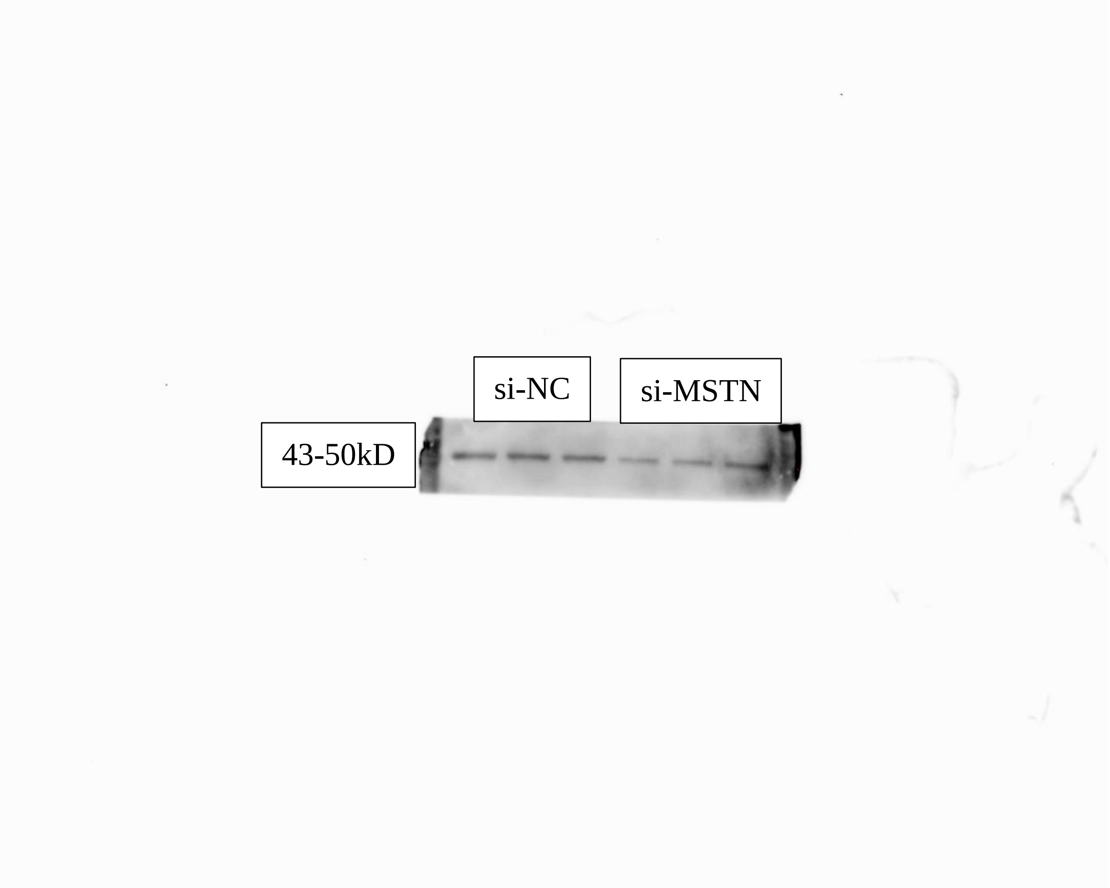

Figure 6-C, Western blotting results of MSTN

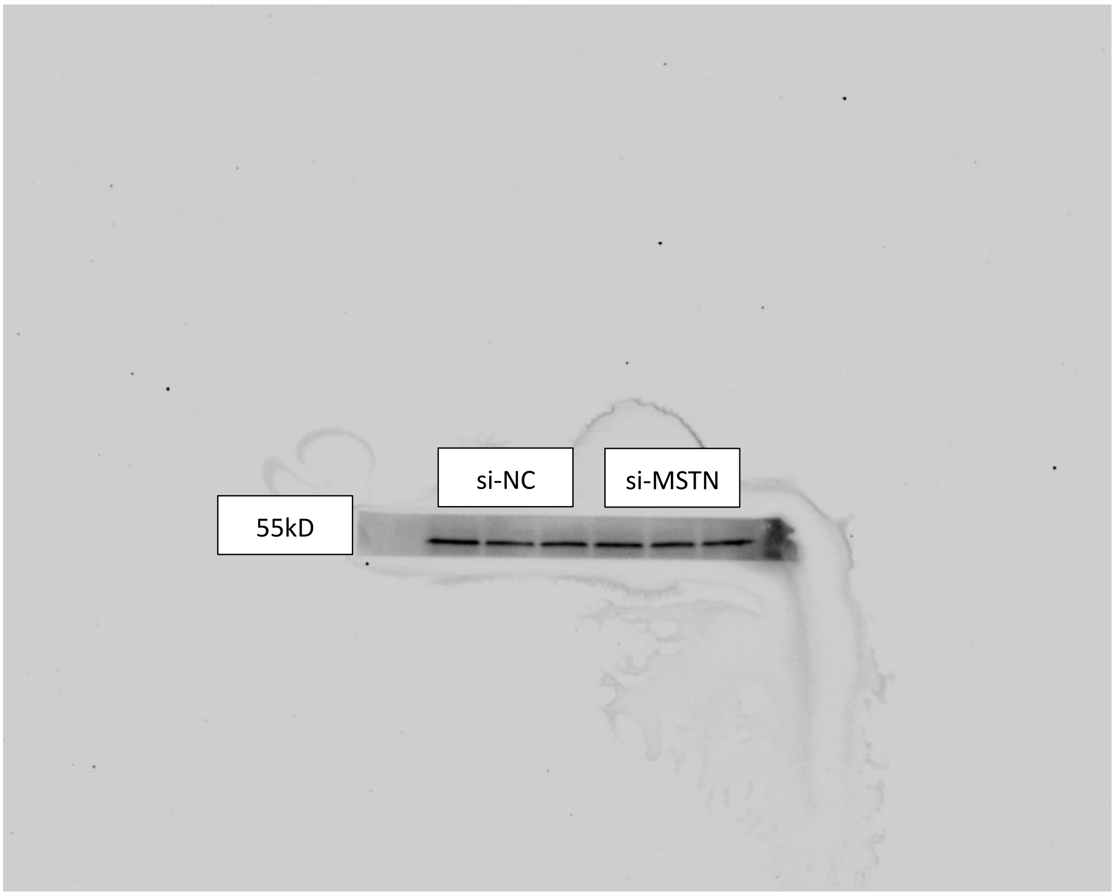

Figure 6-C, Western blotting results of Pax7

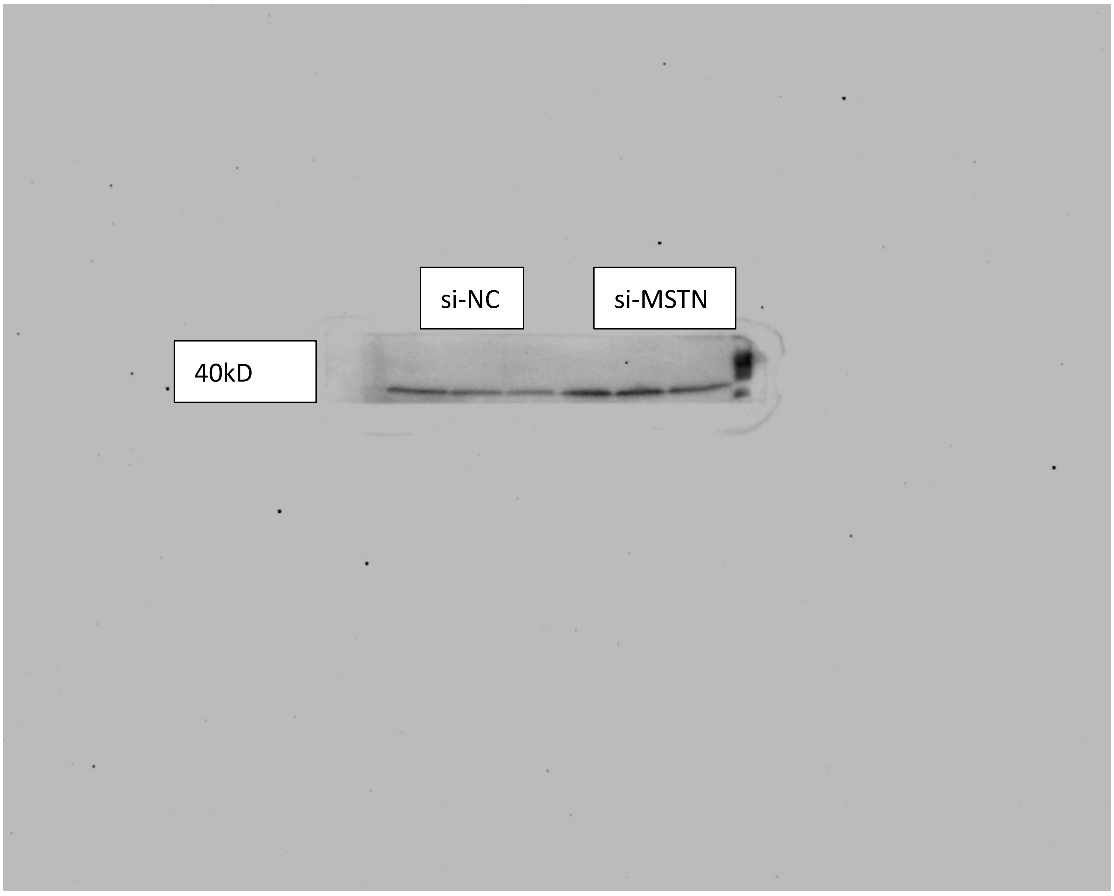

Figure 6-C, Western blotting results of MyoD

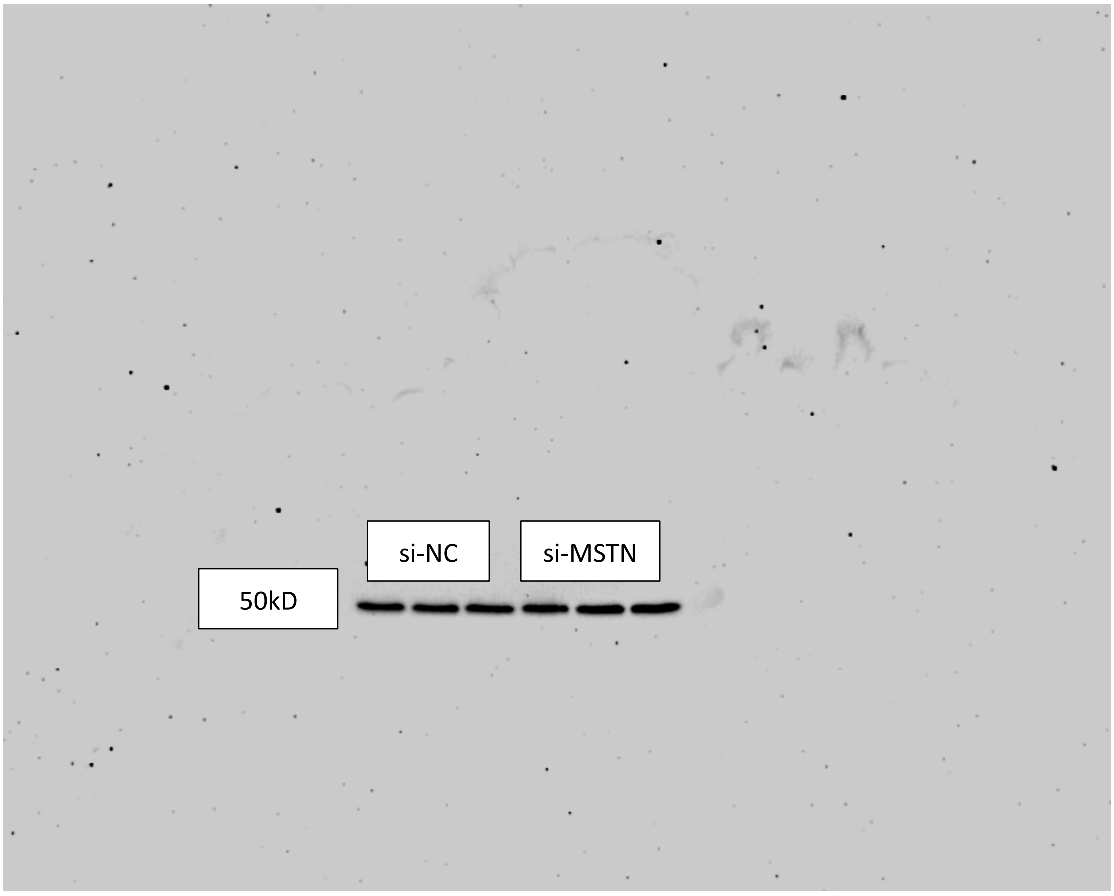

Figure 6-C, Western blotting results of α-tubulin

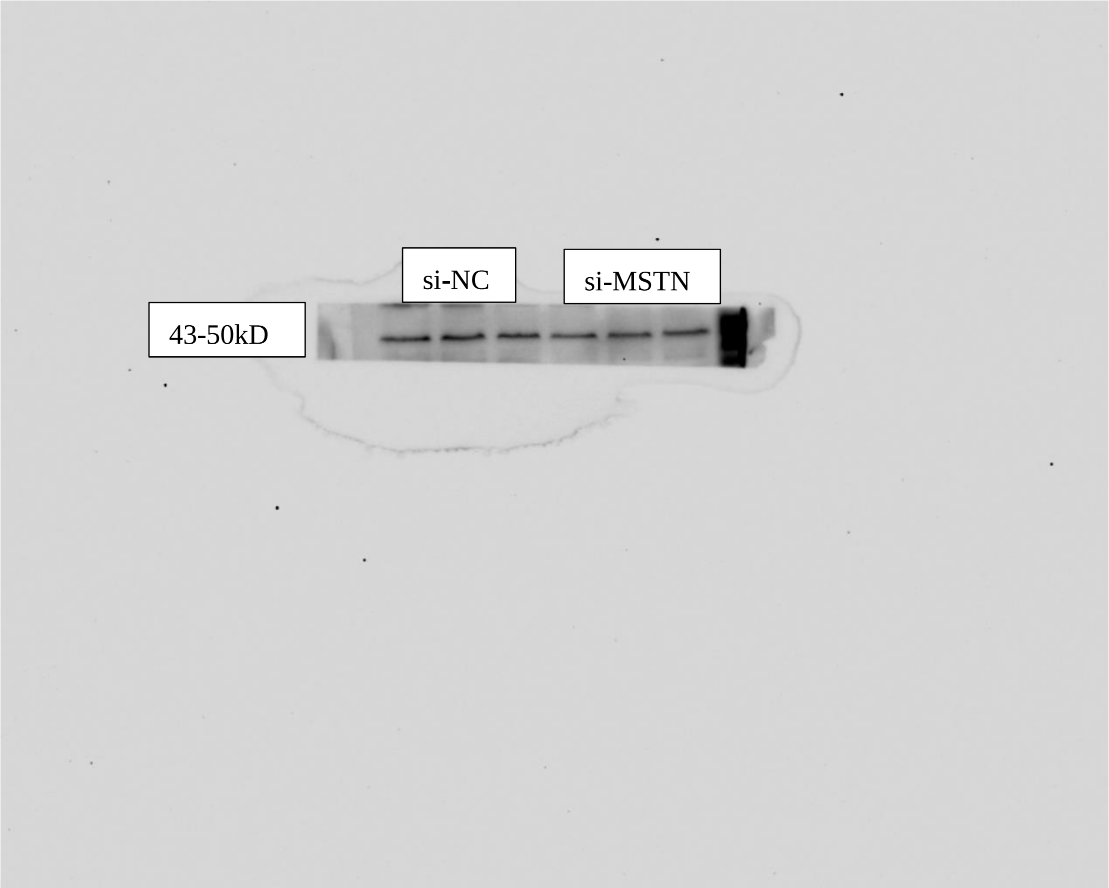

Figure 7-C, Western blotting results of MSTN

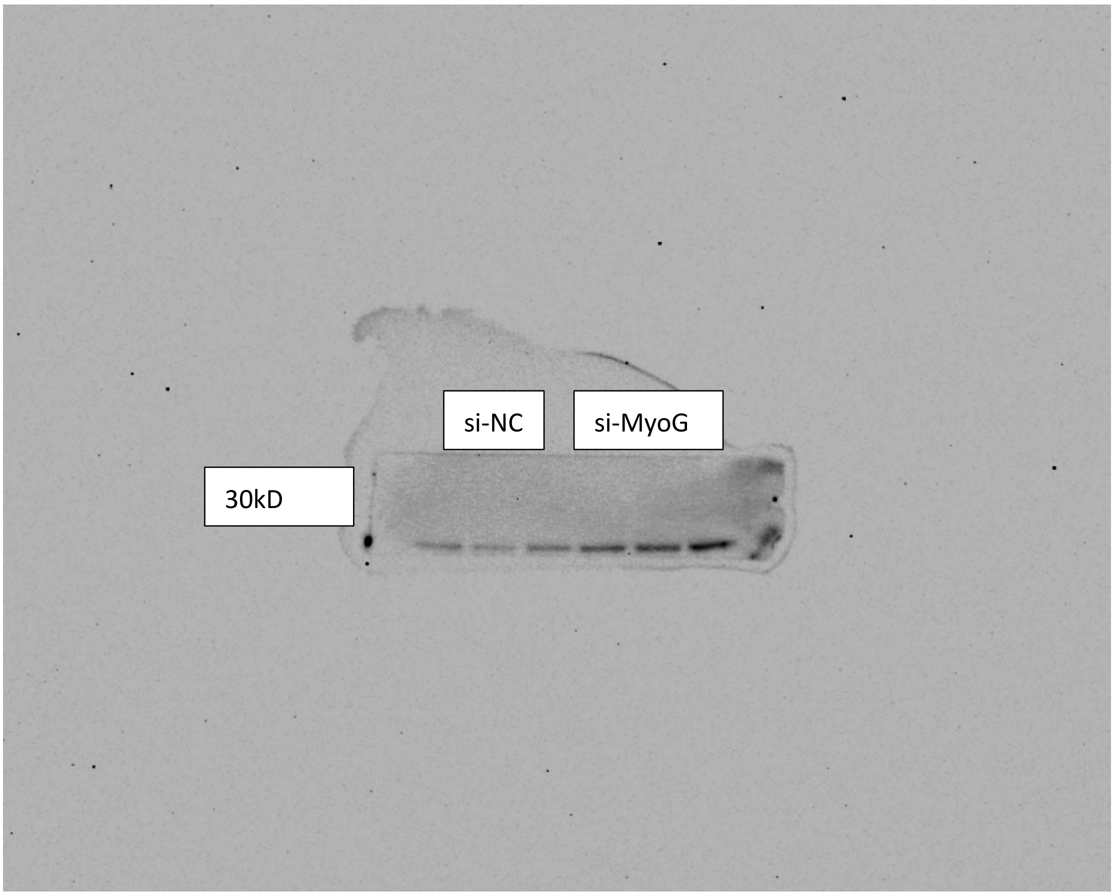

Figure 7-C, Western blotting results of MyoG

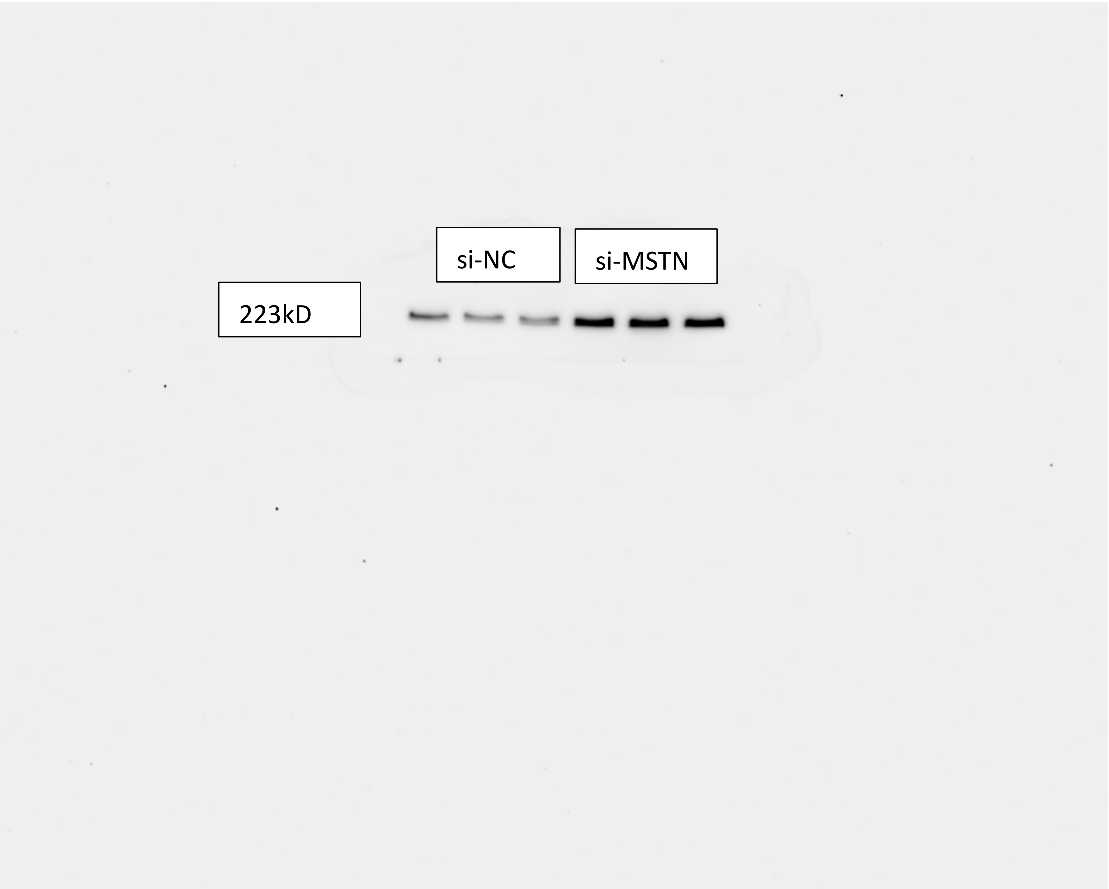

Figure 7-C, Western blotting results of MyHC

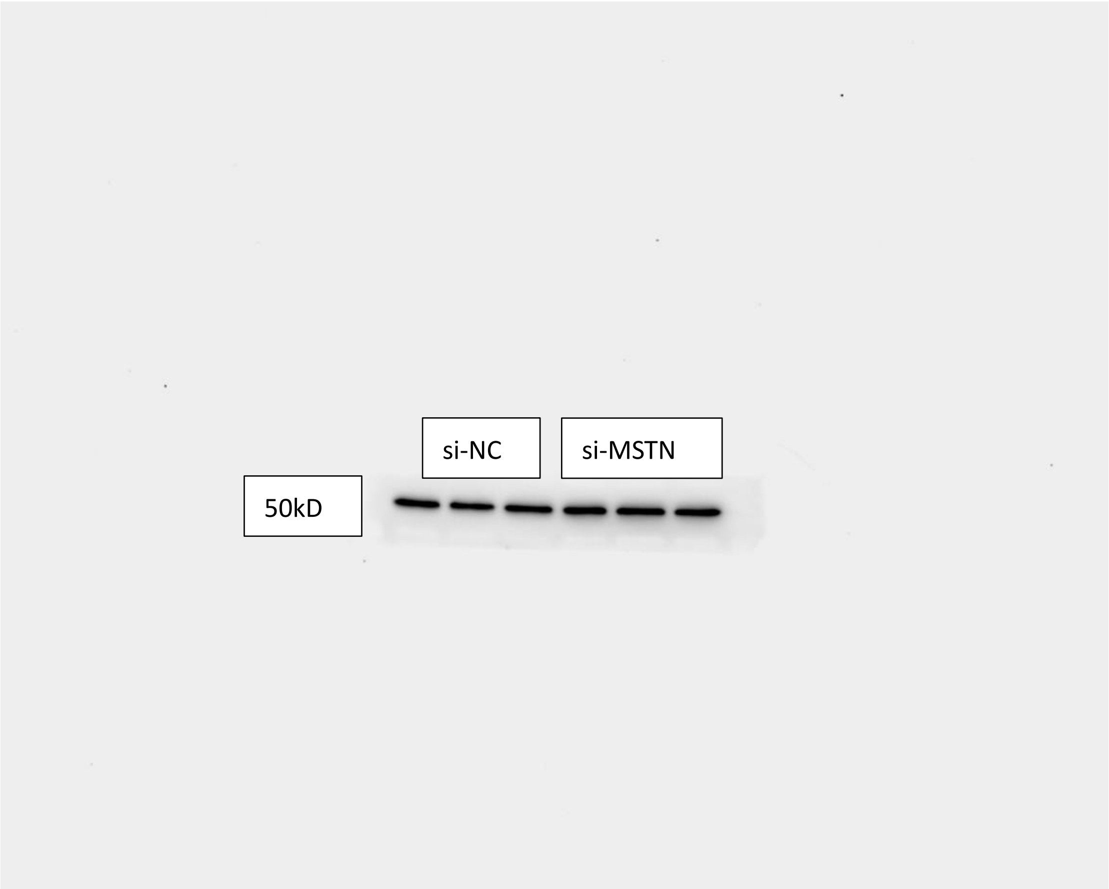

Figure 7-C, Western blotting results of α-tubulin

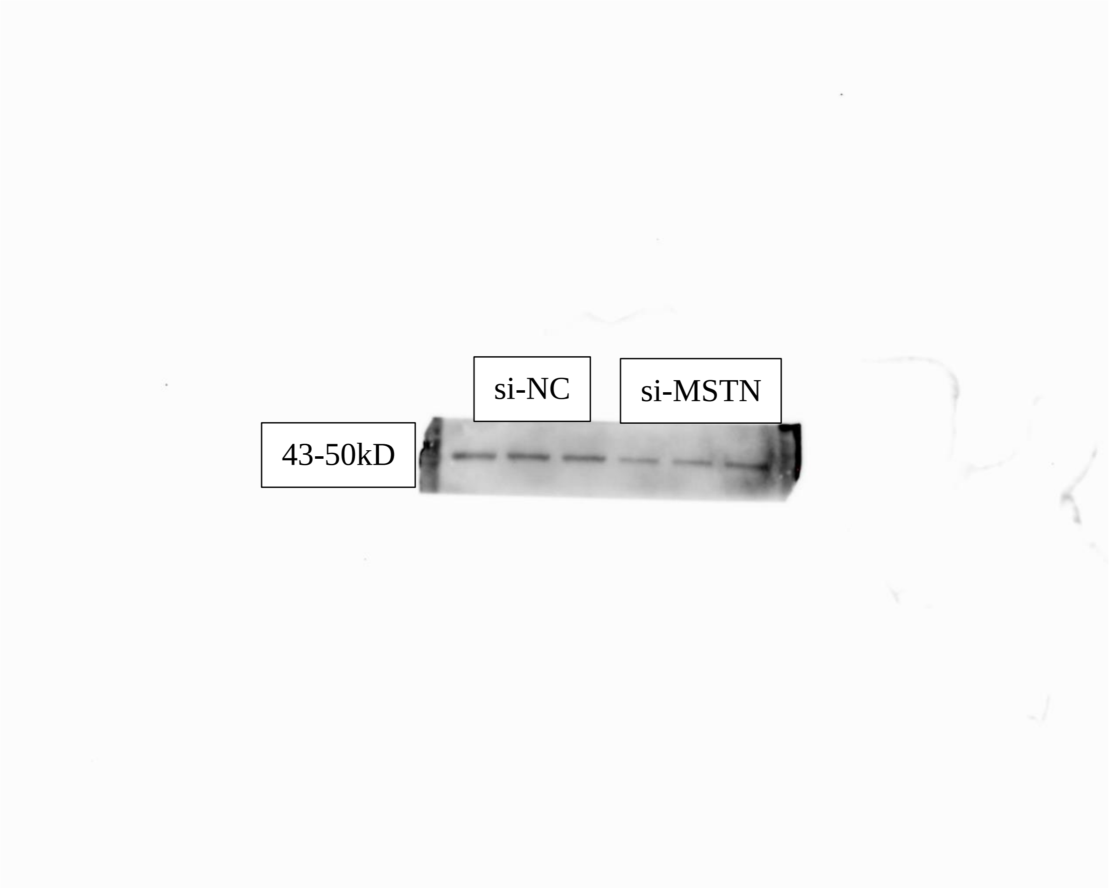

Figure 8-B, Western blotting results of MSTN

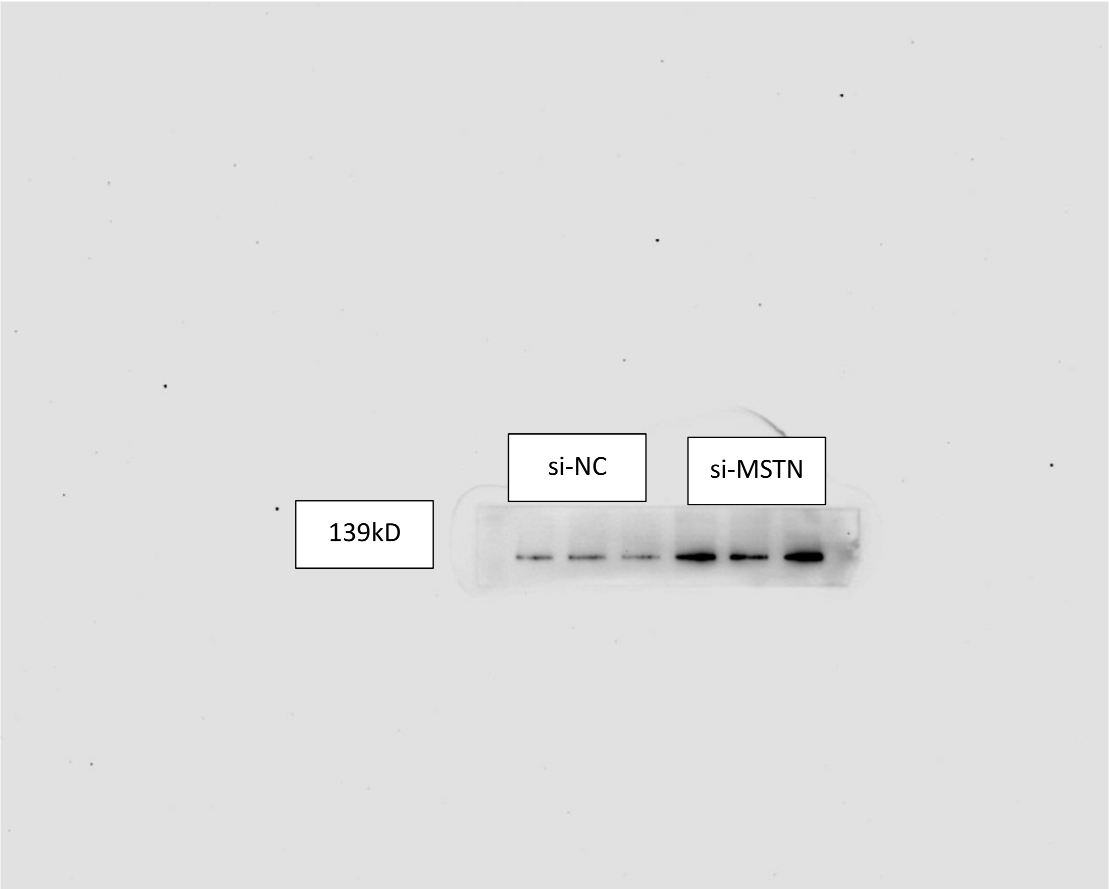

Figure 8-B, Western blotting results of COL1A1

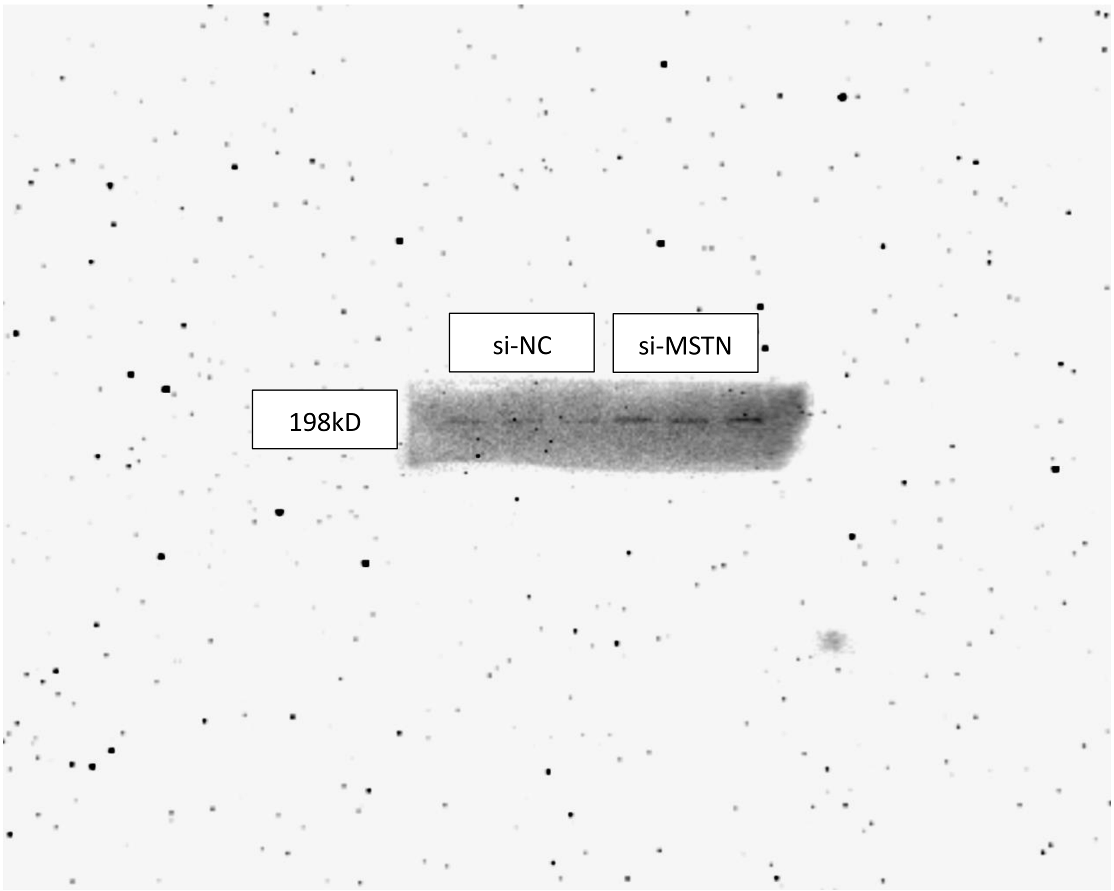

Figure 8-B, Western blotting results of LAMB1

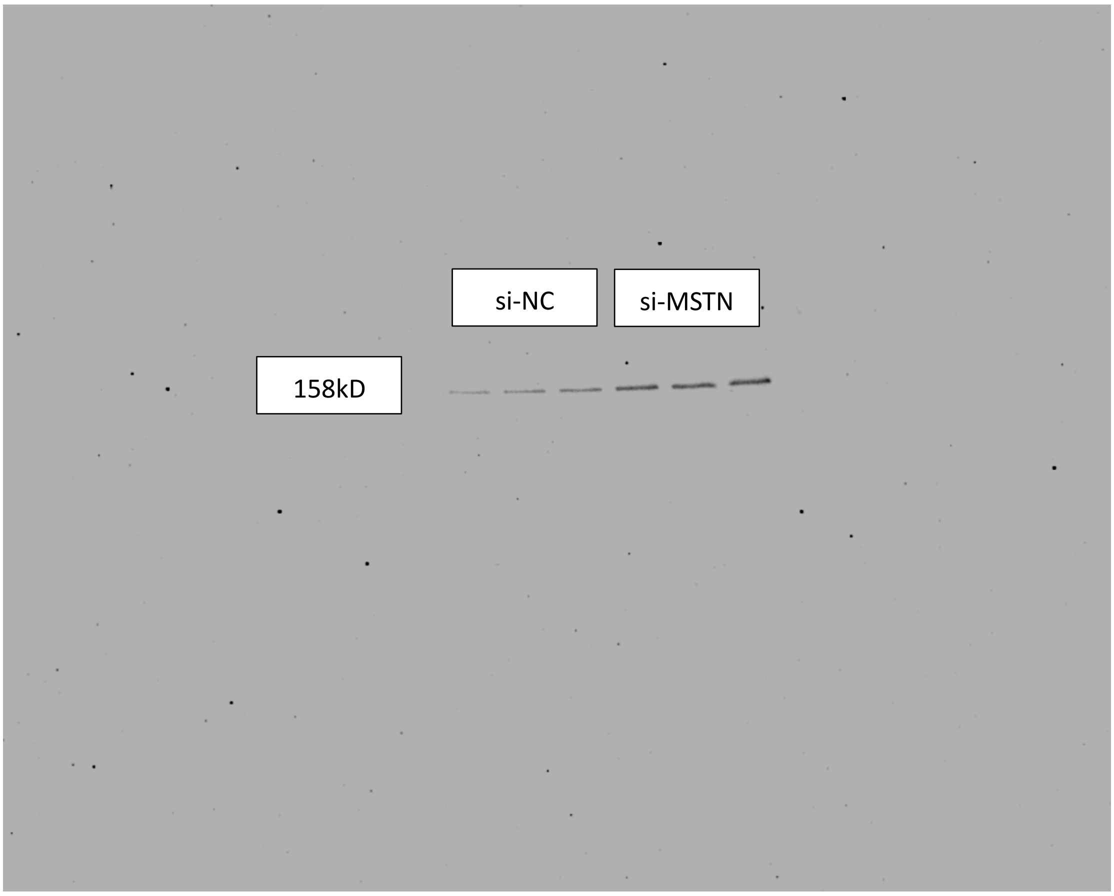

Figure 8-B, Western blotting results of Rock1

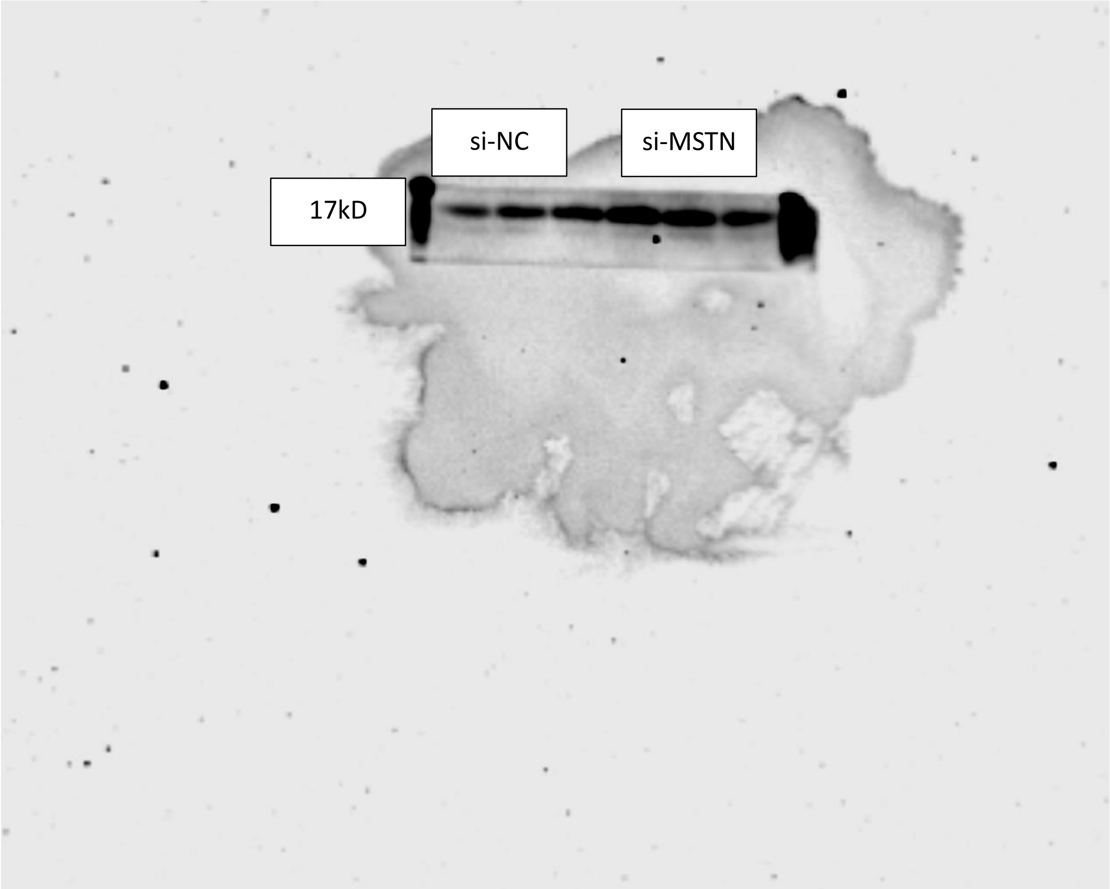

Figure 8-B, Western blotting results of MYL6

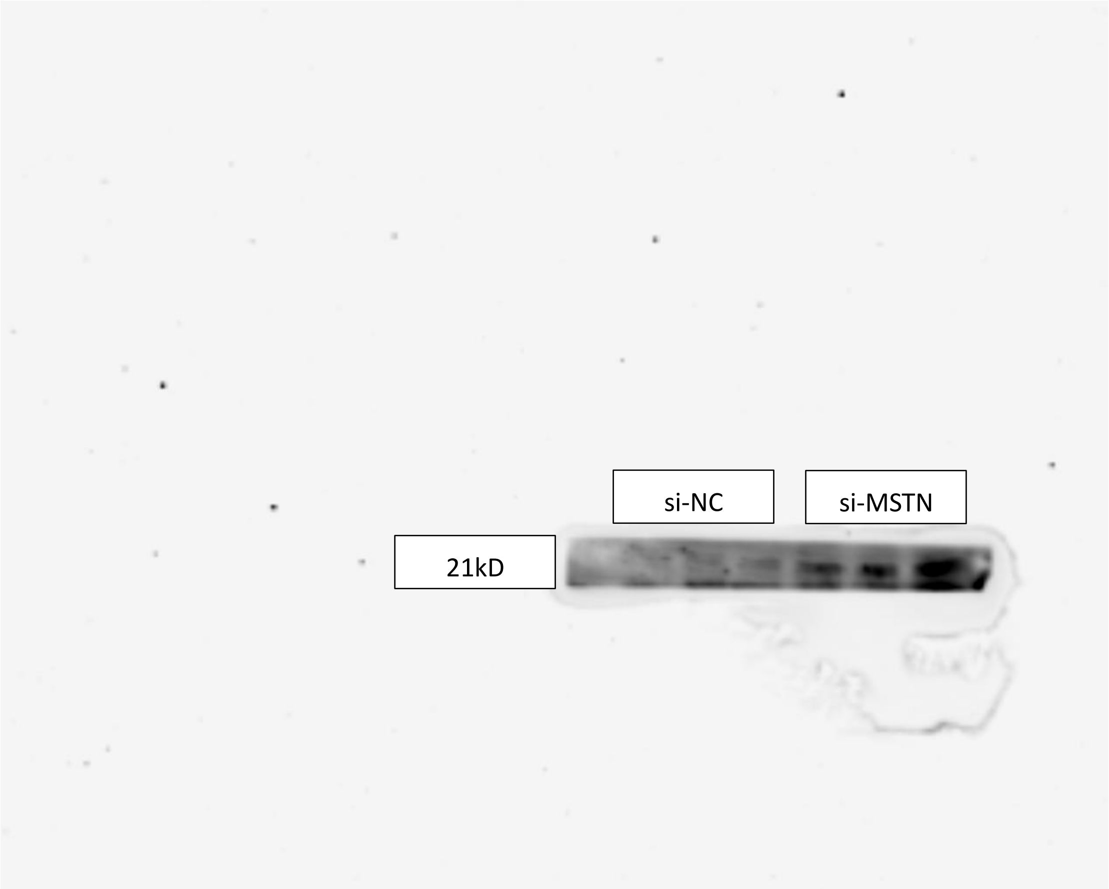

Figure 8-B, Western blotting results of Rac1

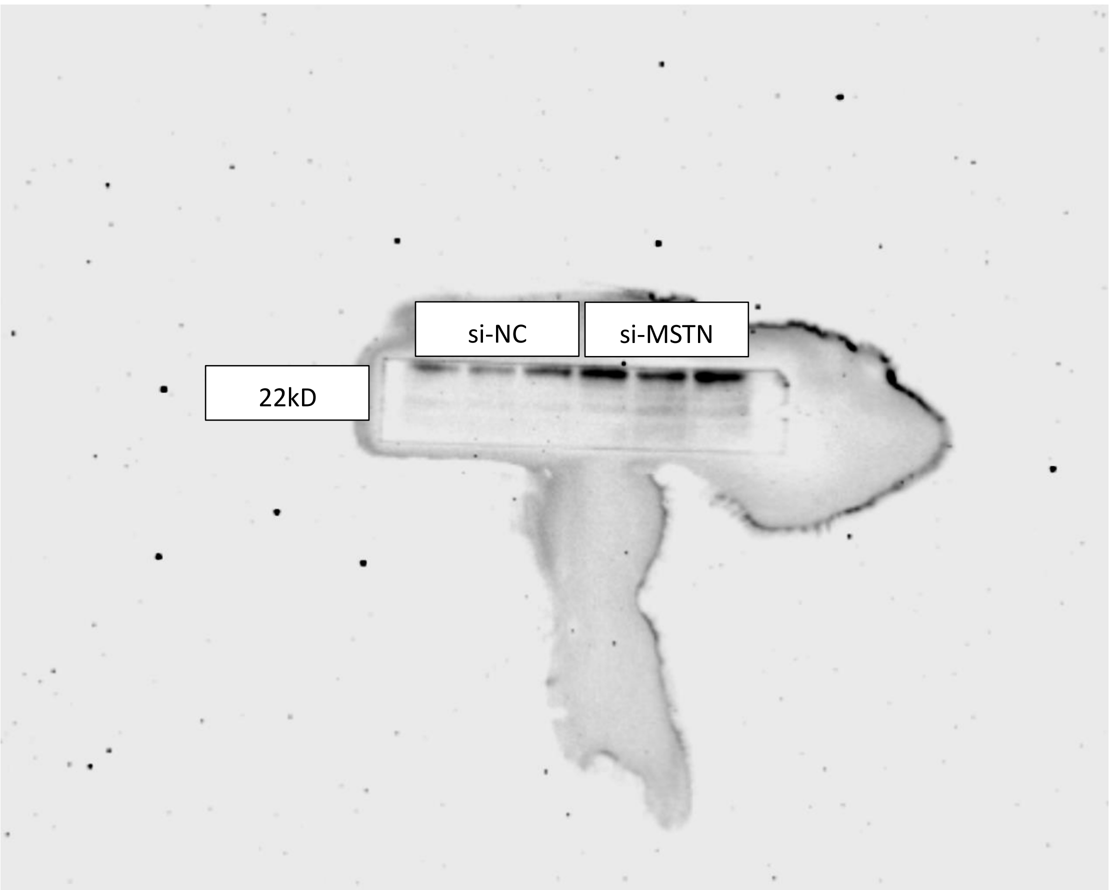

Figure 8-B, Western blotting results of RHoA

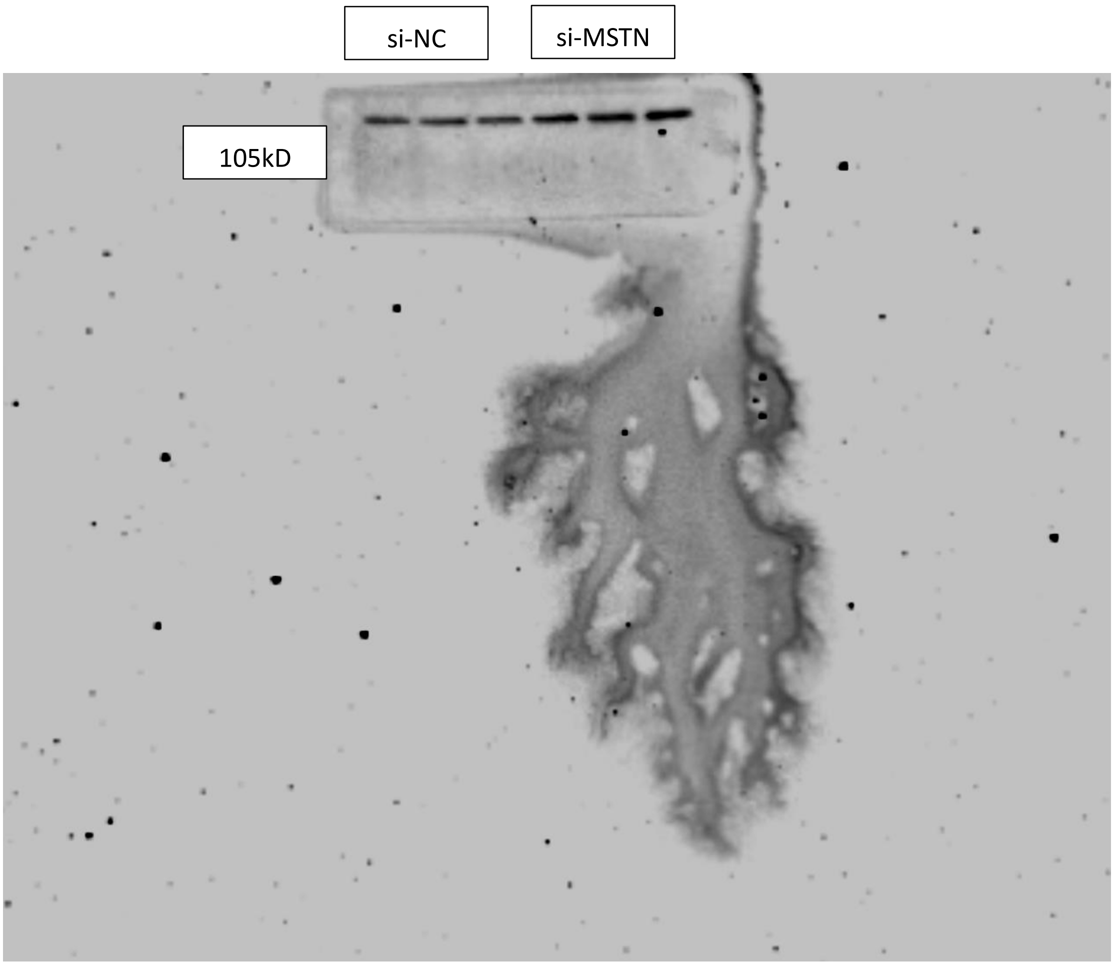

Figure 8-B, Western blotting results of ACTN4

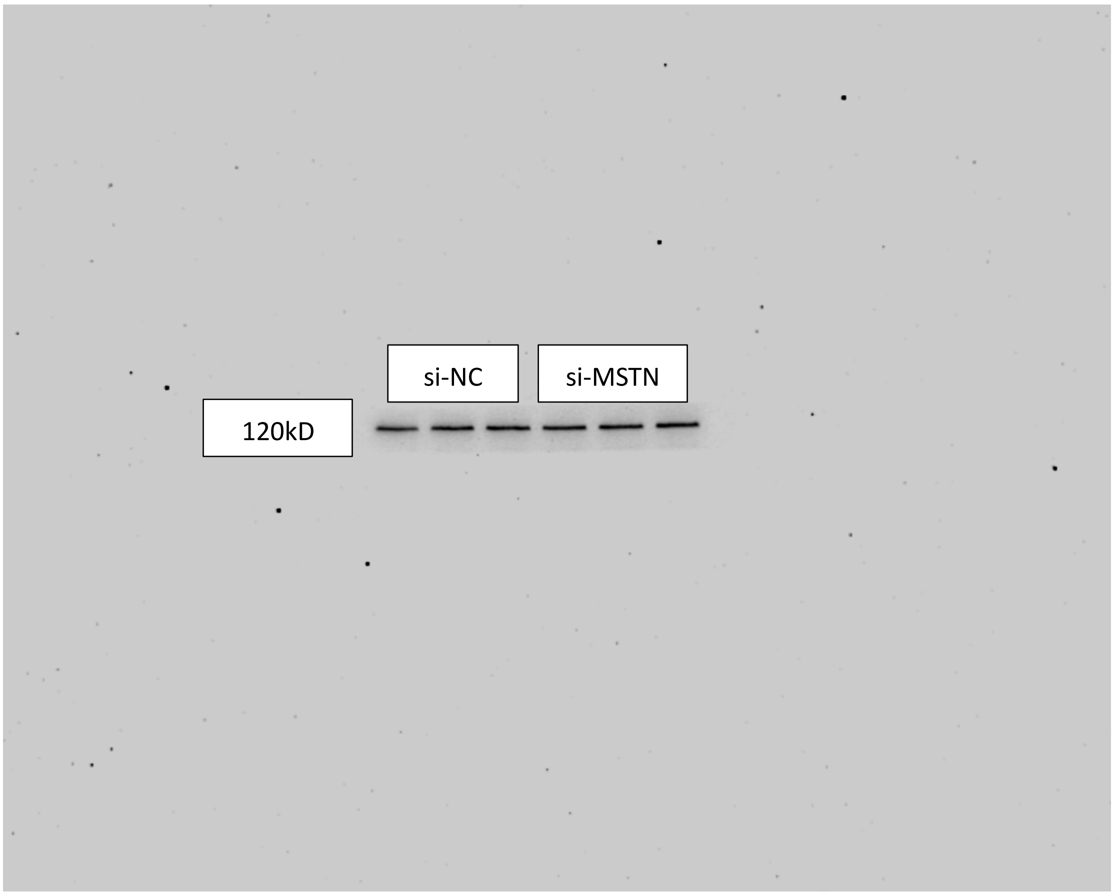

Figure 8-B, Western blotting results of FAK

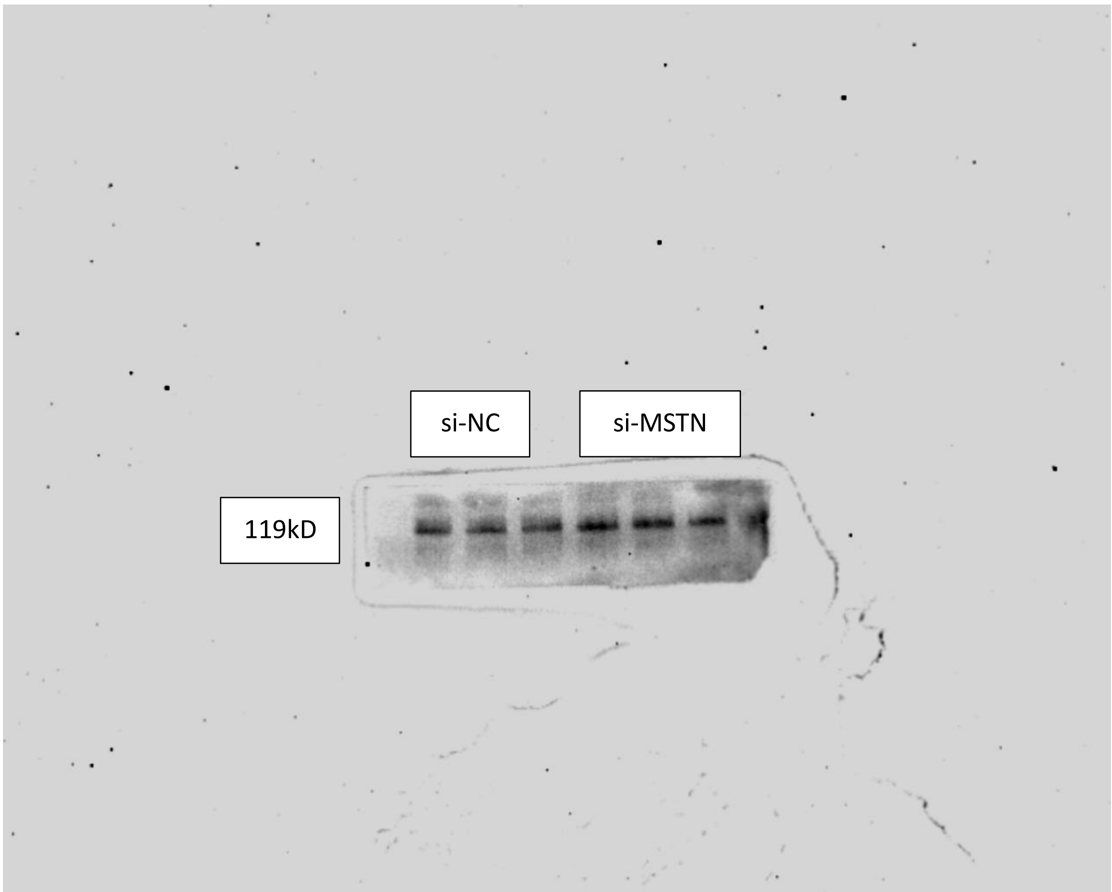

Figure 8-B, Western blotting results of p-FAK

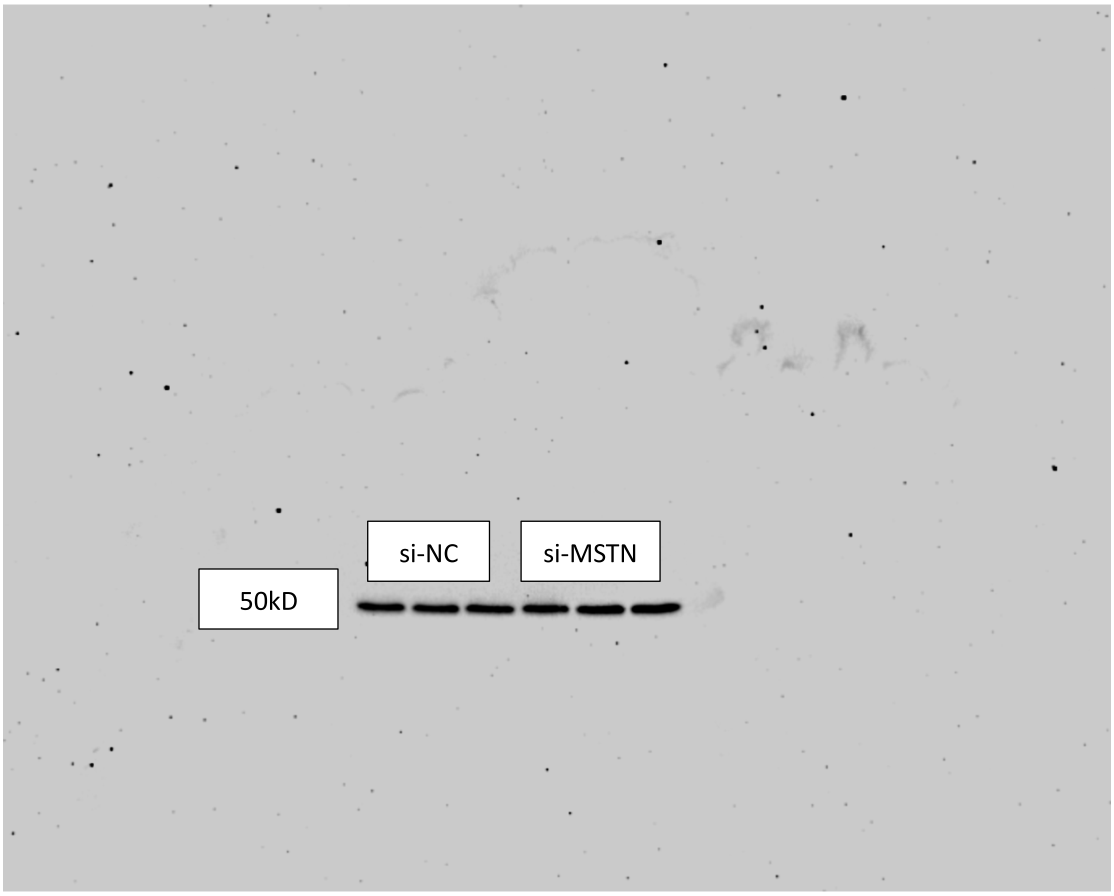

Figure 8-B, Western blotting results of α-tubulin

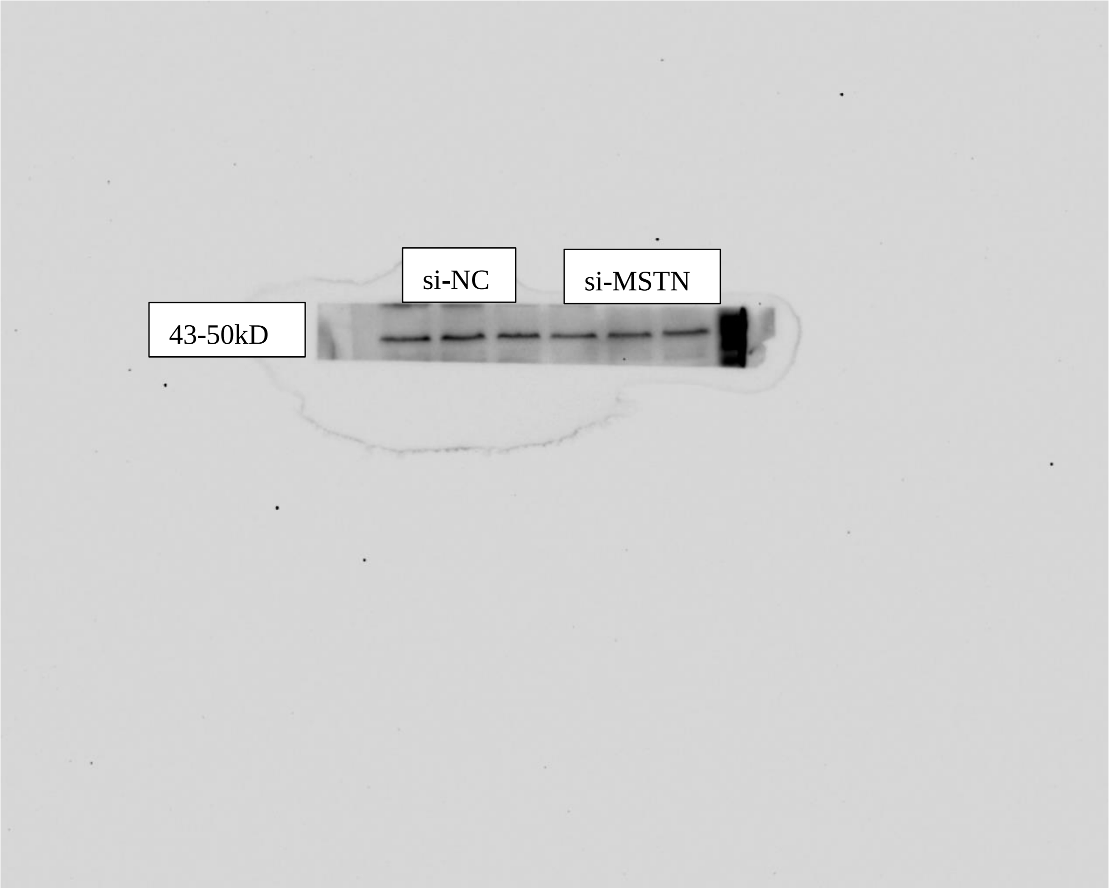

Figure 8-E, Western blotting results of MSTN

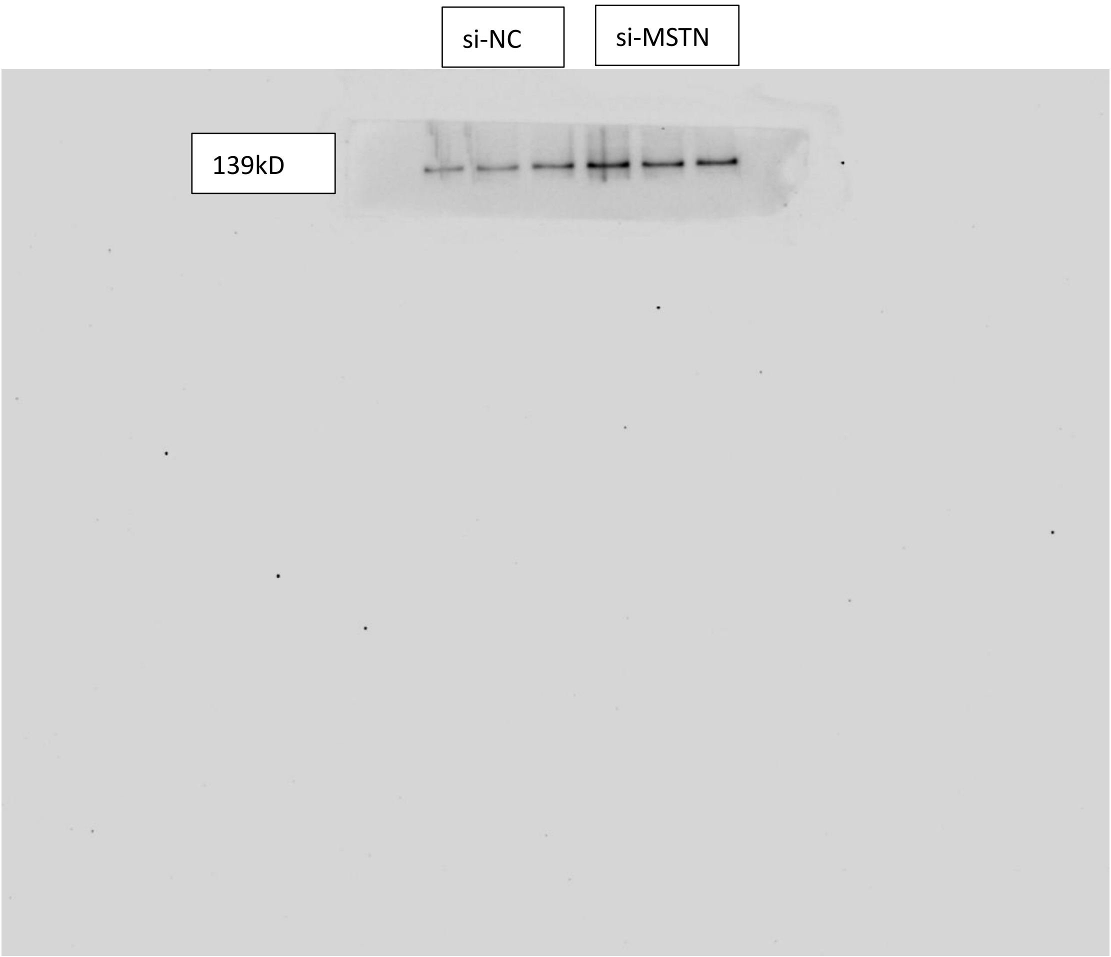

Figure 8-E, Western blotting results of COL1A1

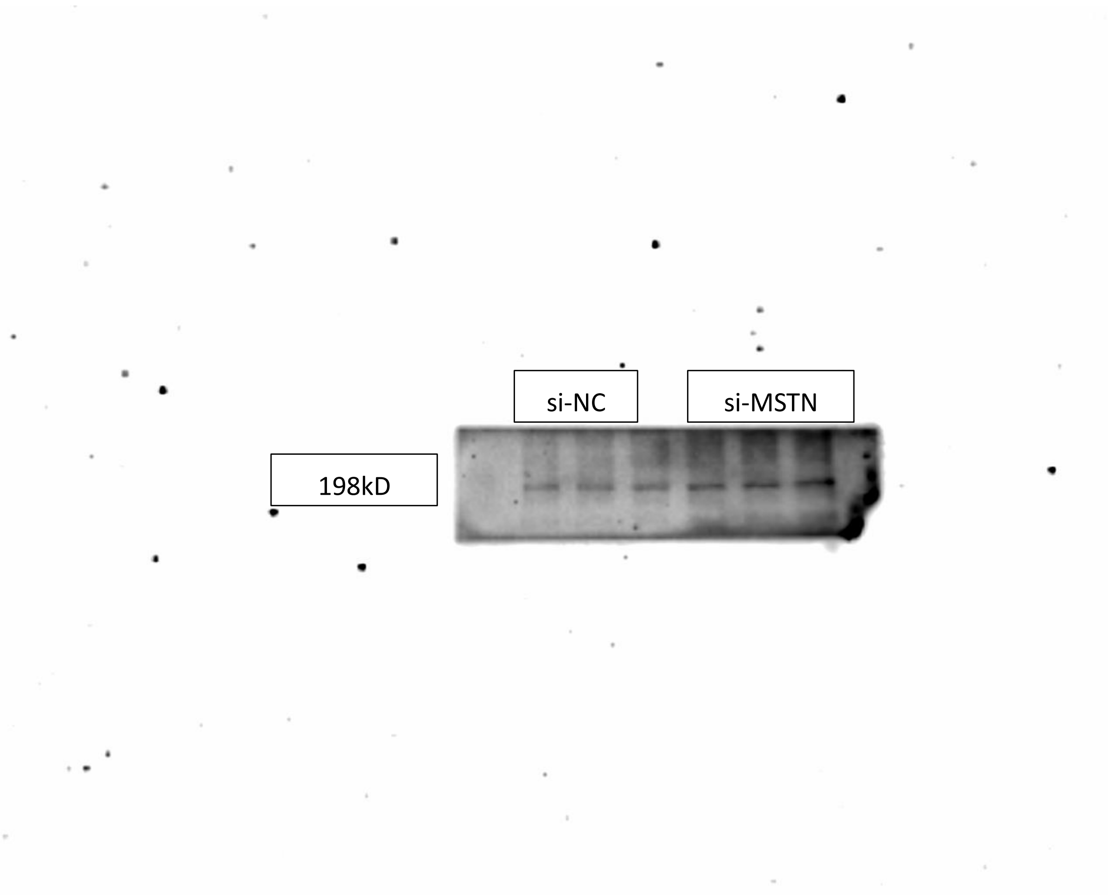

Figure 8-E, Western blotting results of LAMB1

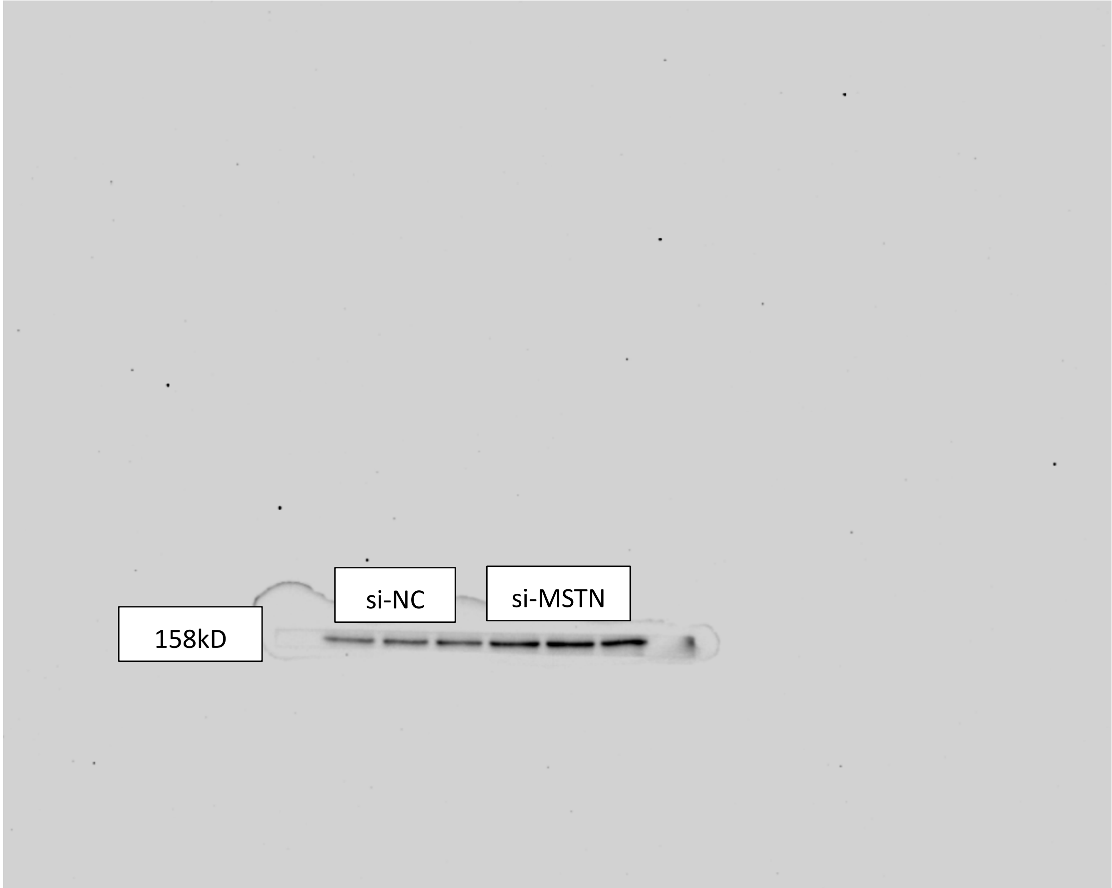

Figure 8-E, Western blotting results of Rock1

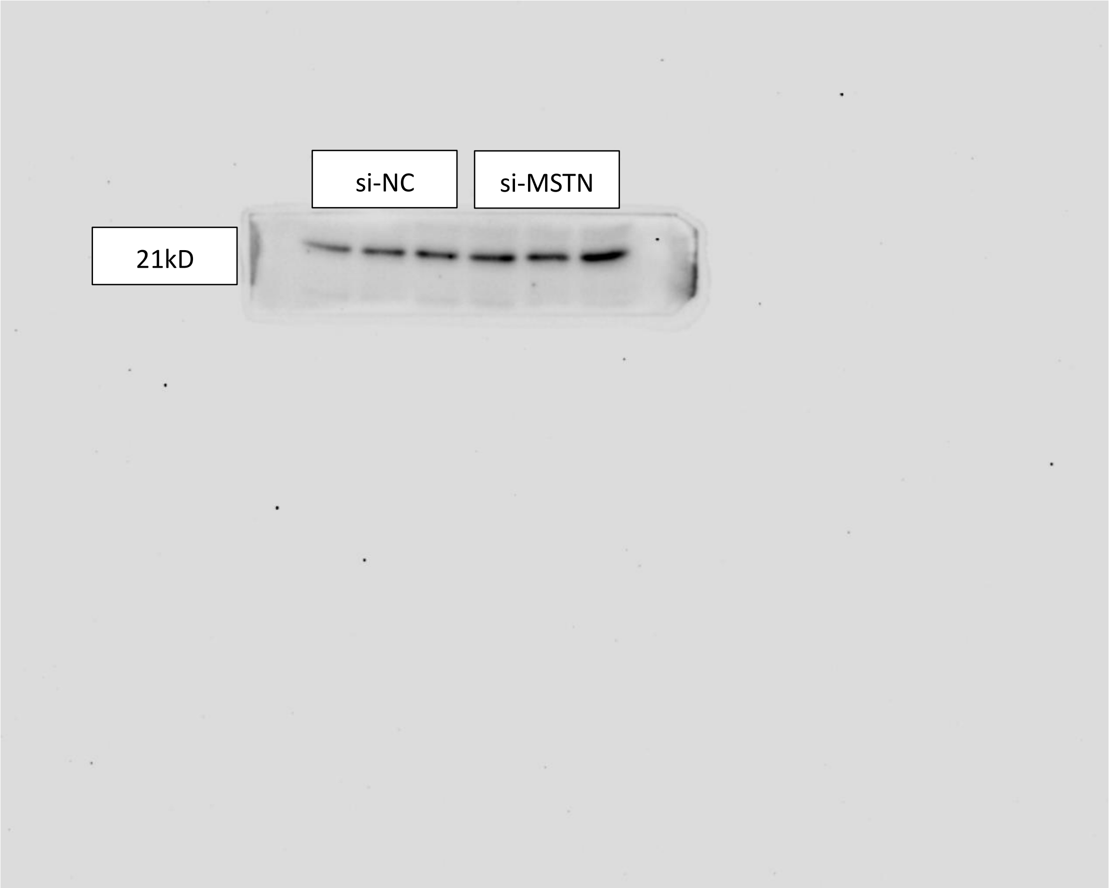

Figure 8-E, Western blotting results of Rac1

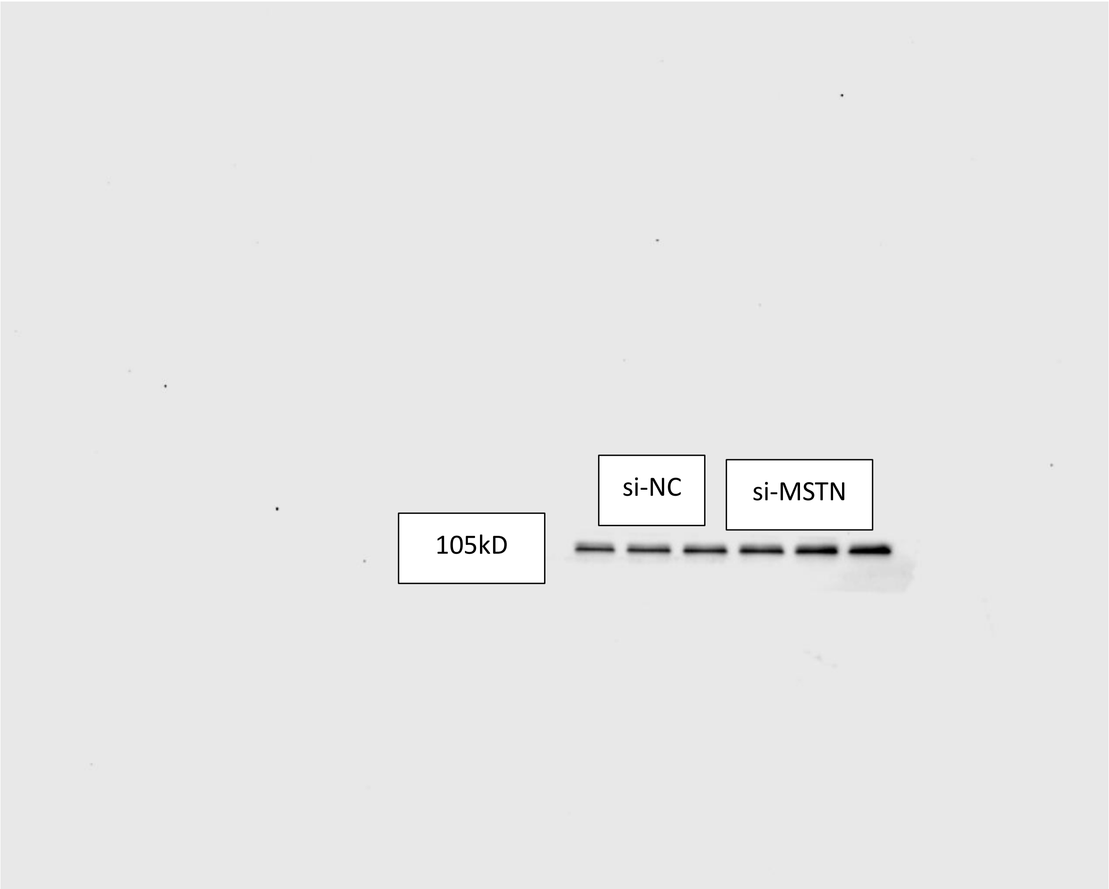

Figure 8-E, Western blotting results of ACTN4

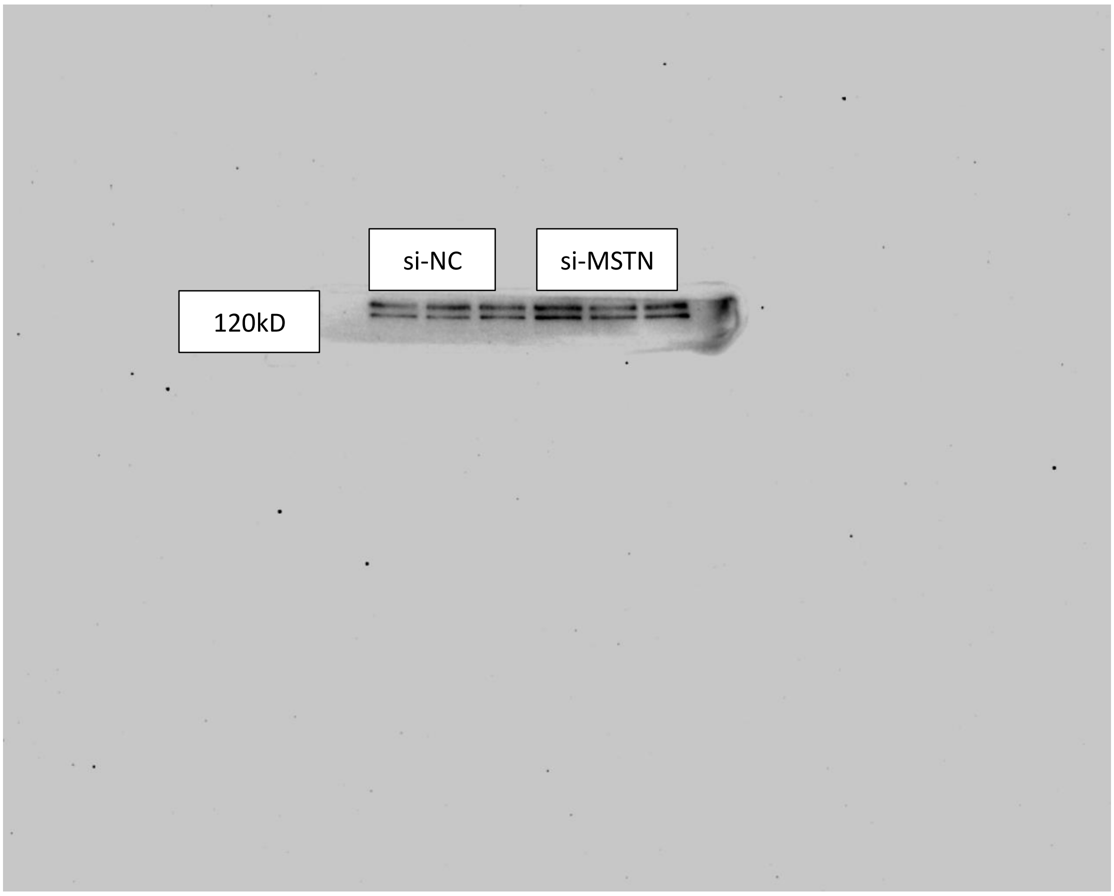

Figure 8-E, Western blotting results of FAK

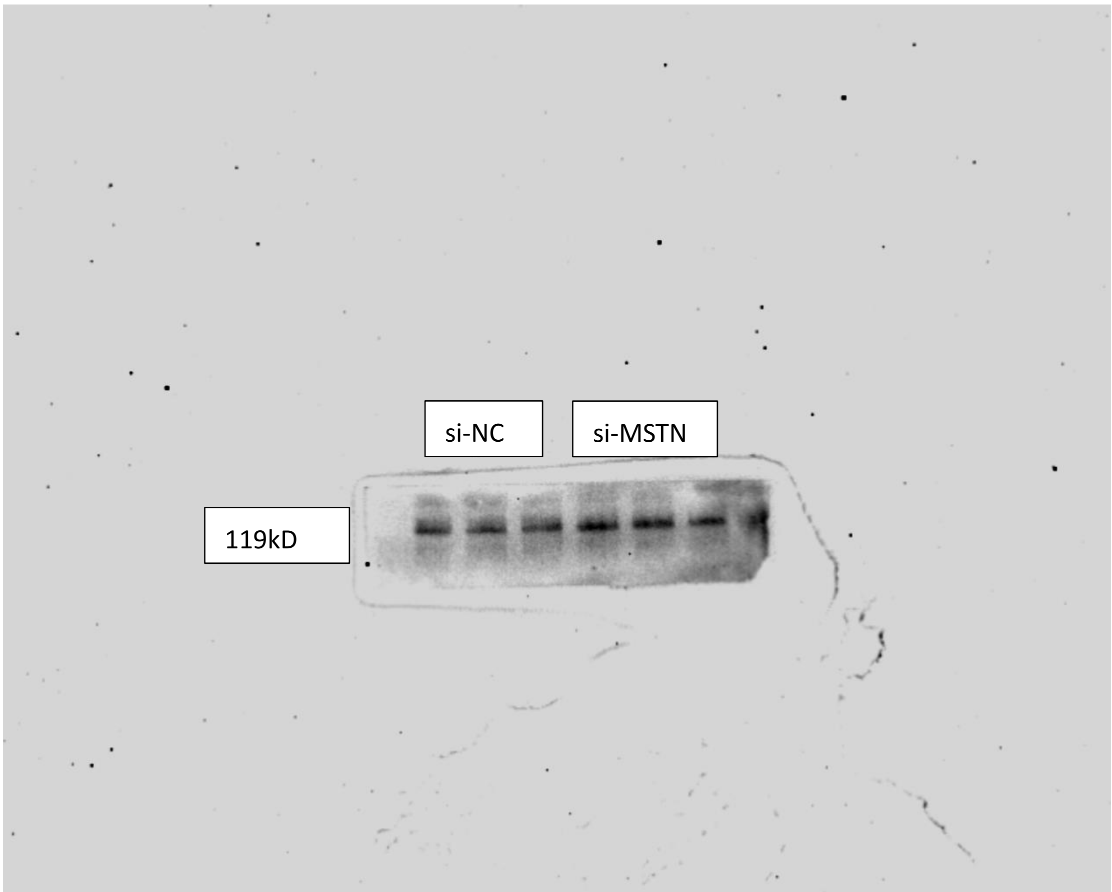

Figure 8-E, Western blotting results of p-FAK

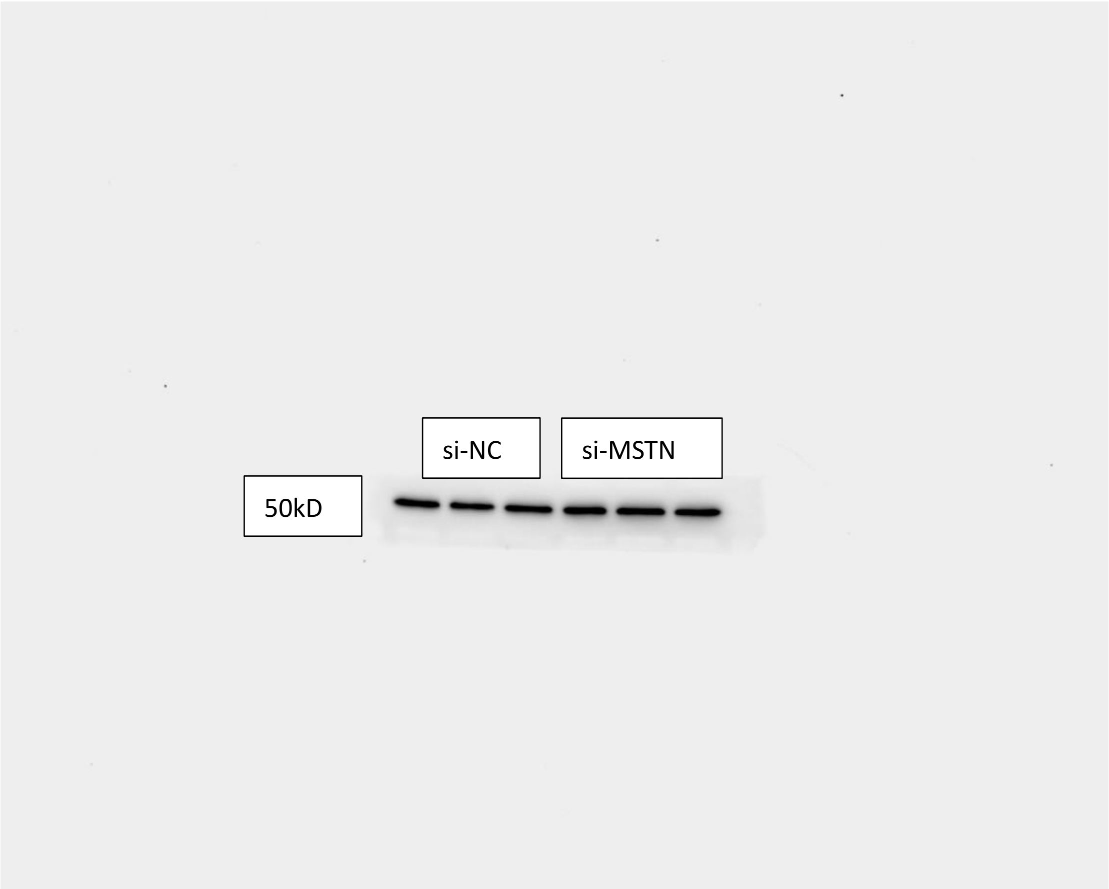

Figure 8-E, Western blotting results of α-tubulin

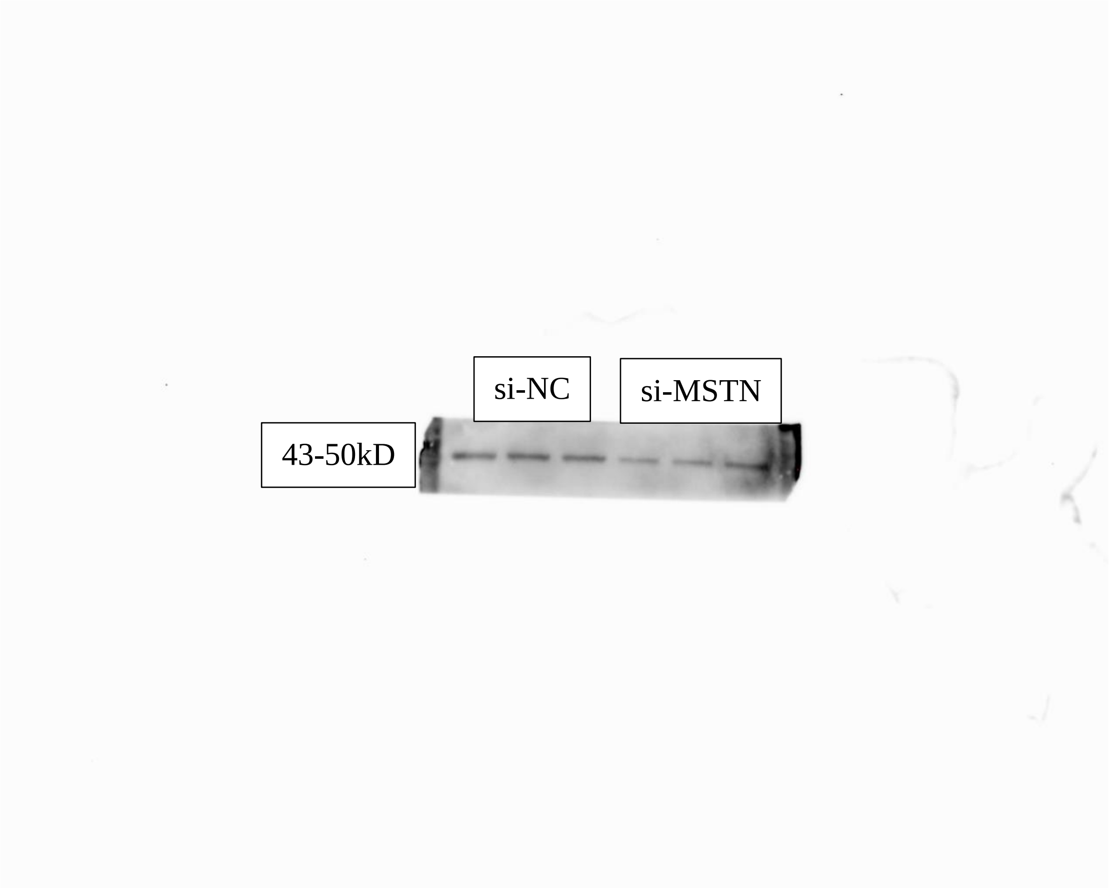

Figure 9-B, Western blotting results of MSTN

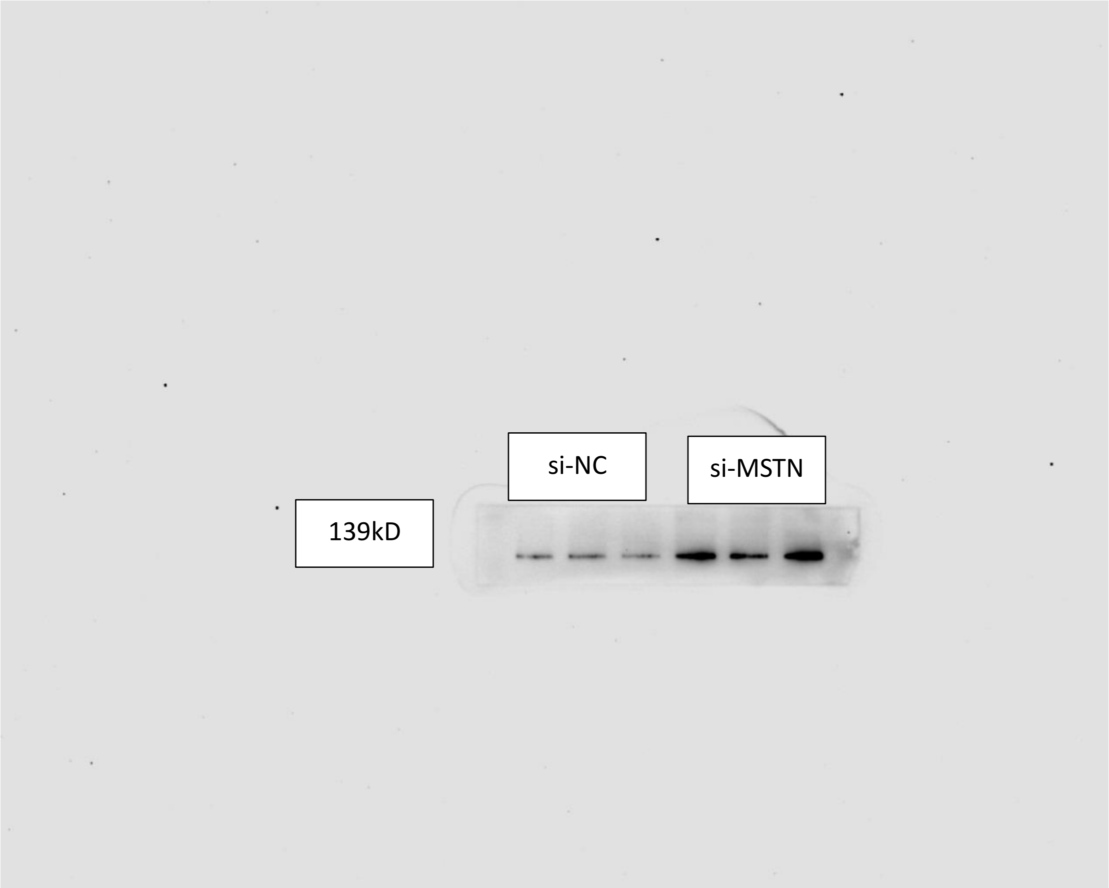

Figure 9-B, Western blotting results of COL1A1

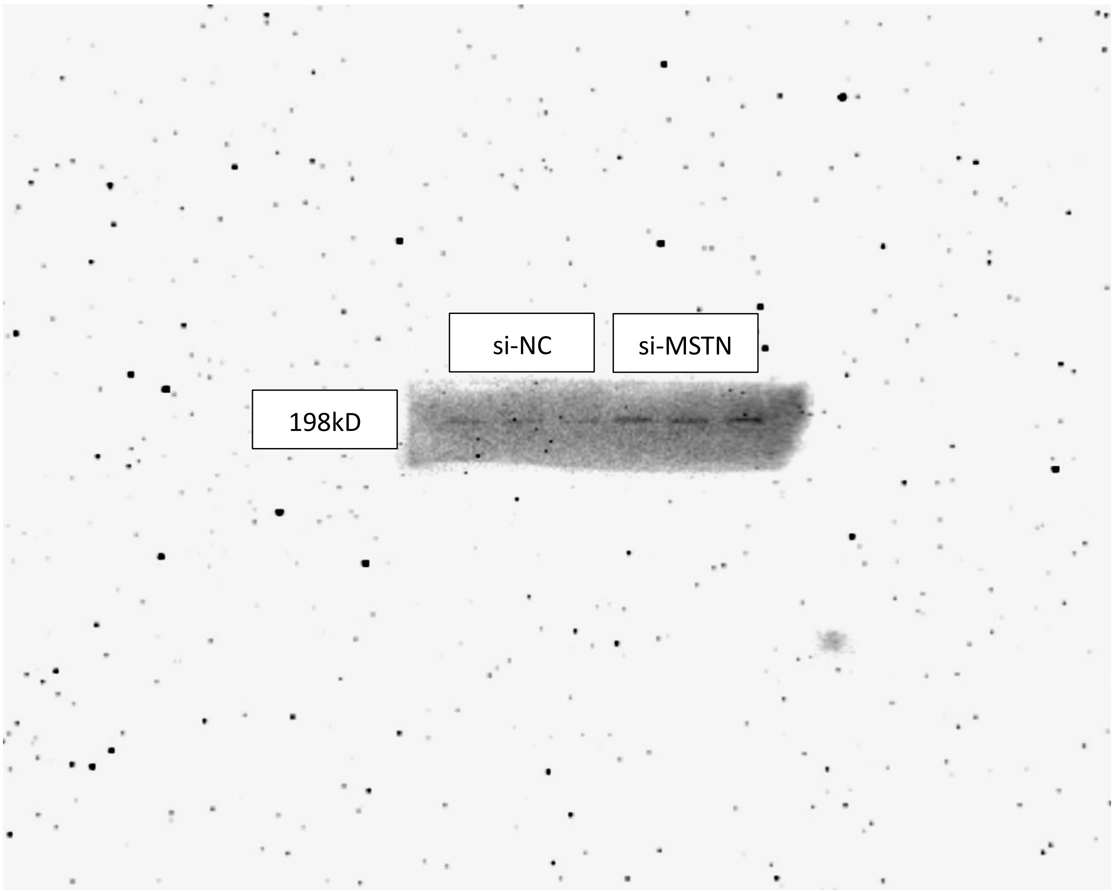

Figure 9-B, Western blotting results of LAMB1

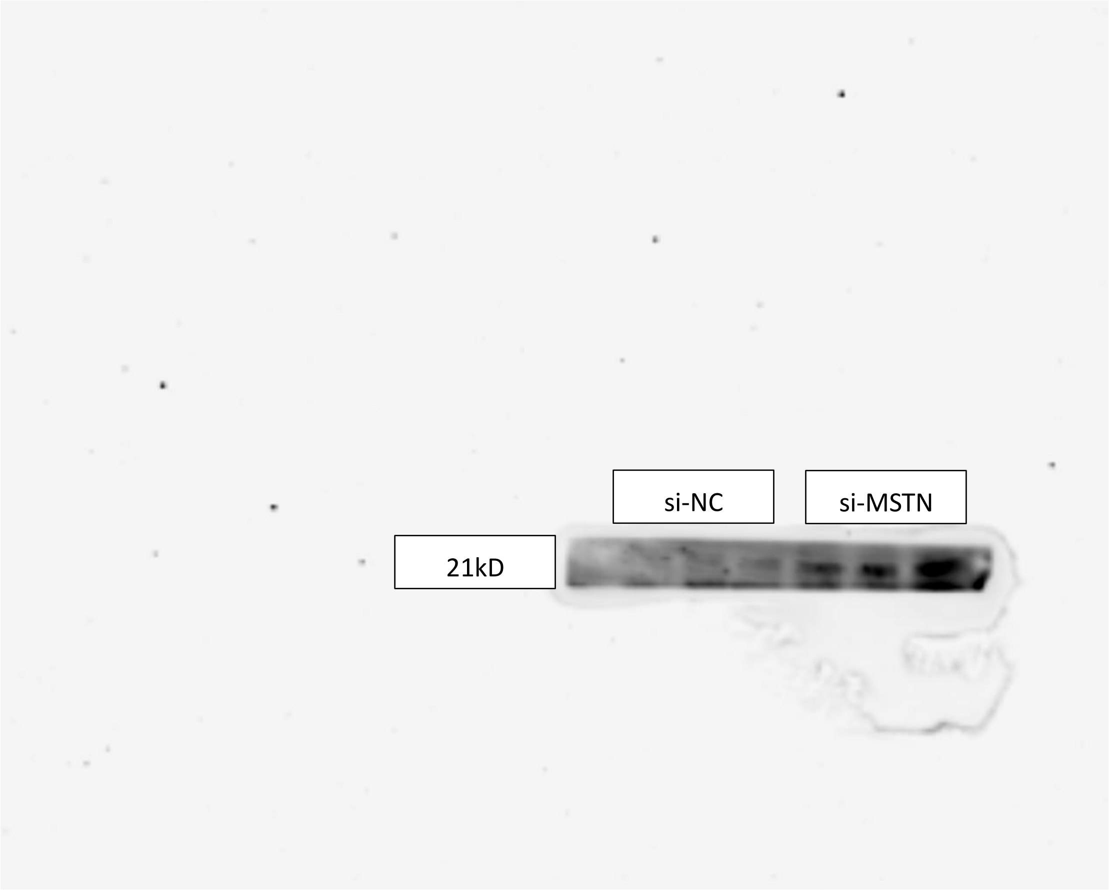

Figure 9-B, Western blotting results of Rac1

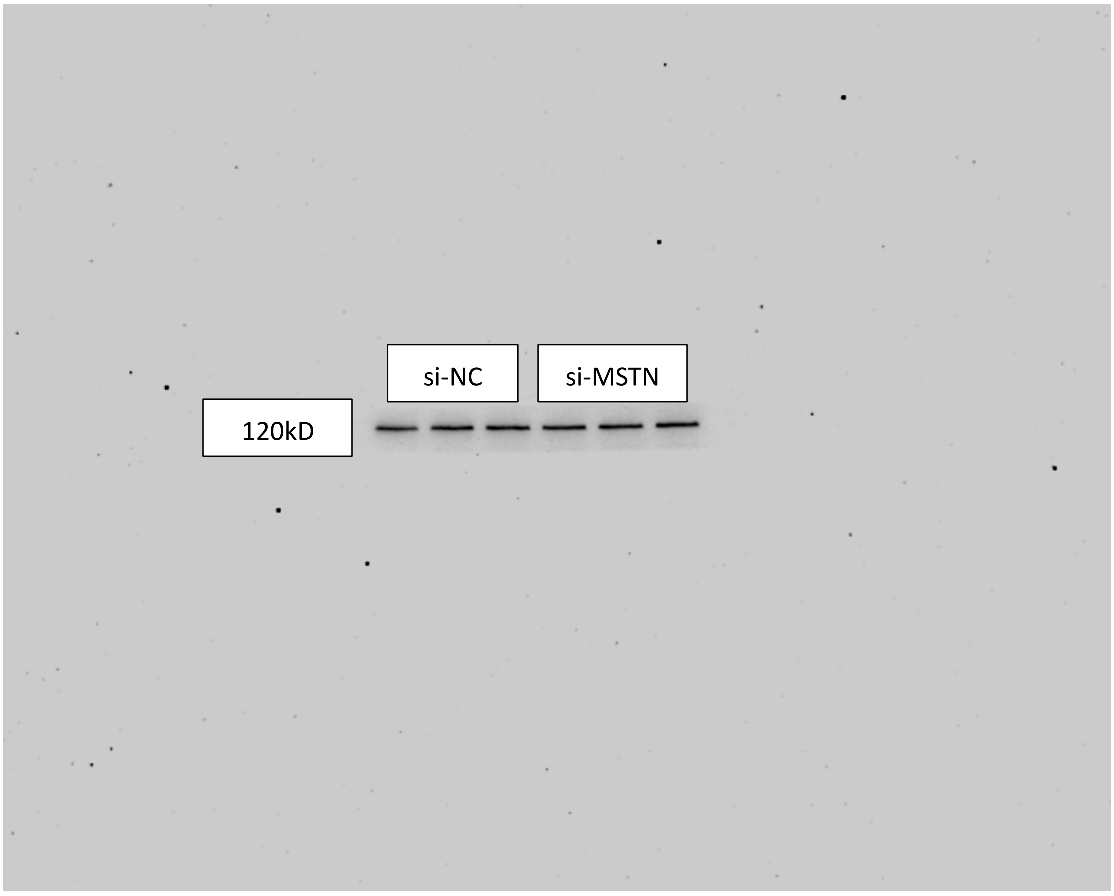

Figure 9-B, Western blotting results of FAK

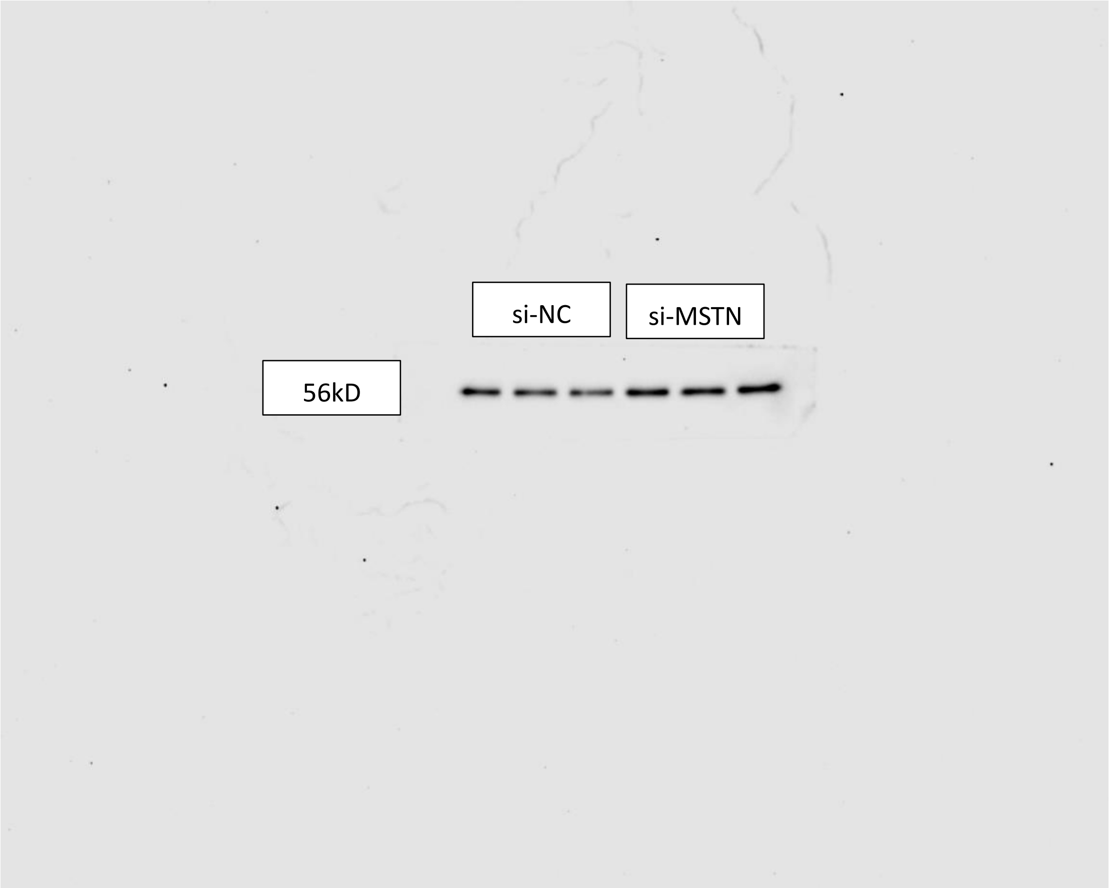

Figure 9-B, Western blotting results of AKT

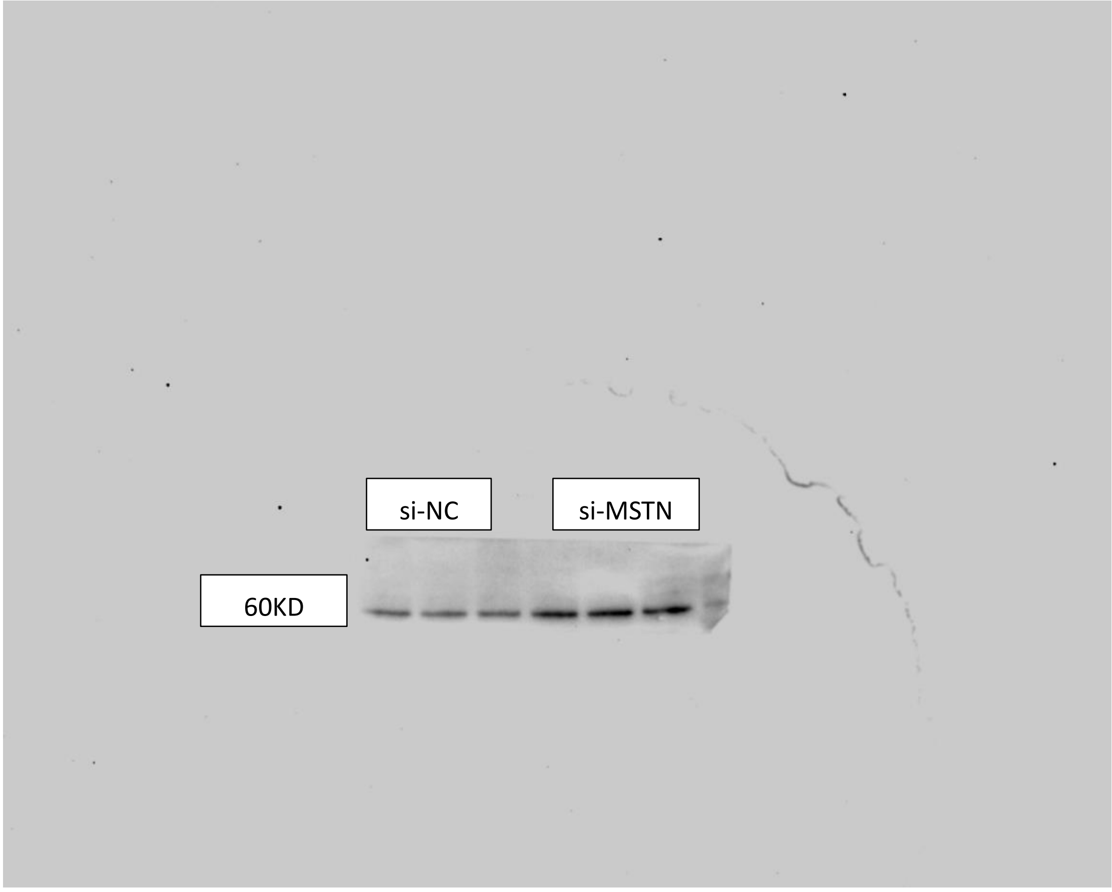

Figure 9-B, Western blotting results of p-AKT

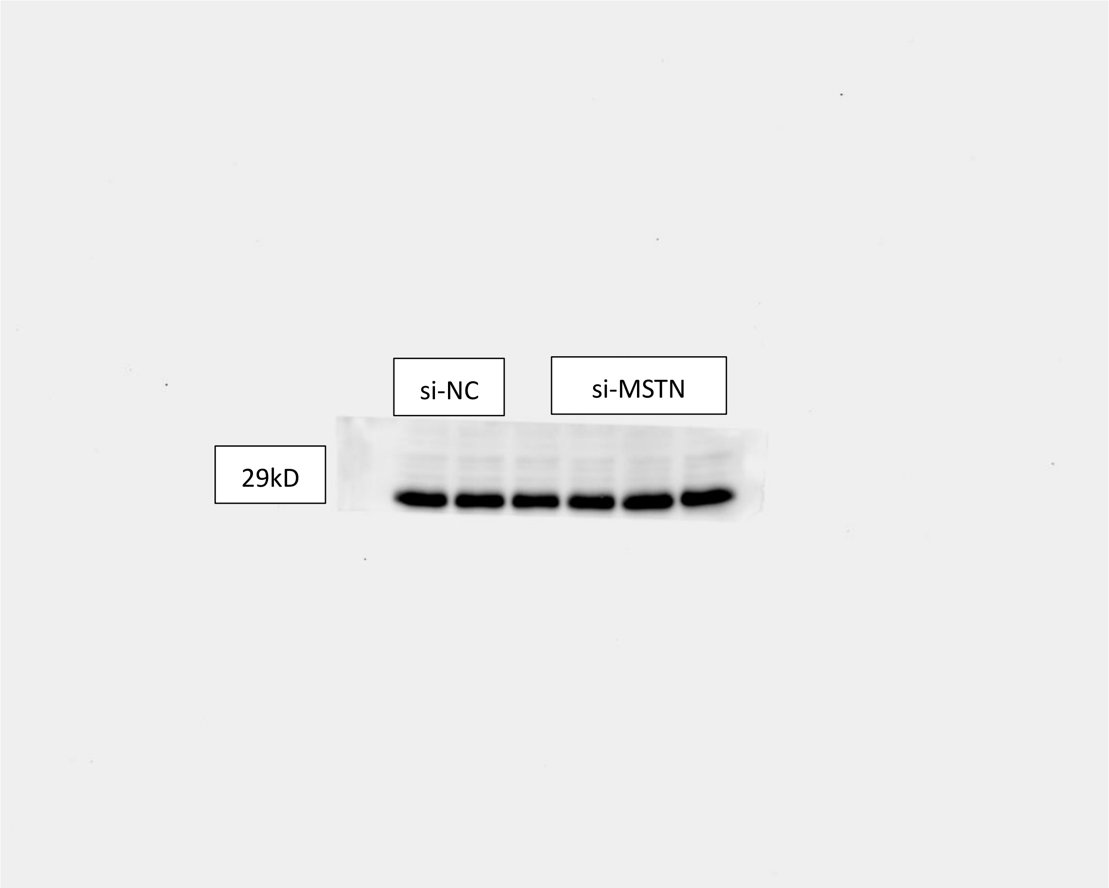

Figure 9-B, Western blotting results of RPS6

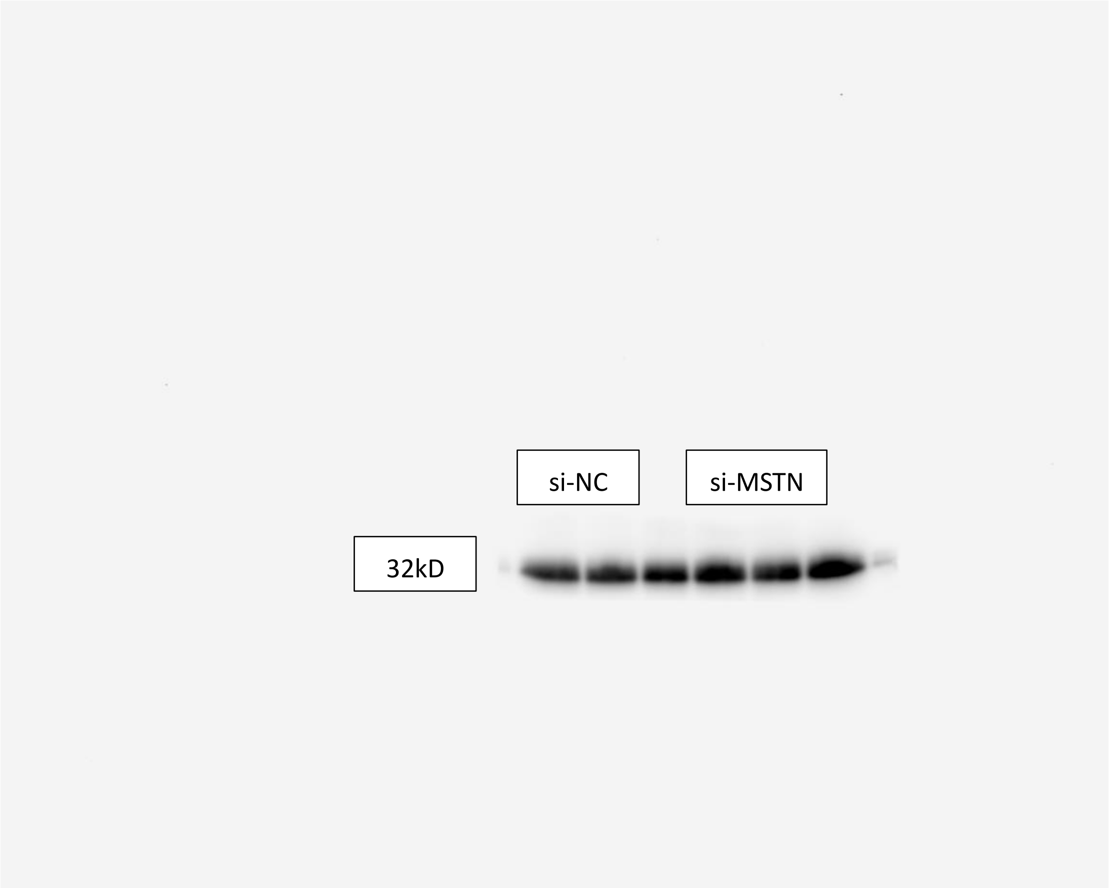

Figure 9-B, Western blotting results of p-RPS6

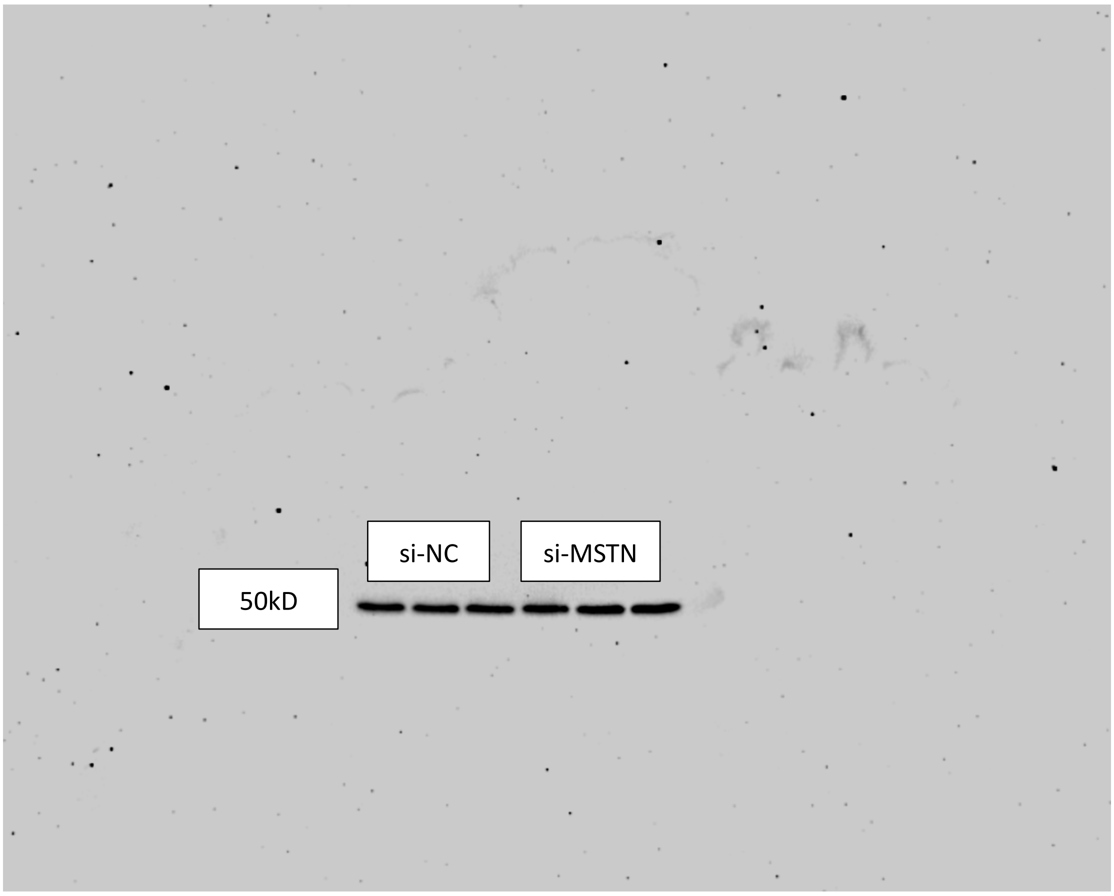

Figure 9-B, Western blotting results of α-tubulin

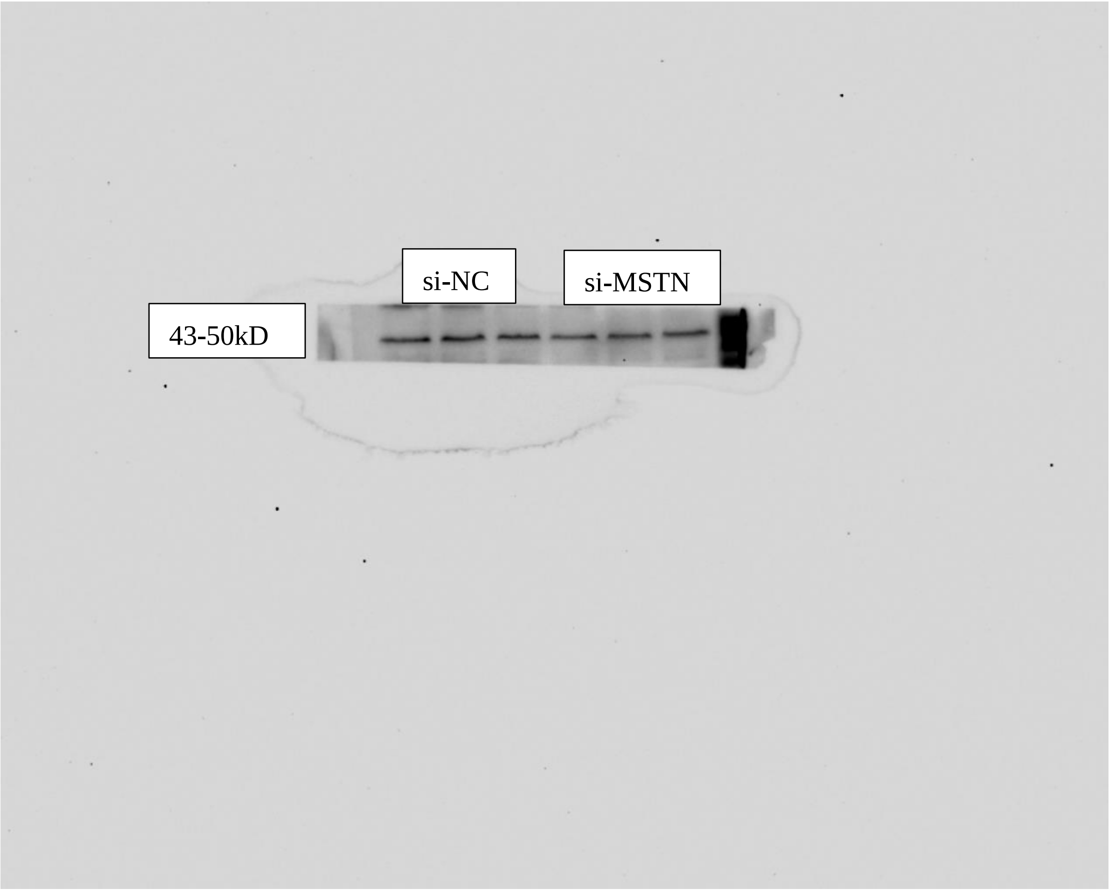

Figure 9-E, Western blotting results of MSTN

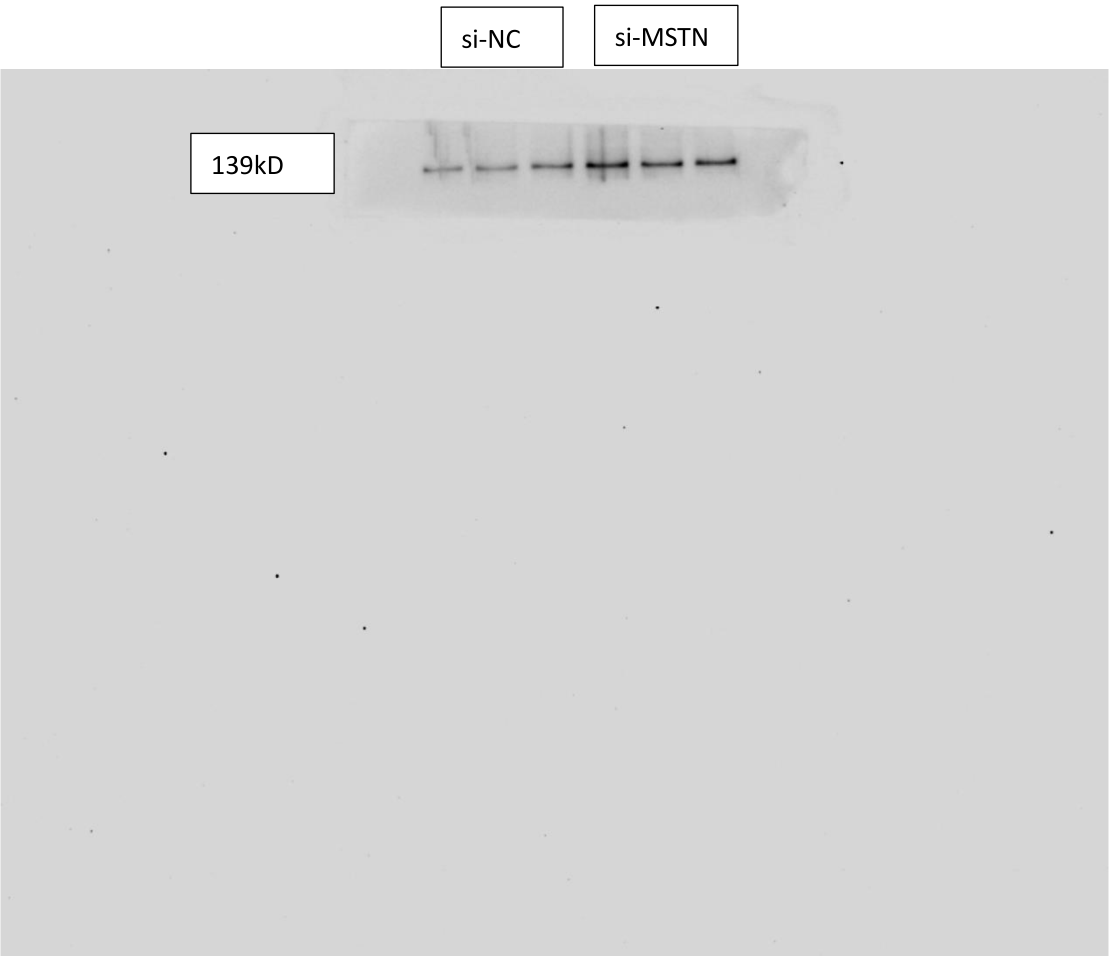

Figure 9-E, Western blotting results of COL1A1

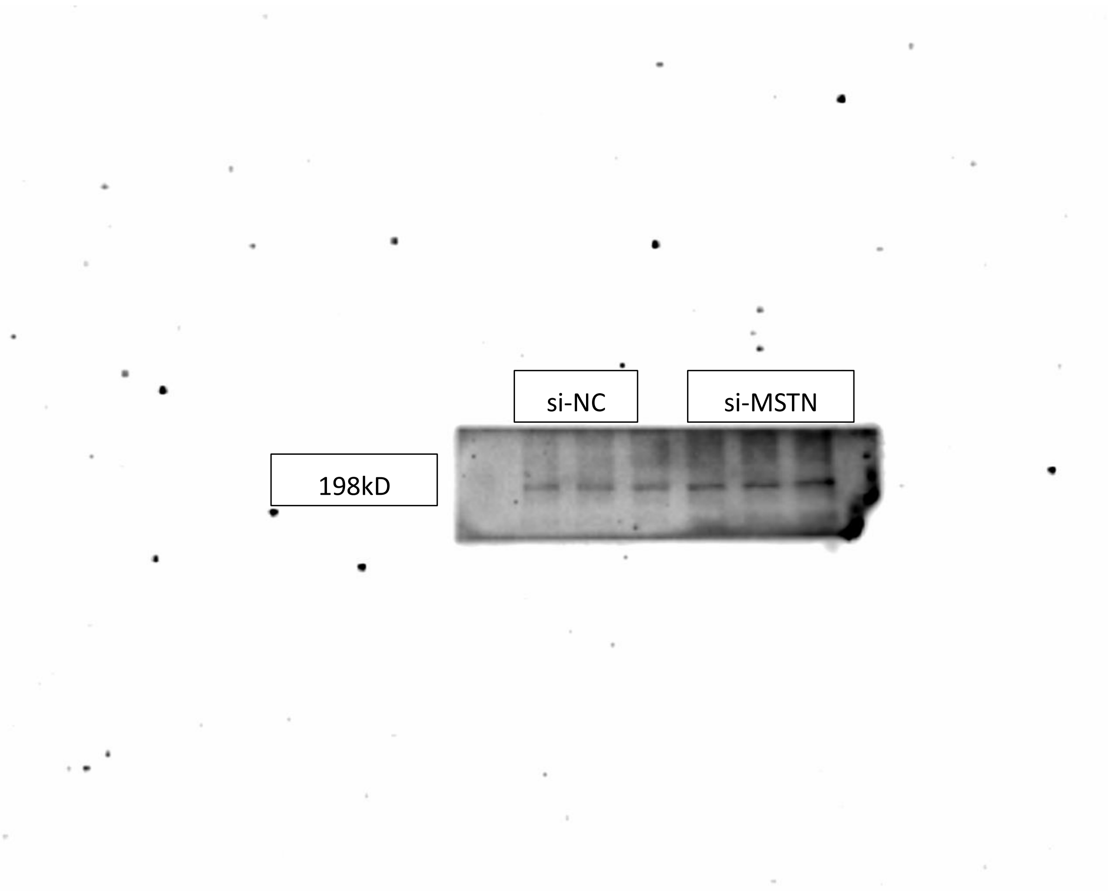

Figure 9-E, Western blotting results of LAMB1

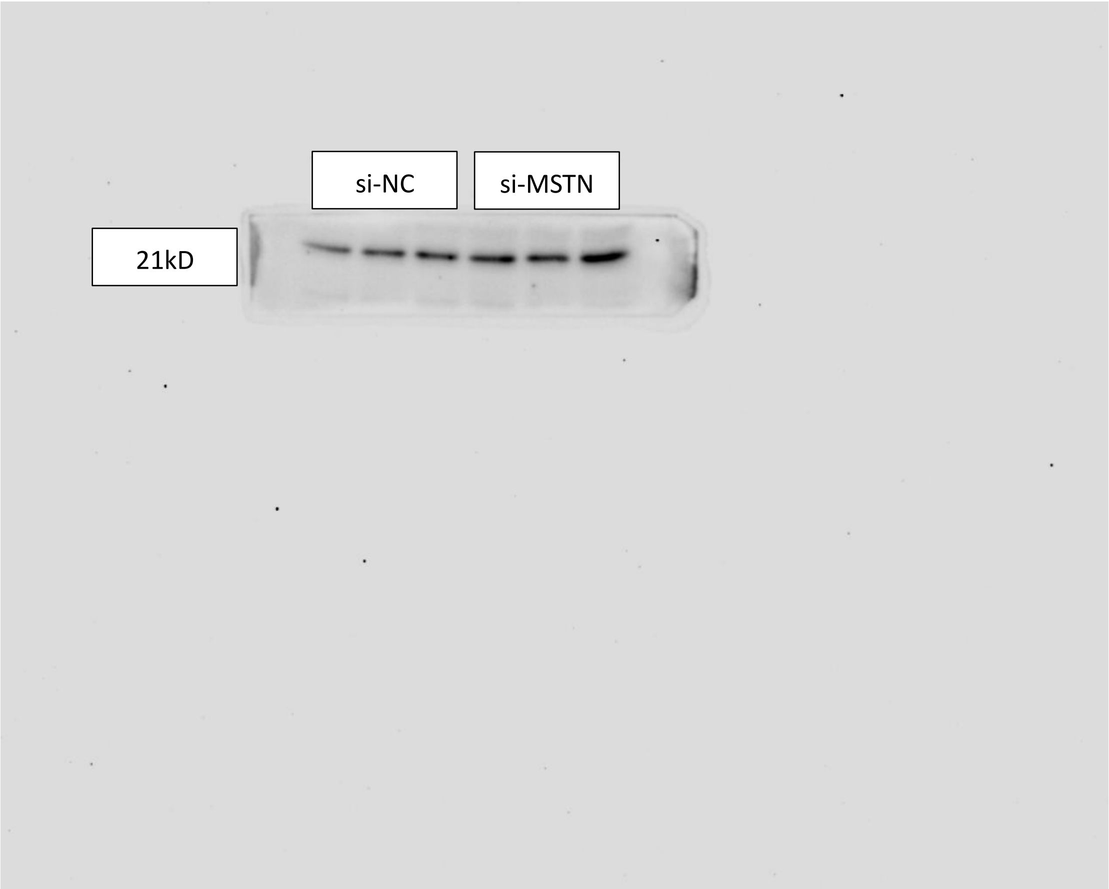

Figure 9-E, Western blotting results of Rac1

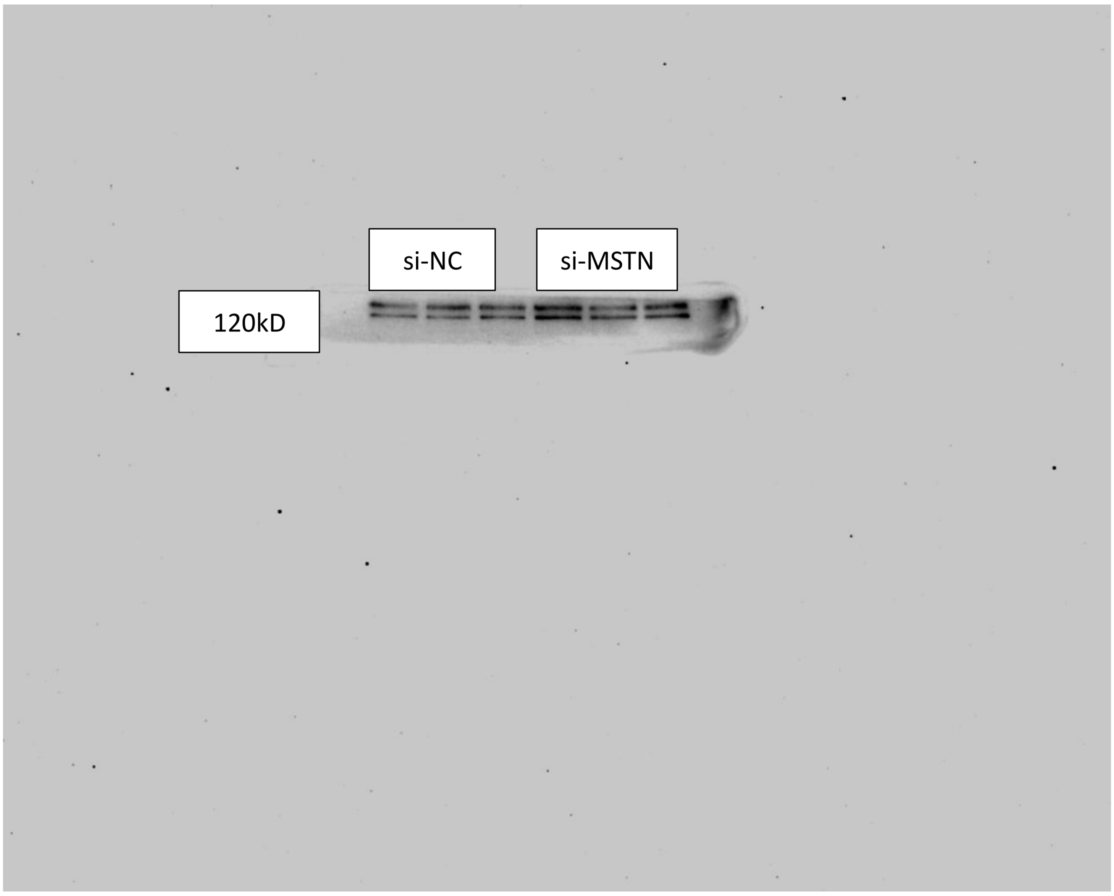

Figure 9-E, Western blotting results of FAK

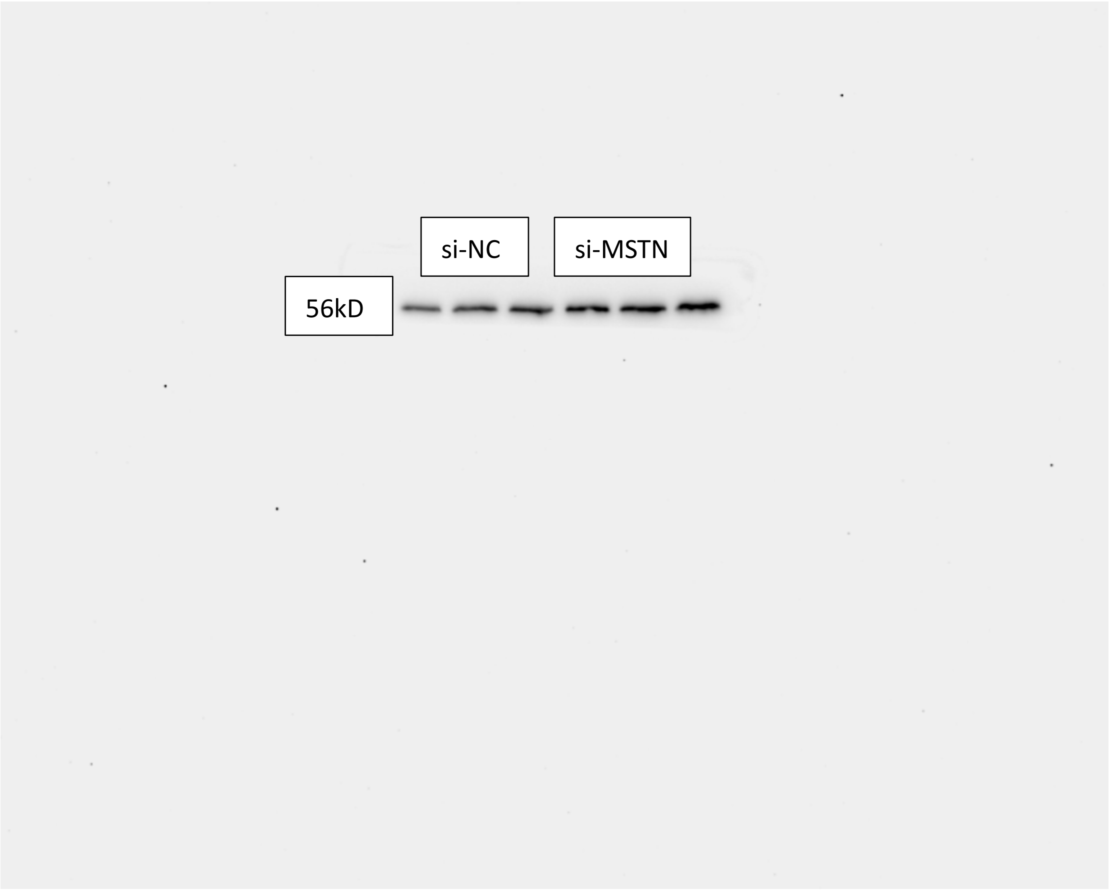

Figure 9-E, Western blotting results of AKT

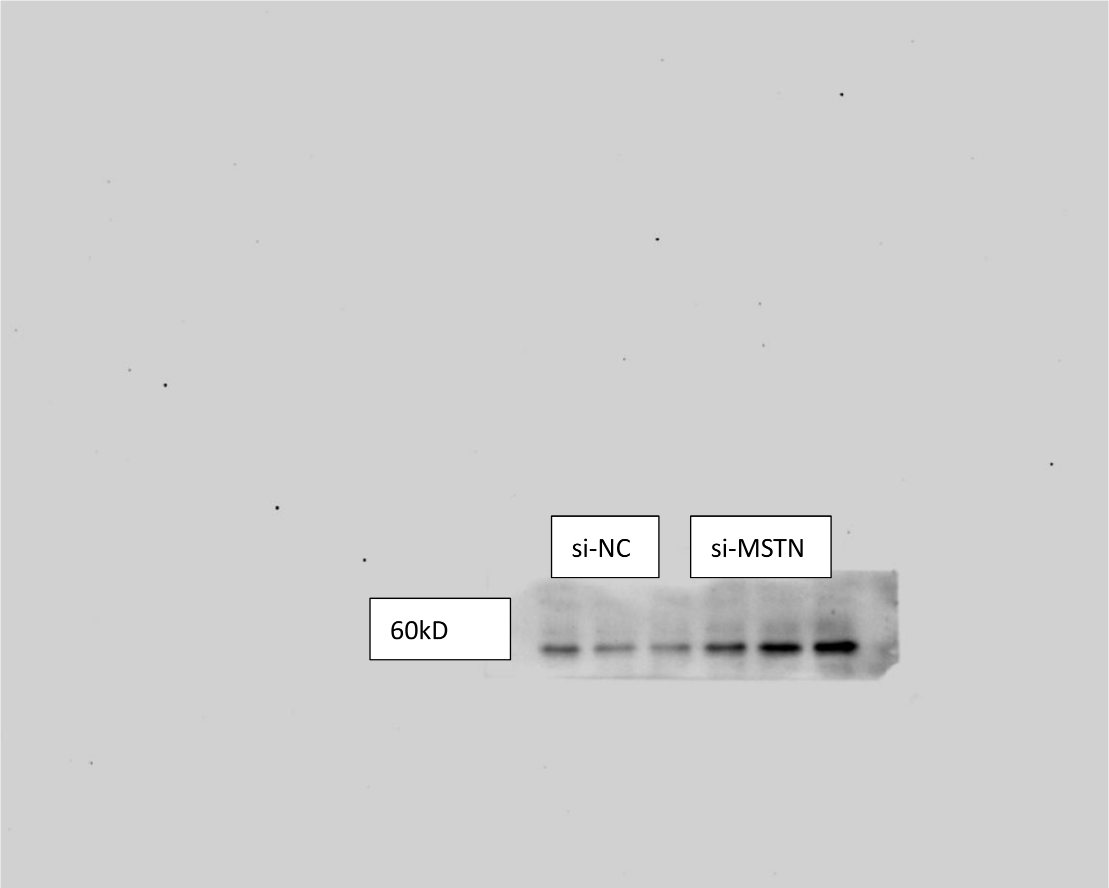

Figure 9-E, Western blotting results of p-AKT

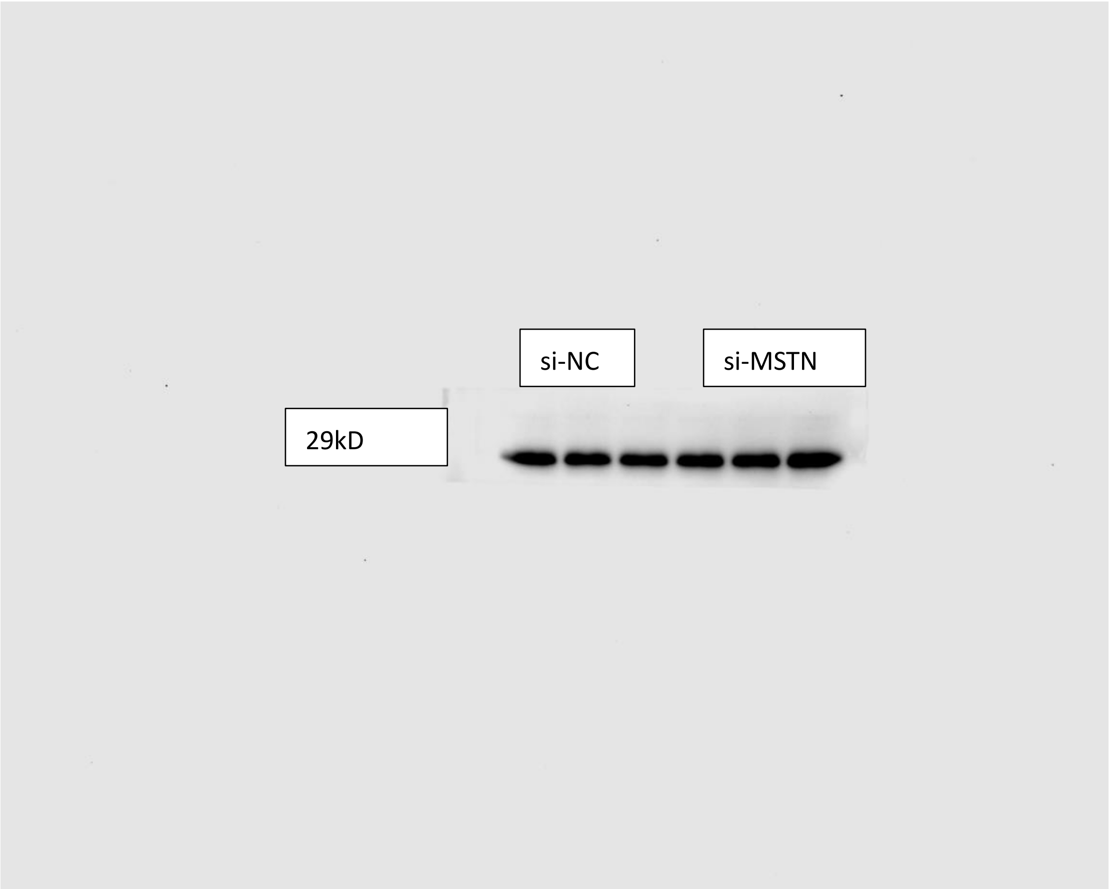

Figure 9-E, Western blotting results of RPS6

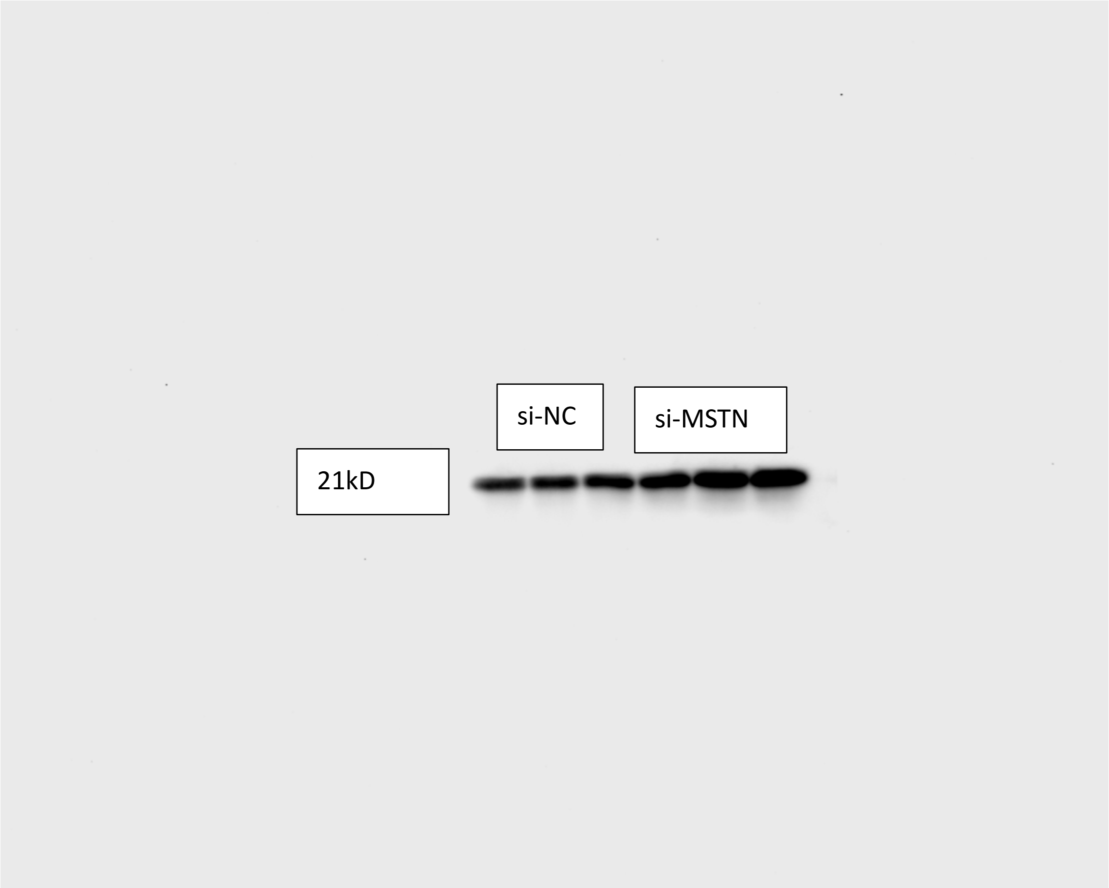

Figure 9-E, Western blotting results of p-RPS6

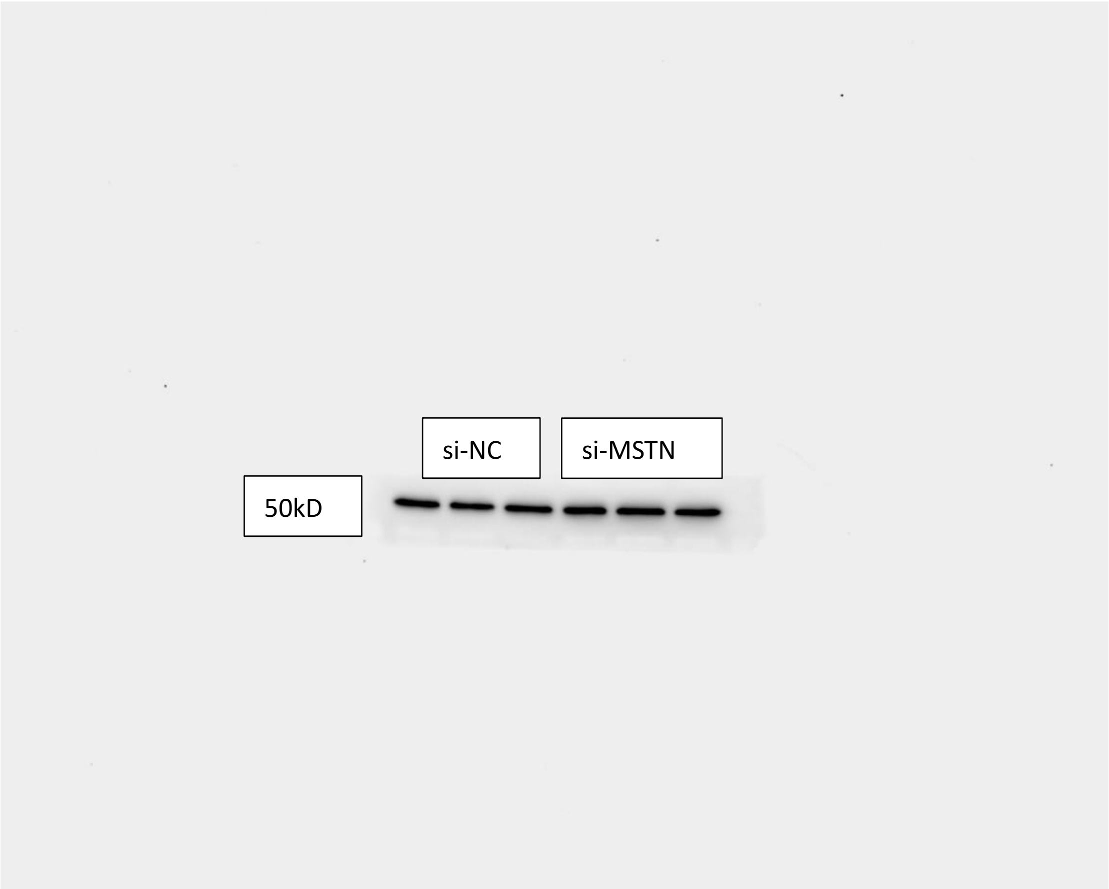

Figure 9-E, Western blotting results of α-tubulin

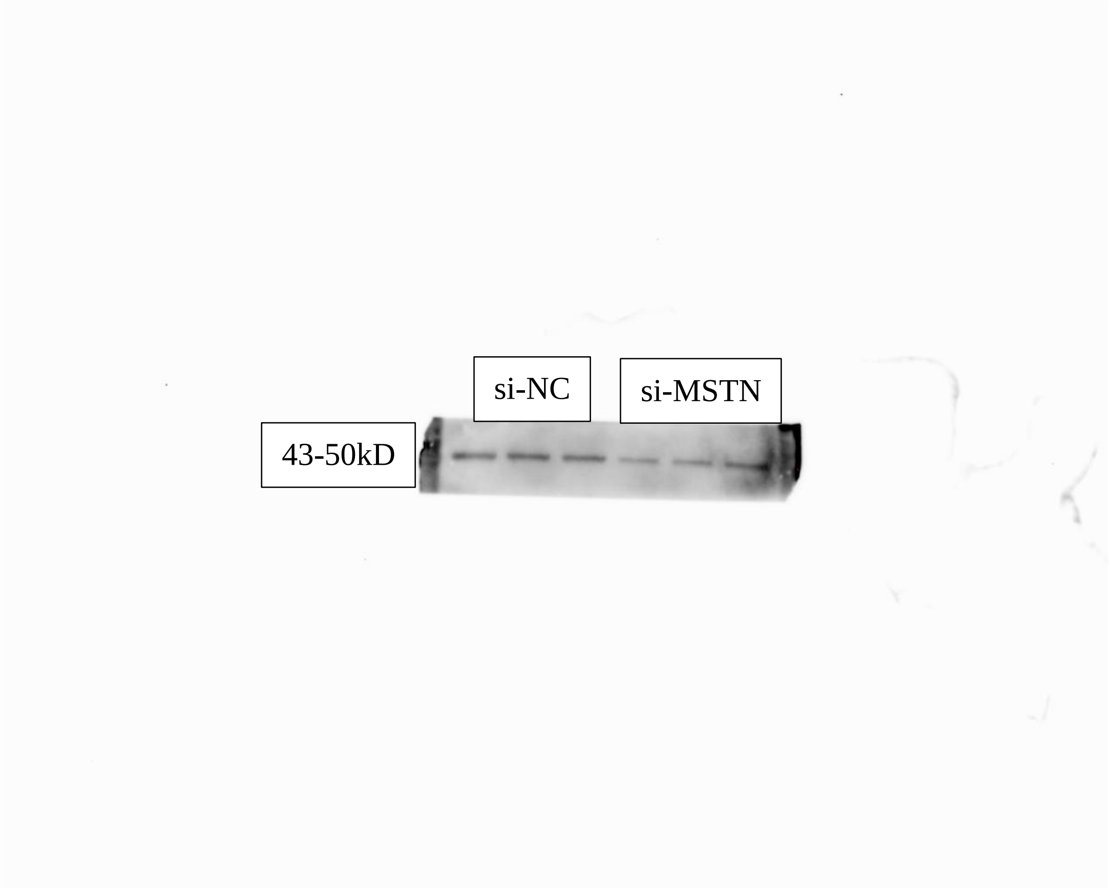

Figure 10-B, Western blotting results of MSTN

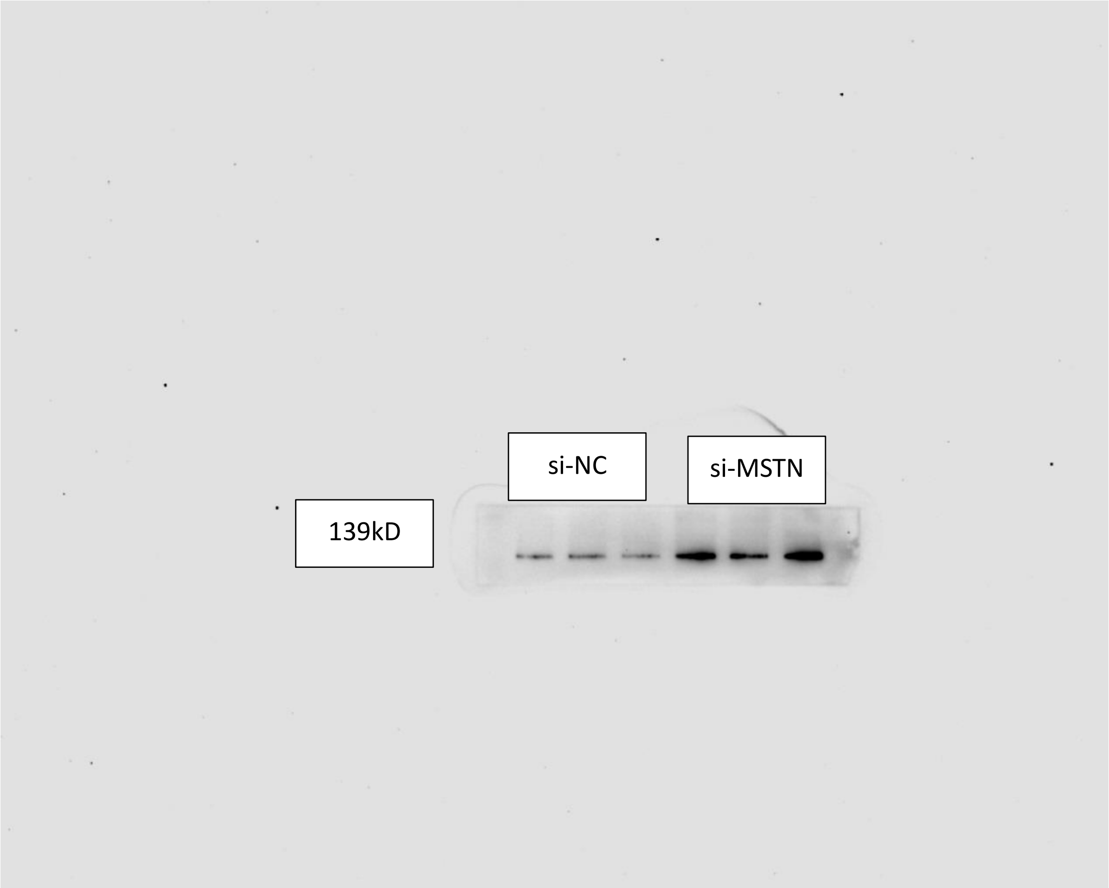

Figure 10-B, Western blotting results of COL1A1

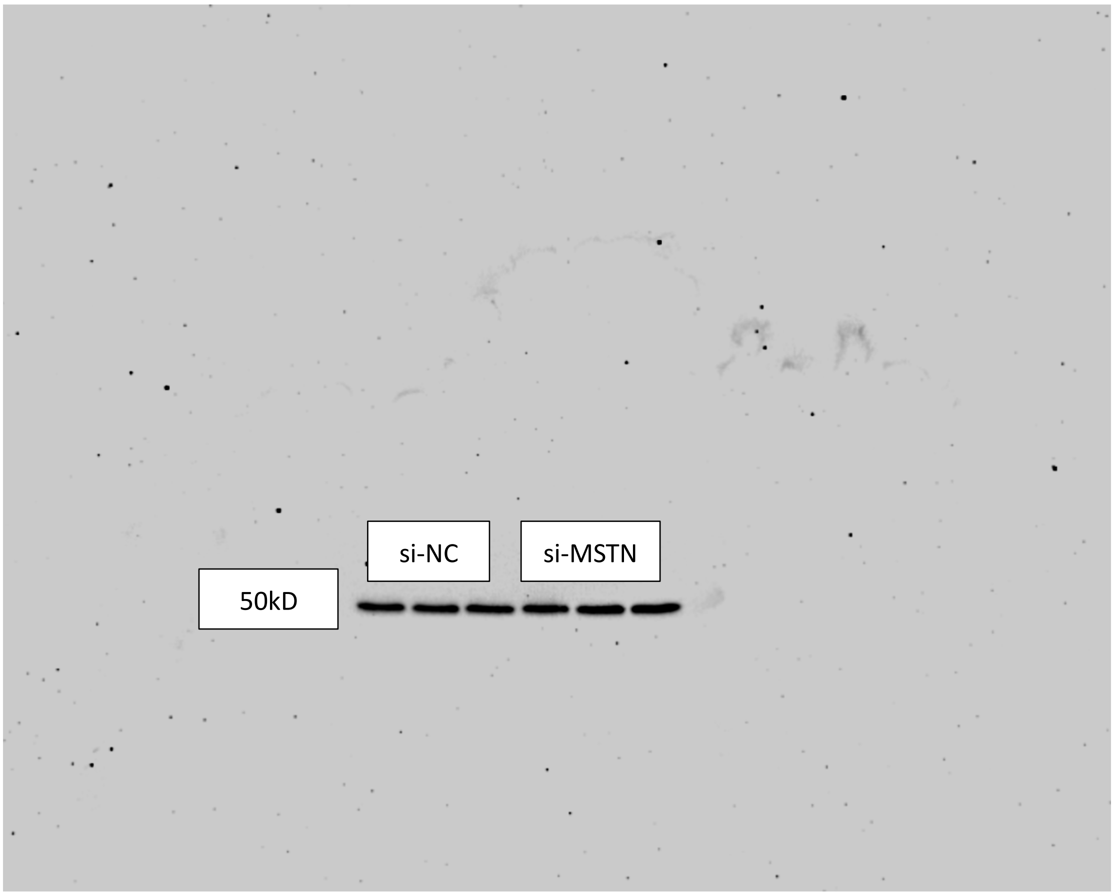

Figure 10-B, Western blotting results of α-tubulin

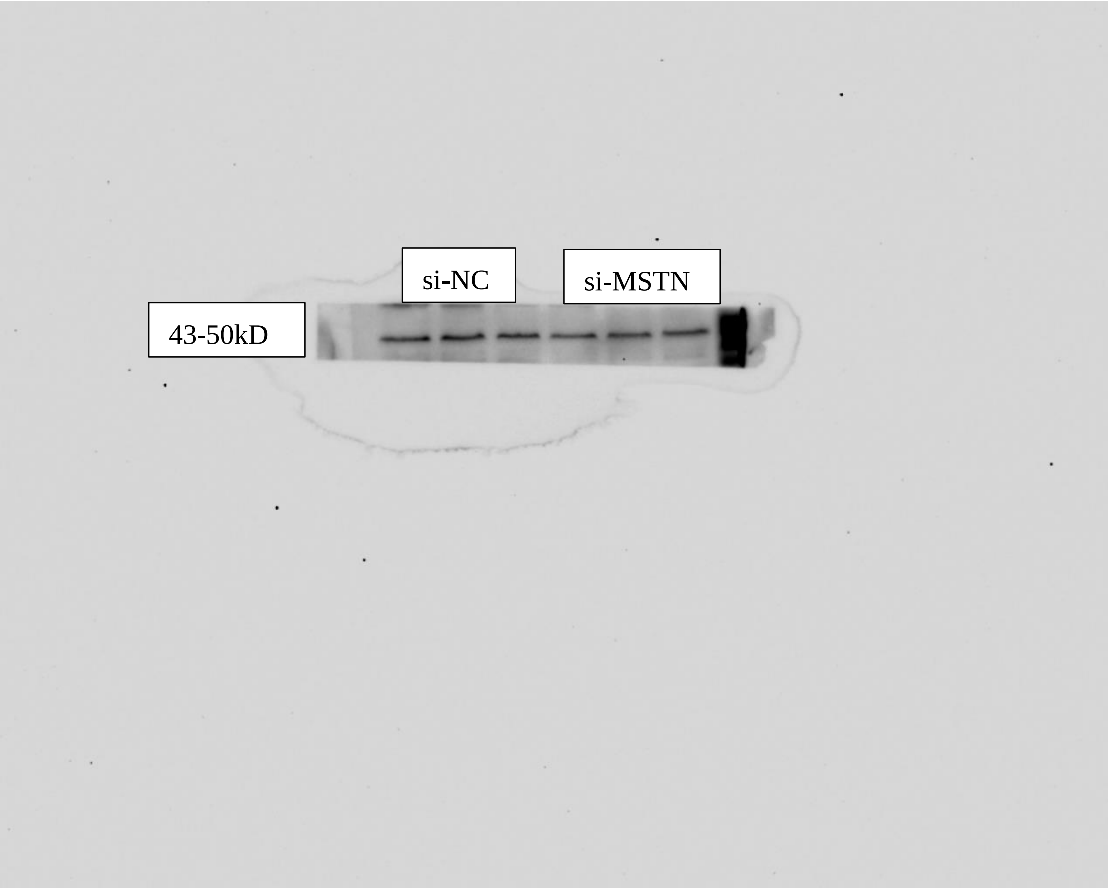

Figure 10-C, Western blotting results of MSTN

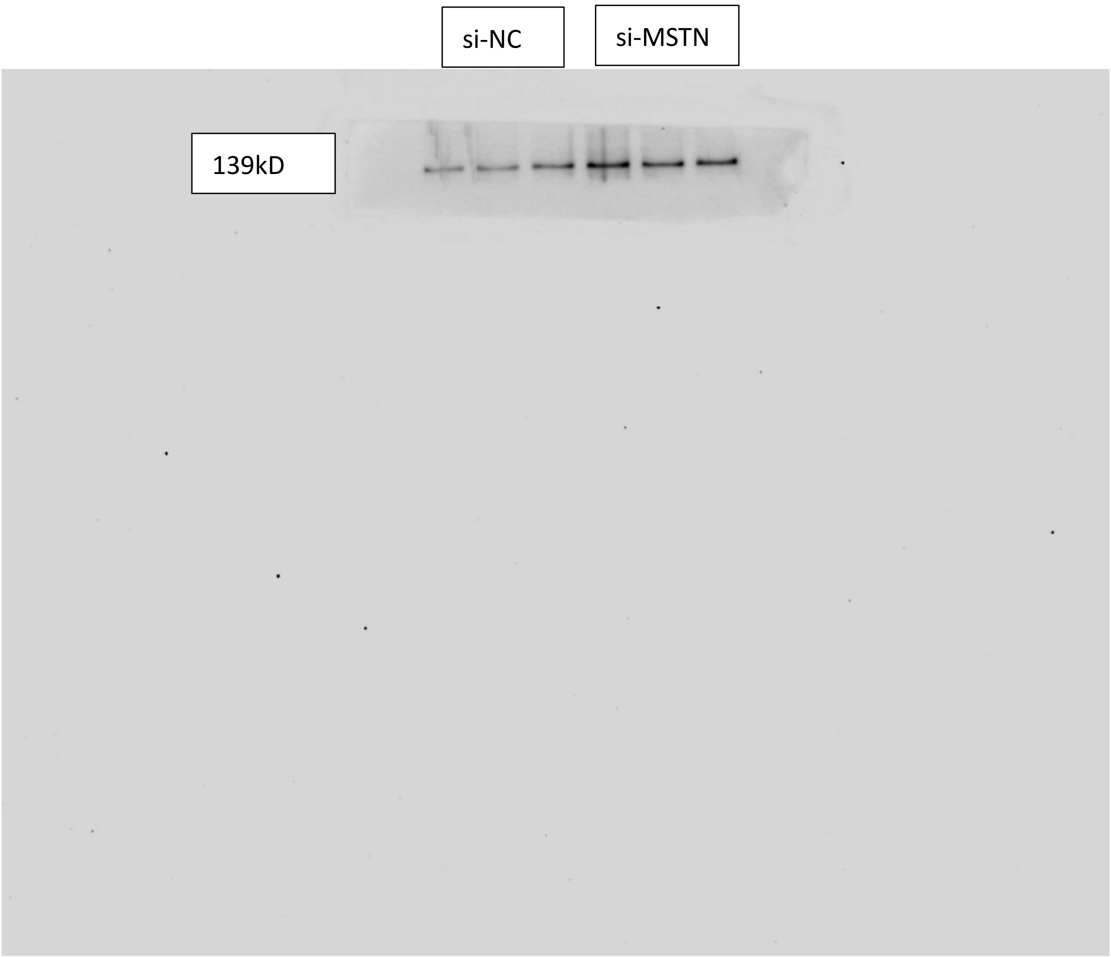

Figure 10-C, Western blotting results of COL1A1

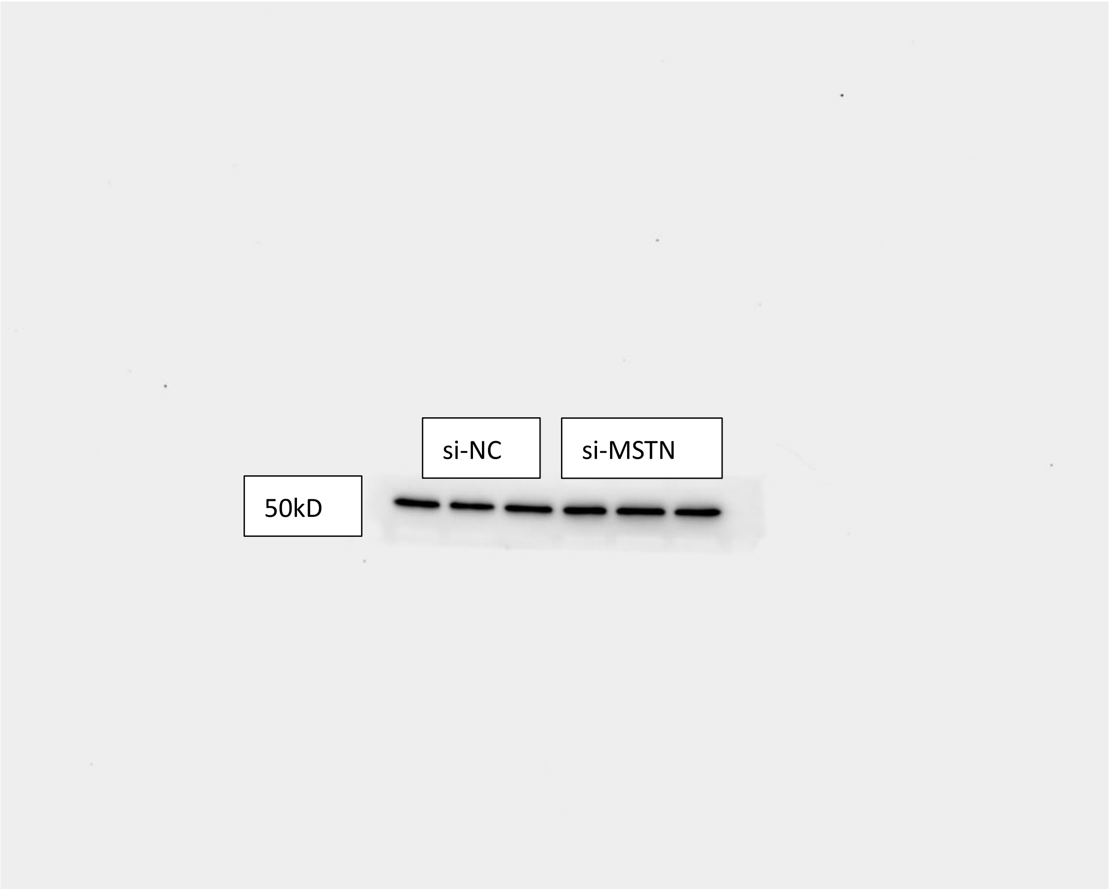

Figure 10-C, Western blotting results of α-tubulin

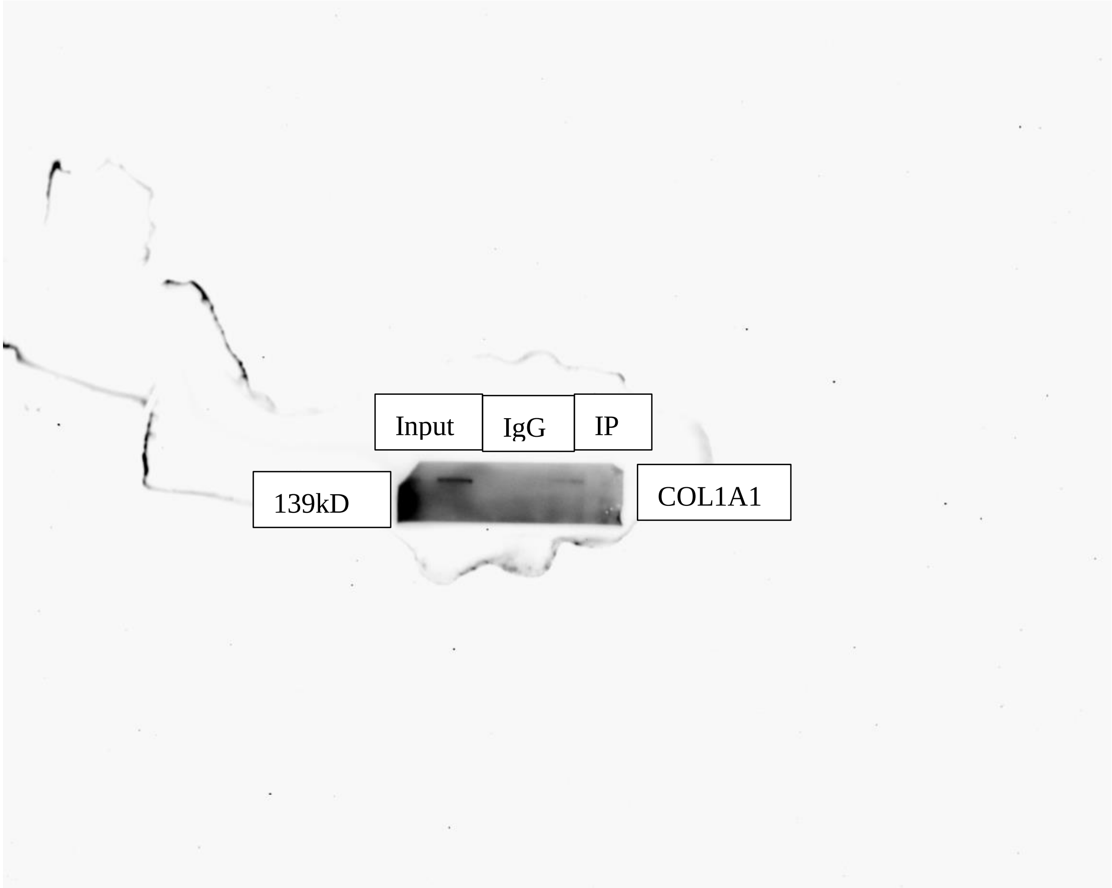

Figure 10-D, Western blotting results of COL1A1

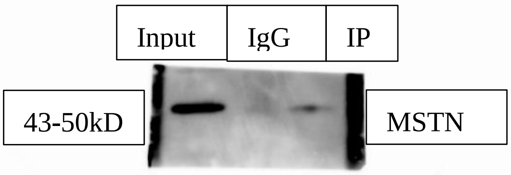

Figure 10-D, Western blotting results of MSTN

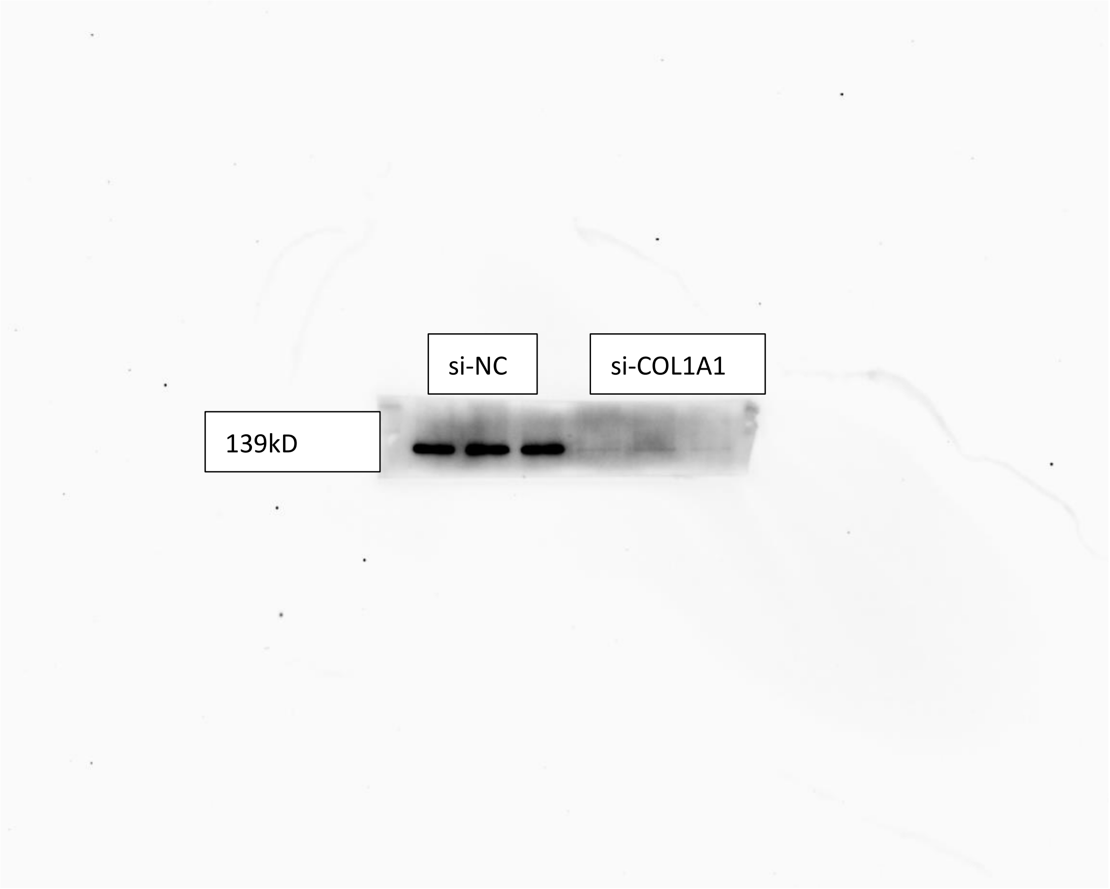

Figure 11-C, Western blotting results of COL1A1

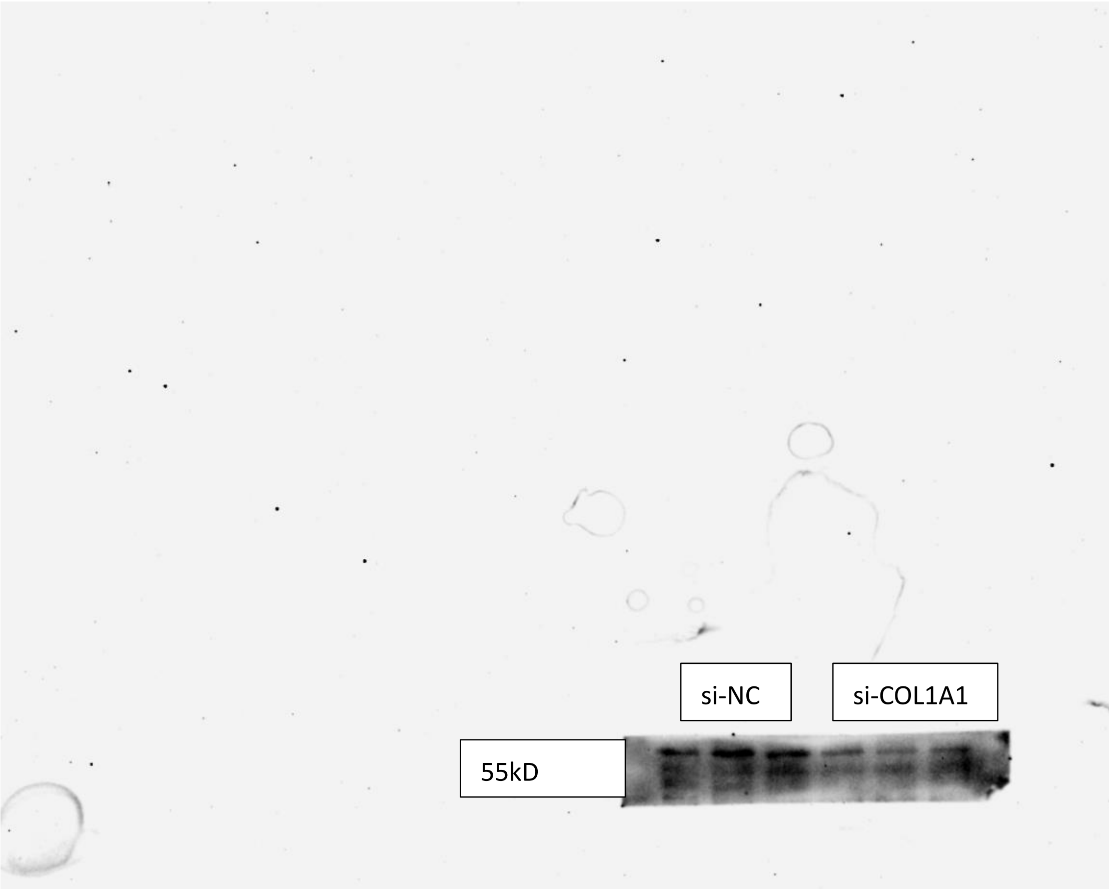

Figure 11-C, Western blotting results of Pax7

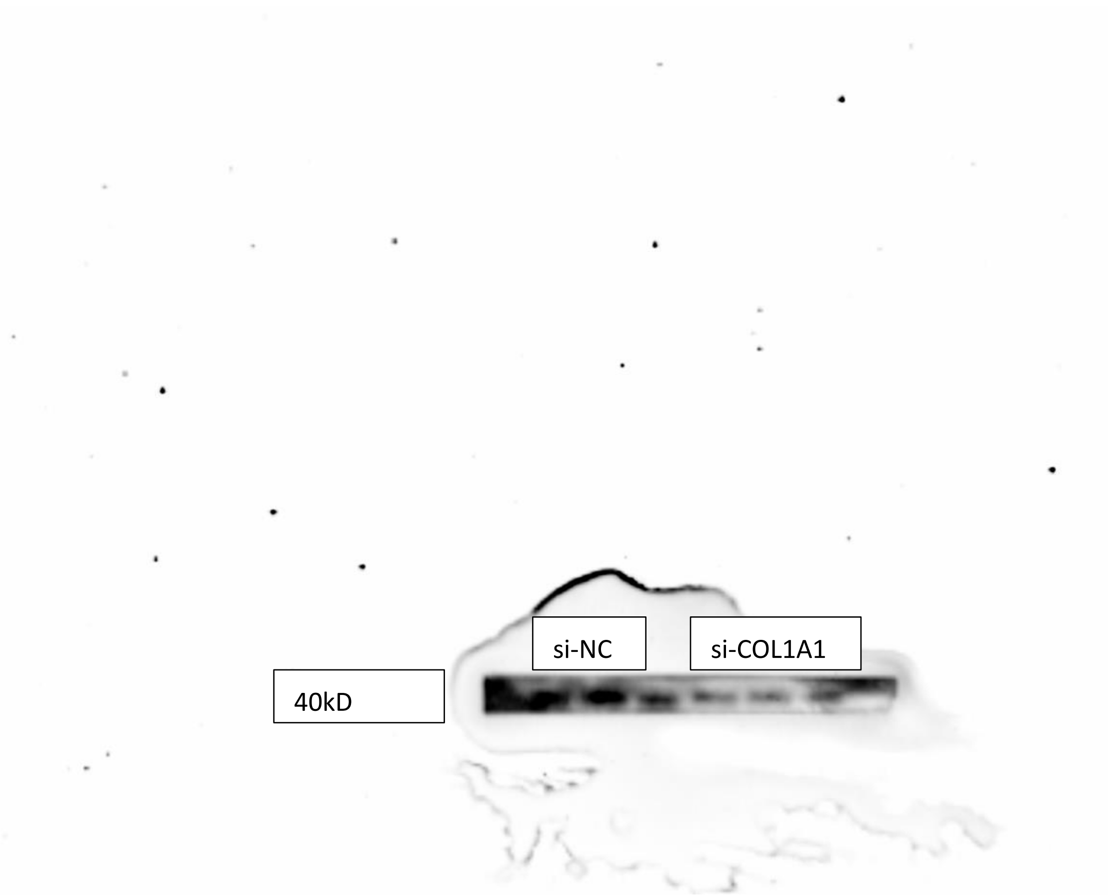

Figure 11-C, Western blotting results of MyoD

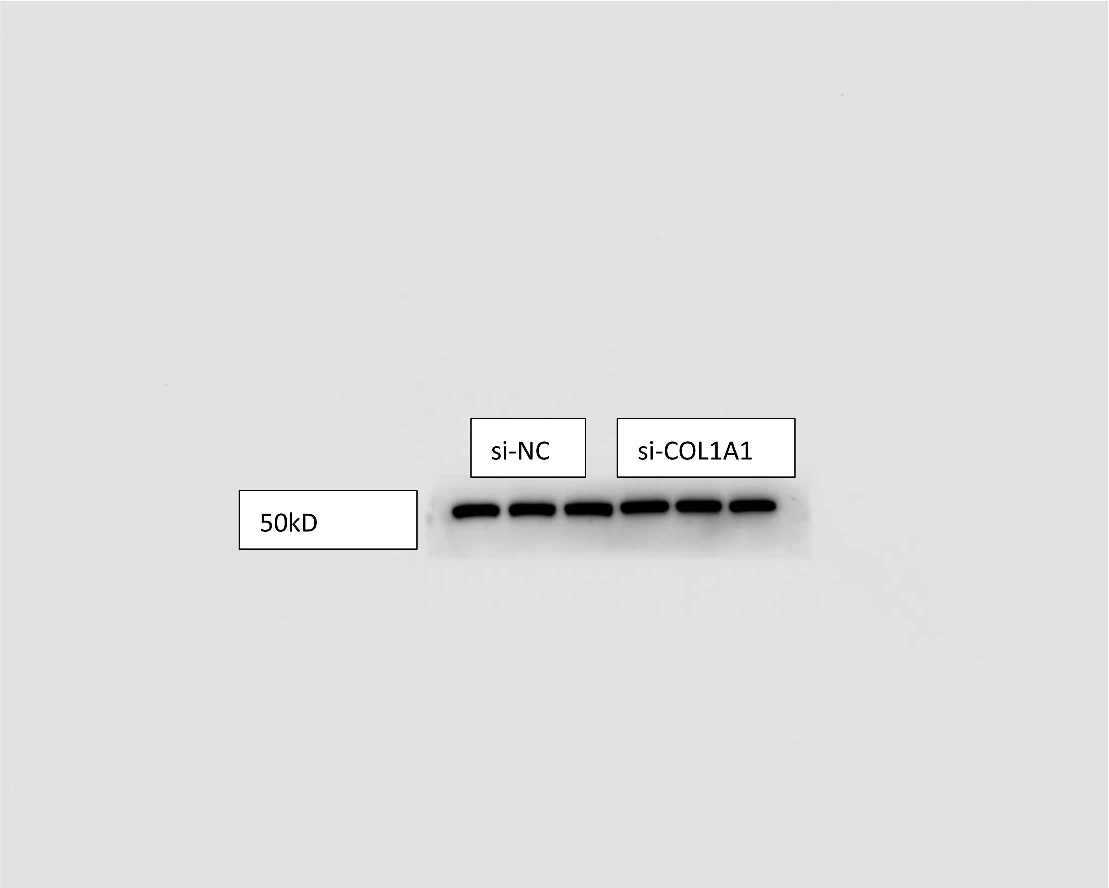

Figure 11-C, Western blotting results of α-tubulin

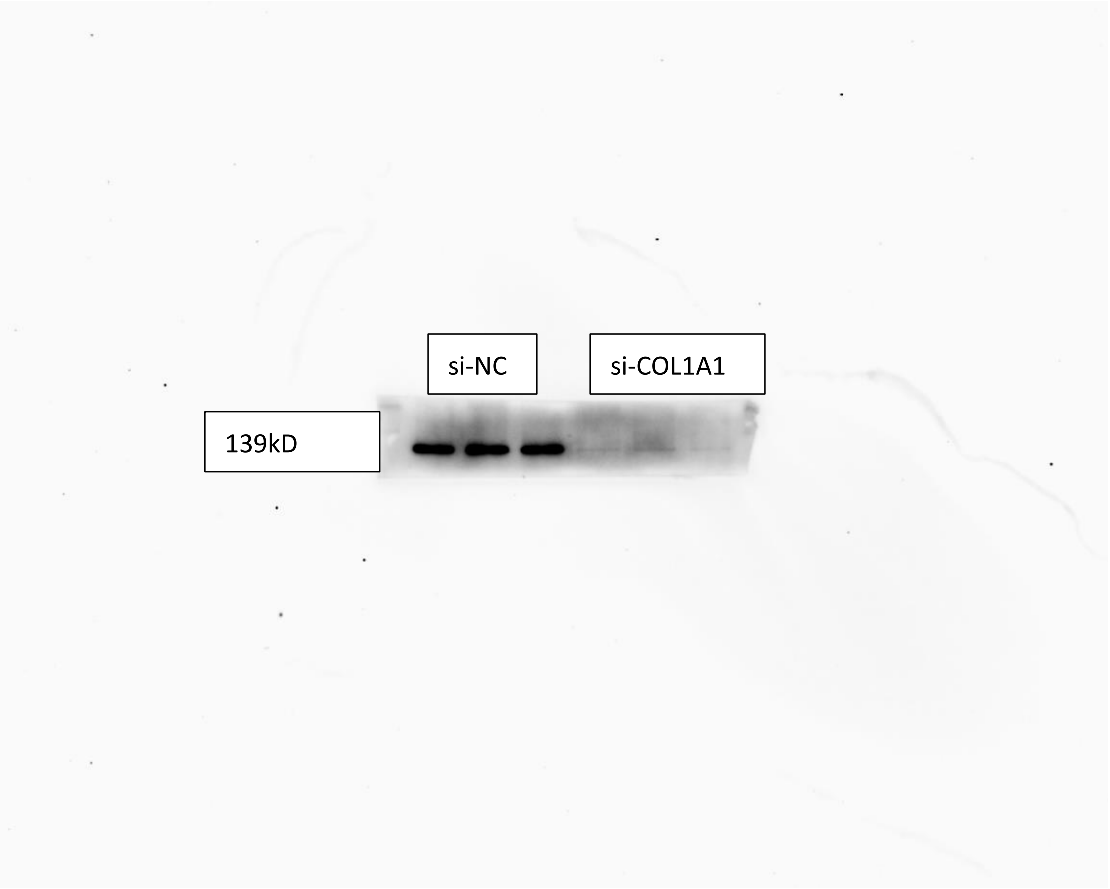

Figure 12-C, Western blotting results of COL1A1

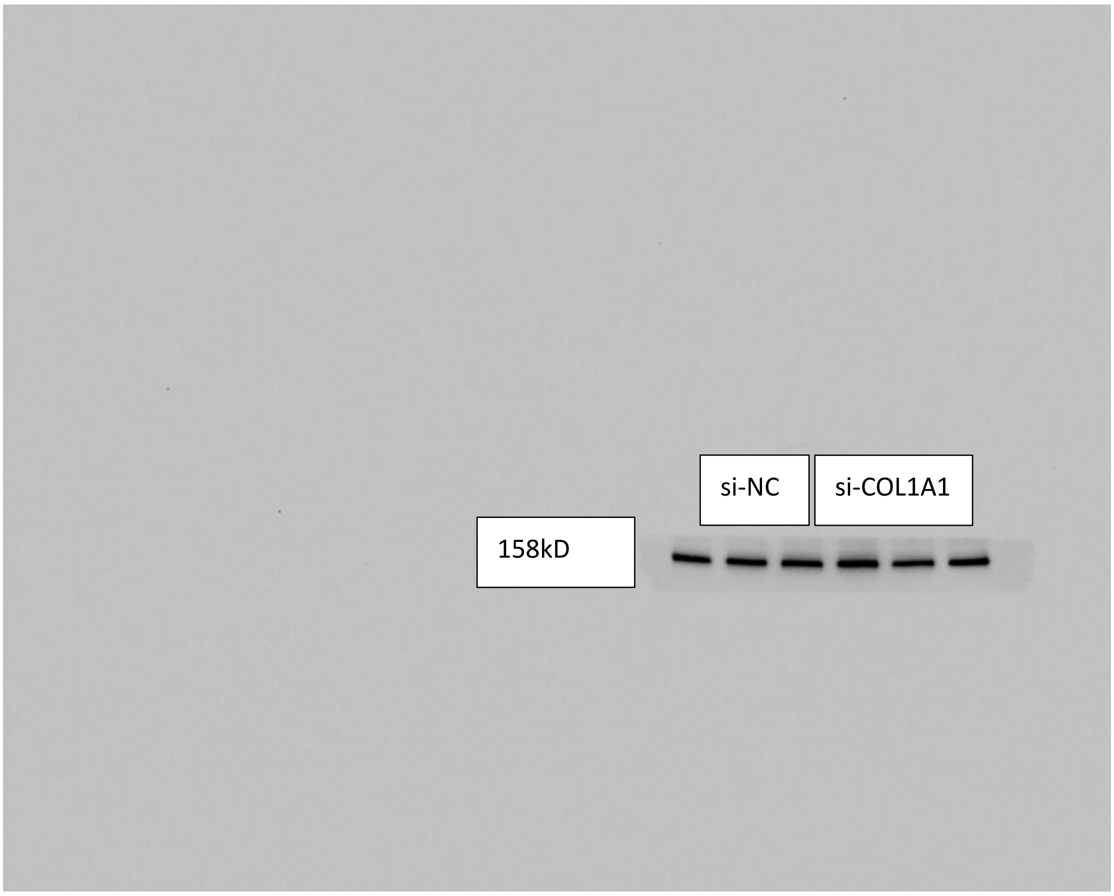

Figure 12-C, Western blotting results of Rock1

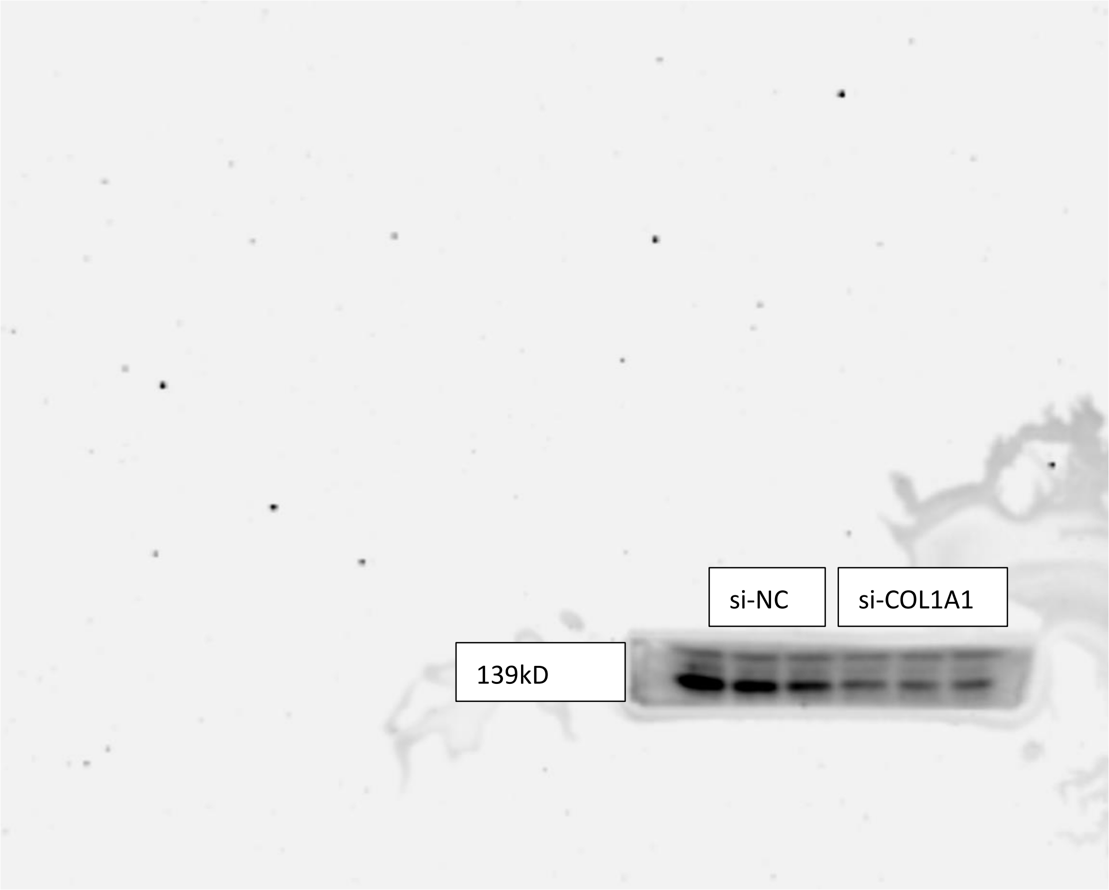

Figure 12-C, Western blotting results of Rac1

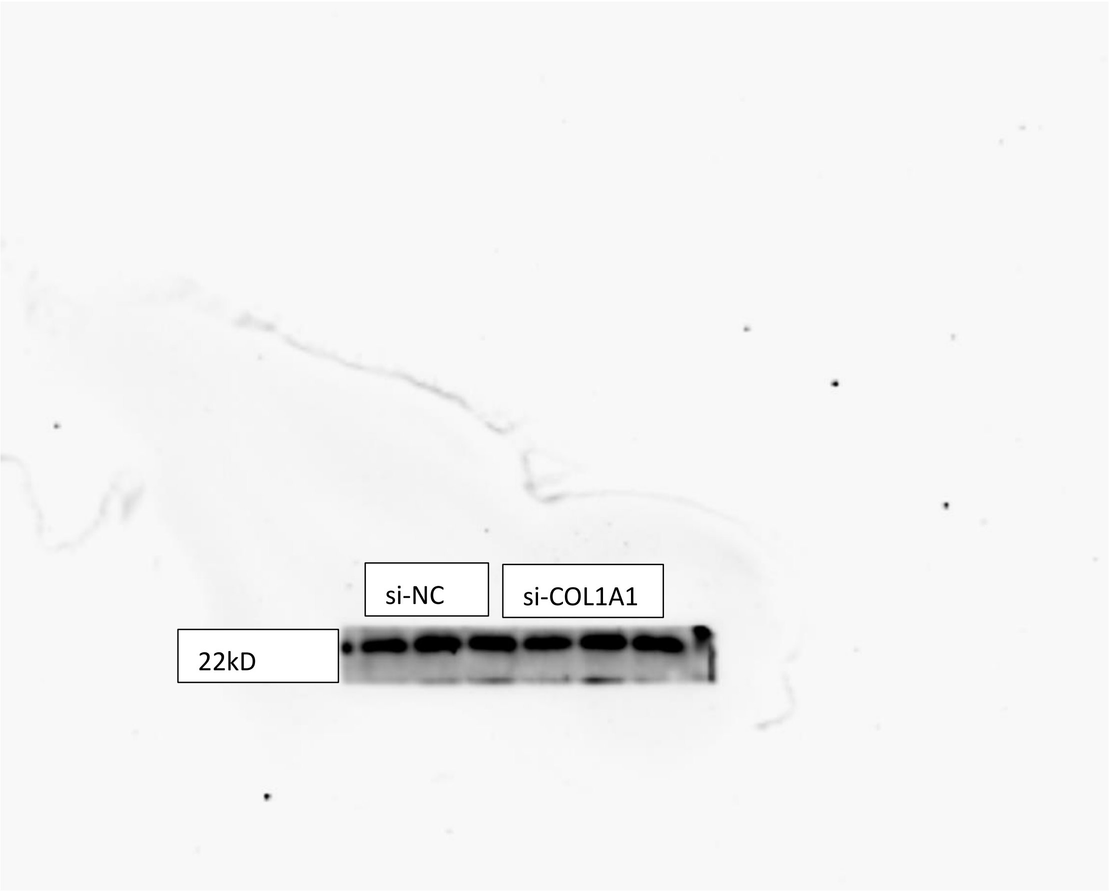

Figure 12-C, Western blotting results of RhoA

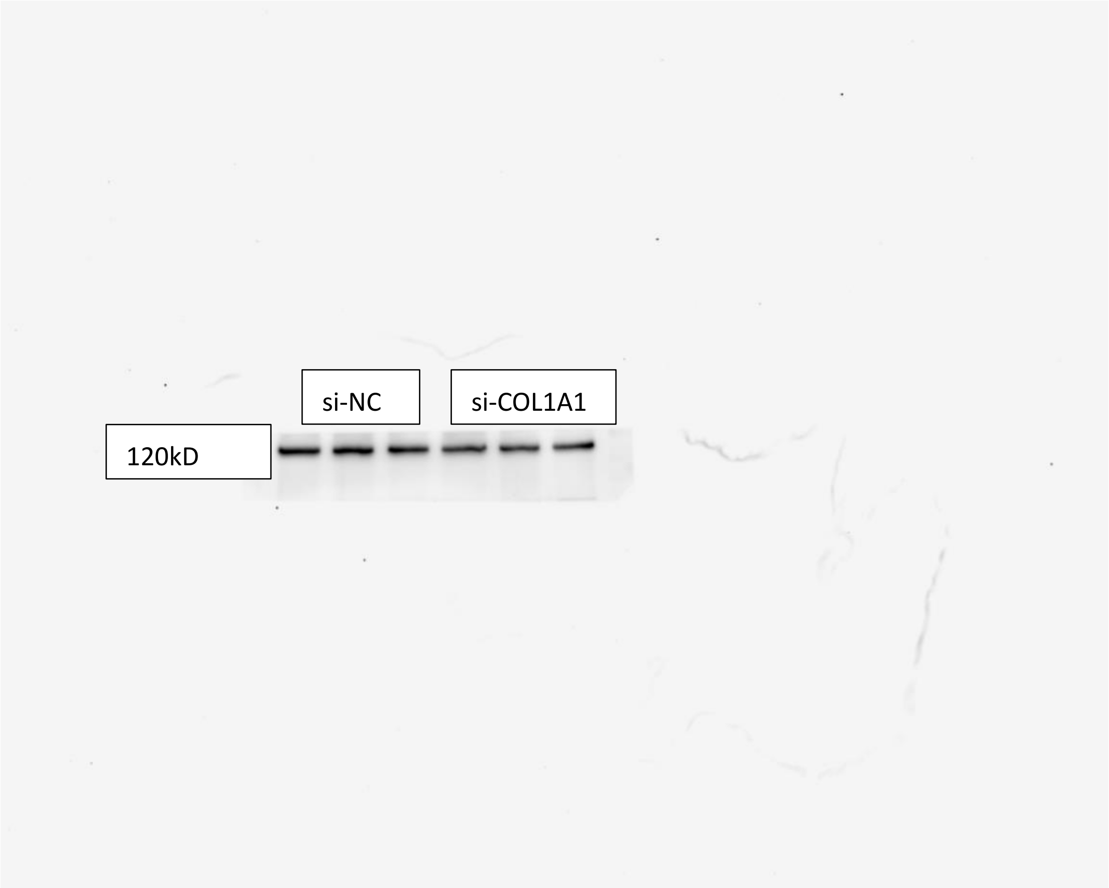

Figure 12-C, Western blotting results of FAK

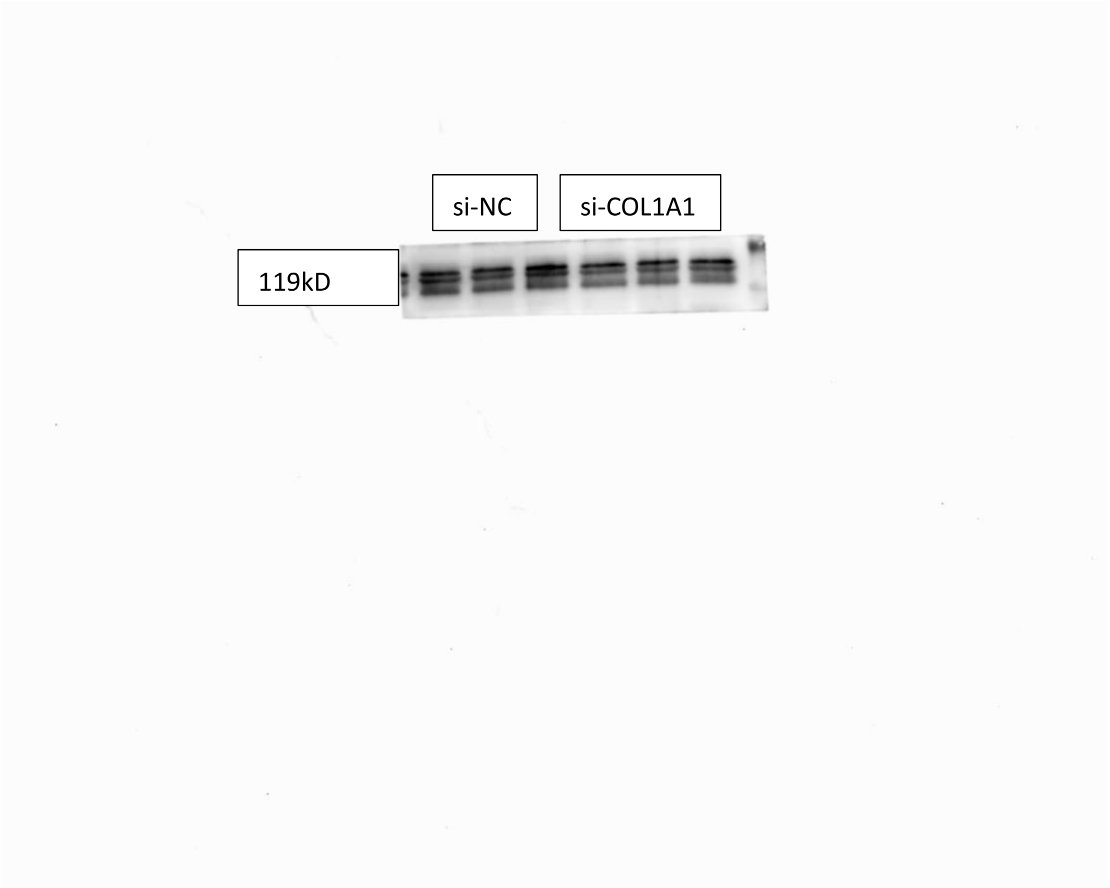

Figure 12-C, Western blotting results of p-FAK

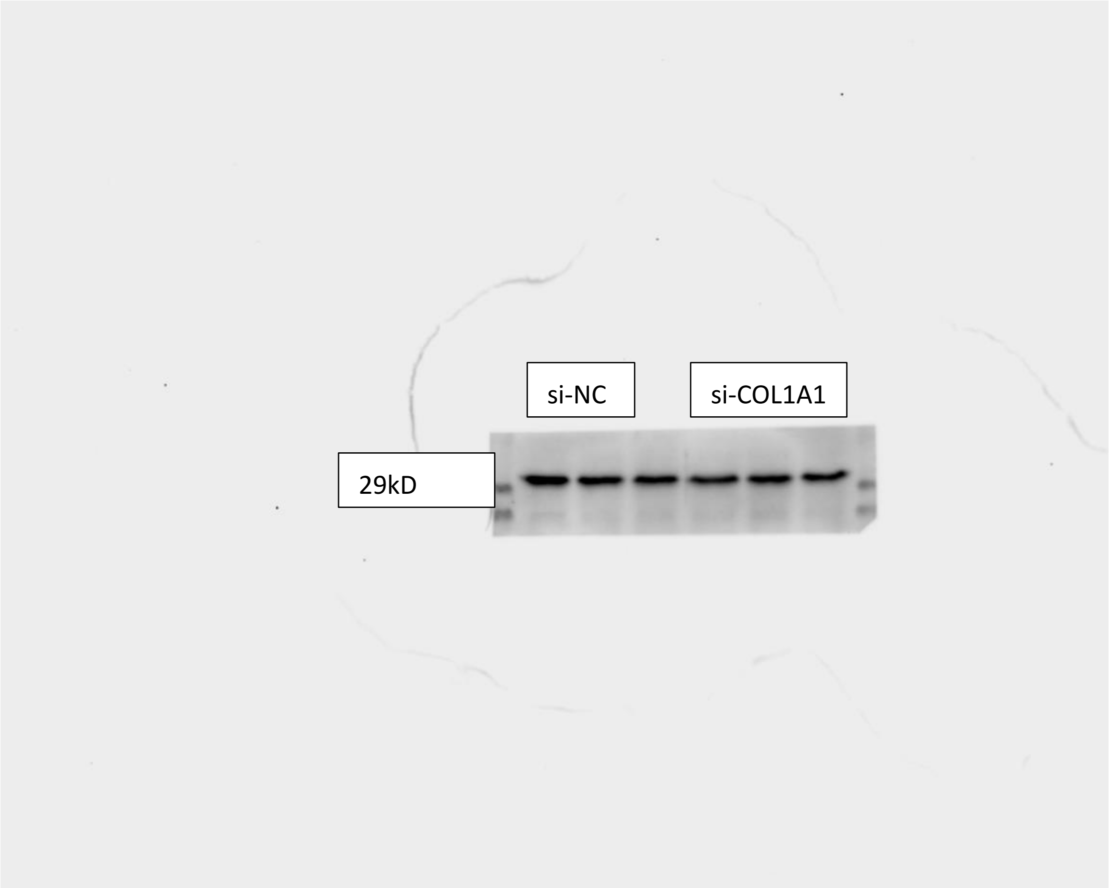

Figure 12-C, Western blotting results of RPS6

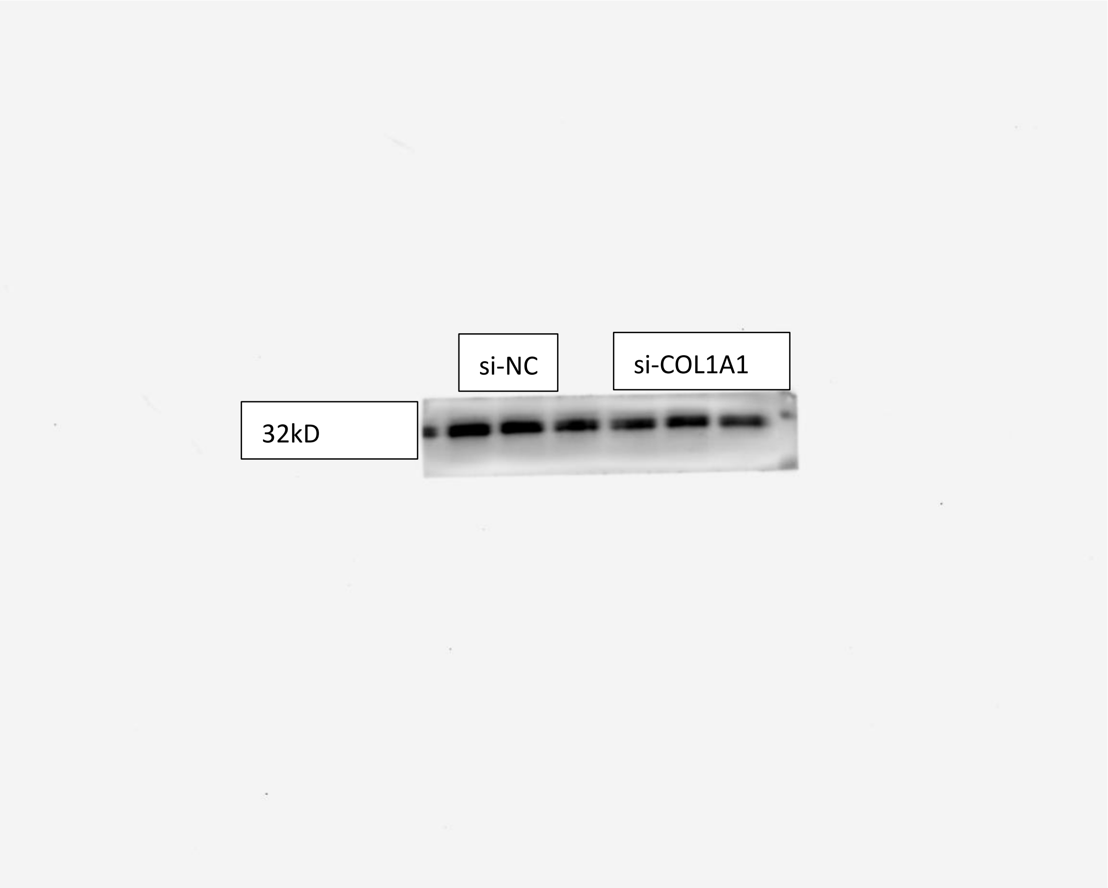

Figure 12-C, Western blotting results of p-RPS6

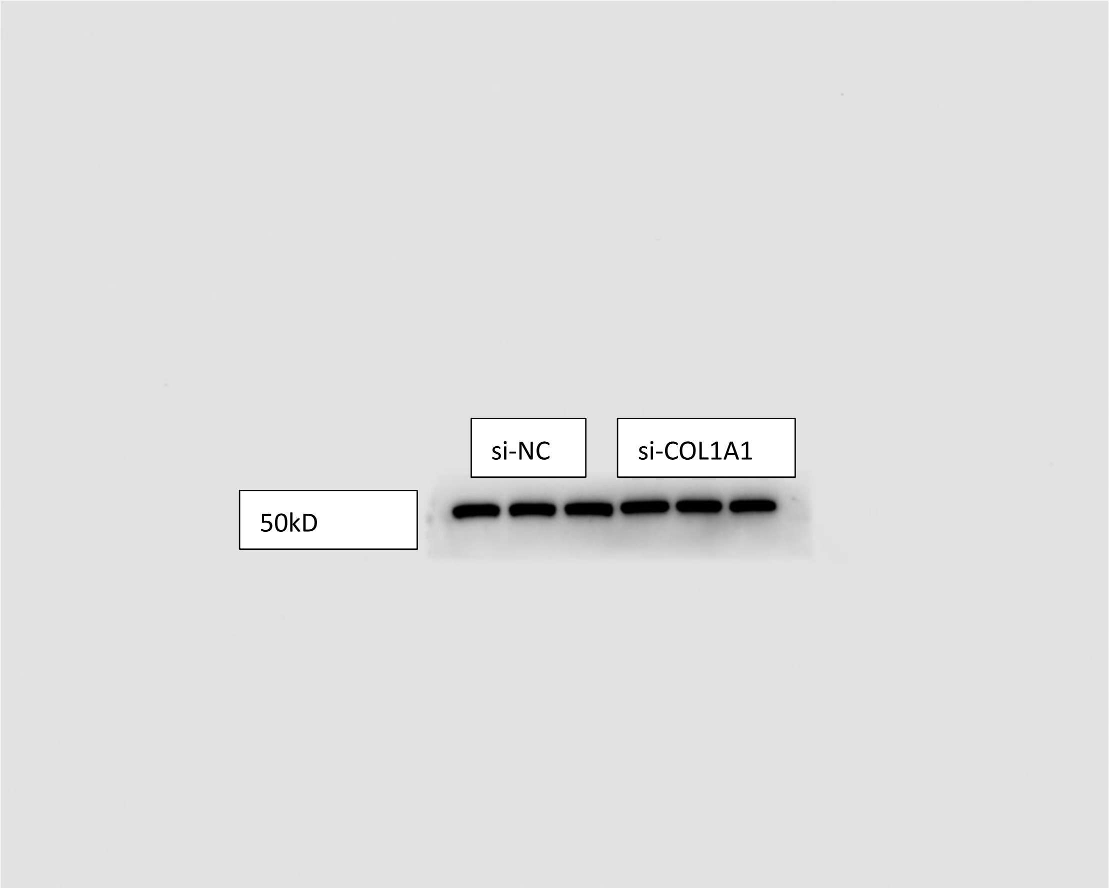

Figure 12-C, Western blotting results of α-tubulin
